# p.E95K mutation in Indian hedgehog causing brachydactyly type A1 impairs IHH/Gli1 downstream transcriptional regulation

**DOI:** 10.1186/s12863-018-0697-5

**Published:** 2019-01-16

**Authors:** Lu Shen, Gang Ma, Ye Shi, Yunfeng Ruan, Xuhan Yang, Xi Wu, Yuyu Xiong, Chunling Wan, Chao Yang, Lei Cai, Likuan Xiong, Xueli Gong, Lin He, Shengying Qin

**Affiliations:** 1 Bio-X Institutes, Key Laboratory for the Genetics of Developmental and Neuropsychiatric Disorders, Ministry of Education, Shanghai Jiao Tong University, 209 Little White House, 1954 Hua Shan Road, Shanghai, 200030 People’s Republic of China; 2 School of Biomedical Engineering, Shanghai Jiao Tong University, Shanghai, 200240 People’s Republic of China; 3 Institute for Nutritional Sciences, Shanghai Institutes for Biological Sciences (SIBS), Chinese Academy of Sciences, Shanghai, 200031 People’s Republic of China; 4 Shanghai Center for Women and Children’s Health, Shanghai, 200062 People’s Republic of China; 5 Center Laboratory, Baoan Maternal and Children Healthcare Hospital, Shenzhen, China; 6 Key Laboratory of Birth Defects Research, Shenzhen, China; 7 Birth Defects Prevention Research and Transformation Team, Shenzhen, China; 8 The Third Affiliated Hospital, Guangzhou Medical University, Guangzhou, 510150 People’s Republic of China

**Keywords:** Brachydactyly type A1, Indian hedgehog, Gli1, E95K

## Abstract

**Background:**

Brachydactyly type A1 (BDA1, OMIM 112500) is a rare inherited malformation characterized primarily by shortness or absence of middle bones of fingers and toes. It is the first recorded disorder of the autosomal dominant Mendelian trait. Indian hedgehog (*IHH*) gene is closely associated with BDA1, which was firstly mapped and identified in Chinese families in 2000. Previous studies have demonstrated that BDA1-related mutant IHH proteins affected interactions with its receptors and impaired IHH signaling. However, how the altered signaling pathway affects downstream transcriptional regulation remains unclear.

**Results:**

Based on the mouse C3H10T1/2 cell model for IHH signaling activation, two recombinant human IHH-N proteins, including a wild type protein (WT, amino acid residues 28–202) and a mutant protein (MT, p.E95k), were analyzed. We identified 347, 47 and 4 Gli1 binding sites in the corresponding WT, MT and control group by chromatin immunoprecipitation and the overlapping of these three sets was poor. The putative cis regulated genes in WT group were enriched in sensory perception and G-protein coupled receptor-signaling pathway. On the other hand, putative cis regulated genes were enriched in *Runx2*-related pathways in MT group. Differentially expressed genes in WT and MT groups indicated that the alteration of mutant IHH signaling involved cell-cell signaling and cellular migration. Cellular assay of migration and proliferation validated that the mutant IHH signaling impaired these two cellular functions.

**Conclusions:**

In this study, we performed integrated genome-wide analyses to characterize differences of IHH/Gli1 downstream regulation between wild type IHH signaling and the E95K mutant signaling. Based on the cell model, our results demonstrated that the E95K mutant signaling altered Gli1-DNA binding pattern, impaired downstream gene expressions, and leaded to weakened cellular proliferation and migration. This study may help to deepen the understanding of pathogenesis of BDA1 and the role of IHH signaling in chondrogenesis.

**Electronic supplementary material:**

The online version of this article (10.1186/s12863-018-0697-5) contains supplementary material, which is available to authorized users.

## Background

The Hedgehog (HH) signaling pathway is a key signaling pathway that regulates a wide range of developmental processes in vertebrate and invertebrate organisms [1–3]. HH proteins in mammals consist of three homologues: Indian hedgehog (IHH), Sonic hedgehog (SHH) and Desert hedgehog (DHH). In mammal developing cartilage, IHH is primarily expressed by chondrocytes. IHH protein interacts with its receptor Patched 1 (PTCH1) or other partners, followed by the transduction of the signaling through Smoothened (SMO) protein. This process ultimately regulates expression of downstream genes by Gli zinc finger domain-containing transcription regulators (Gli1 to − 3) [4, 5]. Transcription regulator Gli1 is a central activator of the HH pathway, while Gli2 and Gli3 serve as activators or as repressors depending on HH signaling level [6–8]. HH/Gli signaling pathway regulates multiple aspects of endochondral bone formation and controls the growth of skeleton by coordinating chondrocyte proliferation and differentiation [9]. Studies of chondrogenesis suggested that there existed a complicated network of various signal interactions [10] involving IHH signaling. Gli-mediated regulation of IHH signaling in vertebrates controls downstream gene expressions during the cartilage development [11]. The significant discoveries of the direct targets of Gli-activator in HH signaling made in last few years [12–16] have provided new insights to our understanding of general mechanism of HH/Gli downstream regulation.

Heterozygous missense mutations in IHH result in brachydactyly type A1 (BDA1; OMIM 112500), a condition characterized by the shortening of digits due to hypoplasia/aplasia of the middle phalanx [17, 18]. In 2009 and 2011, two studies have revealed that the BDA1-related mutant IHH impaired its interaction with PTCH1 and HIP1 and lowered the responses close to the source but increased those at a distance. The final enhanced IHH signaling range might cause reduced bone growth [5, 19]. Recent work has indicated that HH signaling transduced into a gradient of Gli activity that orchestrates patterning of development, while relatively small differences in the ration of Gli forms (Gli activators and repressors) dictated distinct transcriptional outputs [20]. However, how the BDA1-related mutant IHH signaling changes Gli-mediated transcriptional regulation remains unclear.

In this study, we aimed to initiate an effort to characterize the alteration of Gli1-mediated transcriptional regulation caused by E95K mutant signaling. First, C3H10T1/2 cells were induced with wild type (WT) or E95K mutant (MT) IHH protein to activate IHH signaling. After 2 days’ incubation, chromatin immunoprecipitation (ChIP) was conducted and then the DNA products were used to interrogate ChIP-chip analysis for identifying and mapping gli1-binding sites. Then we performed microarray-based gene expression analysis on RNA derived from the induced cells. All the data was integrated by bioinformatics analyses to construct IHH/Gli1 downstream pathway networks. In sum, our results demonstrated significant differences of cartilage-specific Gli1-mediated transcriptional regulation between WT group and MT group. This discovery will help us to unveil the underlying intracellular mechanisms that modulate pathogenesis with BDA1 and to promote the cartilage defect therapy.

## Methods

### IHH protein expression and purification

The pGEX-2 T-based recombinant human IHH-N proteins, including wild type protein (amino acid residues 28–202) and a mutant protein (E95k), were expressed by the BL21 (DE3) strain of *Escherichia coli* (*E. coli*). We obtained the two transformed strains from Bio-X Institutes, Shanghai Jiao Tong University, which had been published earlier [19]. After coated plates, monoclonal colony was selected and incubated in 5 ml LB liquid medium with ampicillin at 37 °C overnight. Then the medium was transferred to 400 ml 2 × YT liquid medium with ampicillin and incubated at 37 °C for 4 h. OD600 reached 0.6 when the strains were induced with 0.2 mM IPTG at 32 °C for 5 h.. Pellets were collected by centrifugation at 8000×g for 10 min at 4 °C and re-suspended in 200 mM NaCl and 20 mM Tris-HCl, pH 7.5 with cocktail protein inhibitors (Sigma-Aldrich), followed by sonication. After centrifugation at 25000×g for 30 min at 4 °C, the supernatant was collected. Then the proteins were purified according to the Bulk and RediPack GST Purification Modules (GE-Healthcare), followed by cleavage of the GST tag using thrombin. The purified IhhN proteins were verified by western blotting with IHH Antibody (SC-1782, Santa Cruz) and filtered by 0.22 μm filter (Millipore).

### IHH activation of C3H10T1/2 cell

The induction was performed following the method that had been described in previous study [19]. C3H10T1/2 cells (obtained from ATCC) was cultured in 12-well plates in a growth medium containing MEM/EBSS, 10% fetal bovine serum (FBS), and 2% penicillin/streptomycin. WT or MT IhhN (E95K) was added into the growth mediums (1x = 750 nM IhhN protein finally) when cells grow to 5 × 10^4^ per well. After 48 h of further incubation at 37 °C (5% CO_2_), the cells were harvested for further analysis. Expression of *Gli1, Ptch1* and *Ihh*, as markers of IHH signaling pathway, were detected by real time PCR. All the induction assays were performed in triplicate.

### ChIP-chip analysis

We used EZ-ChIP Kit (Millipore) to perform chromatin immunoprecipitation. After isolating sheared crosslinked chromatin, each sample (1 × 10^5^cells) was incubated overnight with 5 μg polyclonal Gli1 antibody (AB3444, Millipore) or matched IgG controls. Then 60 μL Protein G Agarose was added to each sample and incubated for 1 h at 4 °C with rotation. After incubation, Protein G Agarose was pelleted by brief centrifugation and washed according to the instruction manual. The pellets were incubated with 100 μL elution buffer (10 μL 20% SDS, 20 μL 1 M NaHCO_3_ and 170 μL sterile, distilled water) at room temperature for 15 min twice and the supernatant was collected. To each sample collection (totally 200 μL), 8 μL 5 M NaCl was added and incubated at 65 °C overnight to reverse the DNA-protein crosslink. On the next day, the samples were sequentially treated with RNAse A and Proteinase K. DNAs were purified using PCR Purification Kit (Qiagen) and amplified using GenomePlex® Whole Genome Amplification Kit (Sigma-Aldrich).

For each ChIP, 2 μg DNA was labeled with Cy5-dUTP for amplified ChIP sample and Cy3-dUTP for input sample and hybridized on MM9 ChIP 3x720K RefSeq Promoter microarray (Roche Nimblegen) according to the manufacturer’s recommendation. Three independent biological sample groups were employed in the analysis and one of the three groups was chosen to conduct further analyses based on its IHH activation level. Arrays were scanned using a Nimblegen MS 200 Microarrary Scanner at 2 μm resolution. Array signals were extracted using NimbleScan Software version 2.6 (Roche Nimblegen) according to the manufacturer’s instruction.

### Microarray-based gene expression analysis

We used TRIZOL™ solution (Invitrogen) to extract the total RNA from cell samples according to the manufacturer’s instruction. RNAs were examined by Agilent 2100 Bioanalyzer to assure its RIN to be more than 7. MicroRNA microarray analyses were performed using 200 ng total RNA on Mouse GE 4x44K v2 Microarray (Agilent Technologies). The amplification, labeling, hybridization and scanning of samples were accomplished by Agilent Technologies (Shanghai, China) using standard protocols. The data were extracted by Feature Extraction version 12.5 (Agilent Technologies) and analyzed by Genespring GX version 12.6 (Agilent Technologies).

### Validation of Results from Microarray-based Analyses by Quantitative Real-time PCR

All qPCR data generated were compliant with current Minimum Information for Publication of Quantitative Real-Time PCR Experiment (MIQE) standards according to Bustin et al. [21].

RNA was reverse transcribed to cDNA using the transcriptor high-fidelity cDNA synthesis kit (Roche), with random hexamers according to the manufacturer’s instructions. We used qPCR with SYBR Green to validate results of microarrays. Primers for ChIP-chip and gene expression microarray were designed by Beacon Designer version 7.9 (PREMIER Biosoft). The PCR reaction was performed using the FastStart Universal SYBR Green Master (Rox) Kit (Roche Diagnostics) on ViiA™ 7 Real-Time PCR System (Applied Biosystems). Each sample in triplicates was assessed in denaturation at 94 °C for 10 min, following by 40 cycles of denaturation at 94 °C for 15 s and annealing at 60 °C for 40 s. Expression levels were assessed in triplicate, normalized to *Gapdh* and *Hprt* control levels. All the sequences of the validation primers are shown in Additional file 1.

### Microarray data process

All the microarray data generated were compliant with current Minimum Information About a Microarray Experiment (MIME) standards according to Brazma et al. [22].

### ChIP-chip data process

Raw data of microarrays were generated in pair format using NimbleScan software version 2.6 (Roche Nimblegen). Peak calling was accomplished by MA2C peak calling algorithm (version 1.0.0, *P* = 1e-5, Robust method, C = 2) [23]. The raw data had been submitted to NCBI Gene Expression Omnibus (GSE74022) [24]. Resulting peak files in bed and wig formats were used as input for Cis-regulatory Element Annotation System [25] to annotate peaks with genome features within Cistrome [26] galaxy environment.

To discover de novo motifs of Gli1, MEME-CHIP [27] analysis were conducted on around 500 bp sequences on surrounding peak centers for peak sets. Cluster analysis of peaks were performed following functional enrichment analysis with Database for Annotation, Visualization and Integrated Discovery (DAVID) [28, 29] and Gene Ontology Analysis [30–32]. We used Reactome platform to conduct pathway analysis of the predicted genes [33].

To identify correlation between Gli1-binding peaks and potential target genes, we annotated each peak to any gene within 25 kb from peaks to transcription start site (TSS) region according to the UCSC browser within the Galaxy environment.

### Gene expression microarray data process

The differentially expressed genes (DEGs) among three groups were identified and clustered using Genespring GX version 12.6 (Agilent Technologies). DEGs were filtered by a criteria of fold change> 2.0. The raw data could be obtained from GEO (GSE74021).

### Integrated analysis of microarray data

We used Ingenuity Pathway Analysis (IPA, Qiagen Corp.) to integrate transcriptional DNA-binding sites and mRNA expression for prediction of interactions between signaling pathways. We used Galaxy (https://usegalaxy.org/) for sequence manipulating, correlation computing and genomic interval operation [34–36]. We used Binding and Expression Target Analysis to combine ChIP-chip with differential gene expression data to infer direct target genes [37]. We used VennDiagram in R to draw venn diagrams [38].

### Cell migration and proliferation test

For cell migration test, C3H10T1/2 cells were seed into 6-well tissue culture plates and grew in MEM/EBSS medium with 10% FBS until they reach ~ 70–80% confluence as a monolayer. We scratched the monolayer with a new 10 μl pipette tip across the well. After scratching, the cells were washed twice with medium and replenished with fresh medium. After 48 h of incubation, the cells were washed twice with 1x PBS and fixed with 3.7% paraformaldehyde. The gap distances were evaluated quantitatively by ImageJ software. Experiment in each group was run in triplicate.

For cell proliferation test, we used MTT Cell Proliferation and Cytotoxicity Assay Kit (Beyotime, China). C3H10T1/2 cells were seed into 96-well tissue culture plate and each well contained ~ 1000 cells within 100 μl MEM/EBSS medium with 10% FBS. After 48 h of culturing with or without IHH-induction, each well was added with 10 μl MTT and the incubation continued for 4 h. Then 100 μl formanzan solution was added into each well. After 4 h of incubation, the absorbance was calculated at 570 nm. Experiment in each group was repeated in triplicate.

## Results

### Activation of IHH signaling pathway in C3H10T1/2 cells induced by IhhN proteins

**Fig. 1:**
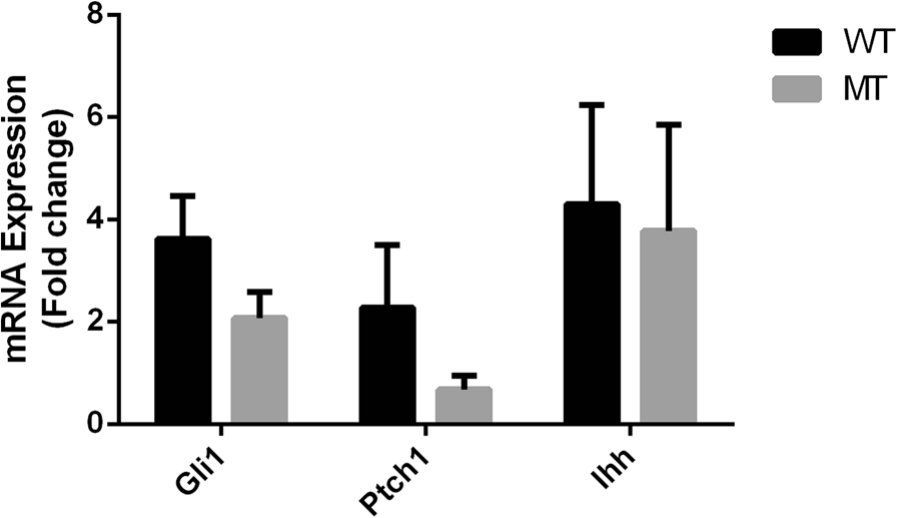
Relative mRNA expression level of *Gli1*, *Ihh* and *Ptch1* after IHH activation. Relative to control group, mRNA expression of *Gli1*, *Ihh* and *Ptch1* in WT and MT group after 2-day incubation with 750 nM IhhN protein

According to previous BDA1 cell model [19], after incubation of C3H10T1/2 cells with 750-nM IhhN protein for 48 h, targets of activated IHH signaling, such as *Gli1*, *Ptch1* and *Ihh*, were examined using quantitative real time PCR in two groups (Fig. 1). *Ihh* and *Gli1* were up-regulated after induction. The expression of *Gli1* was higher in WT group than in MT group (3.63 vs 2.07, *p* = 0.051). These results suggested that E95K mutant IHH weakened the activation of IHH signaling. Interestingly, we observed that *Ptch1*, a 12-transmembrane domain receptor belonging to the Hedgehog (HH) signaling, was up-regulated in WT group but down-regulated in MT group (2.27 vs 0.67, *p* = 0.093). The binding between IHH and PTCH1 contributes to the blocking of inhibitory effects of PTHC1 on the activity of SMO, thus activating the IHH signaling downstream targets [39].

### Genome-wide characterization of Gli1-binding sites reveals the differences of downstream regulation in response to IHH signaling between two groups

**Fig. 2:**
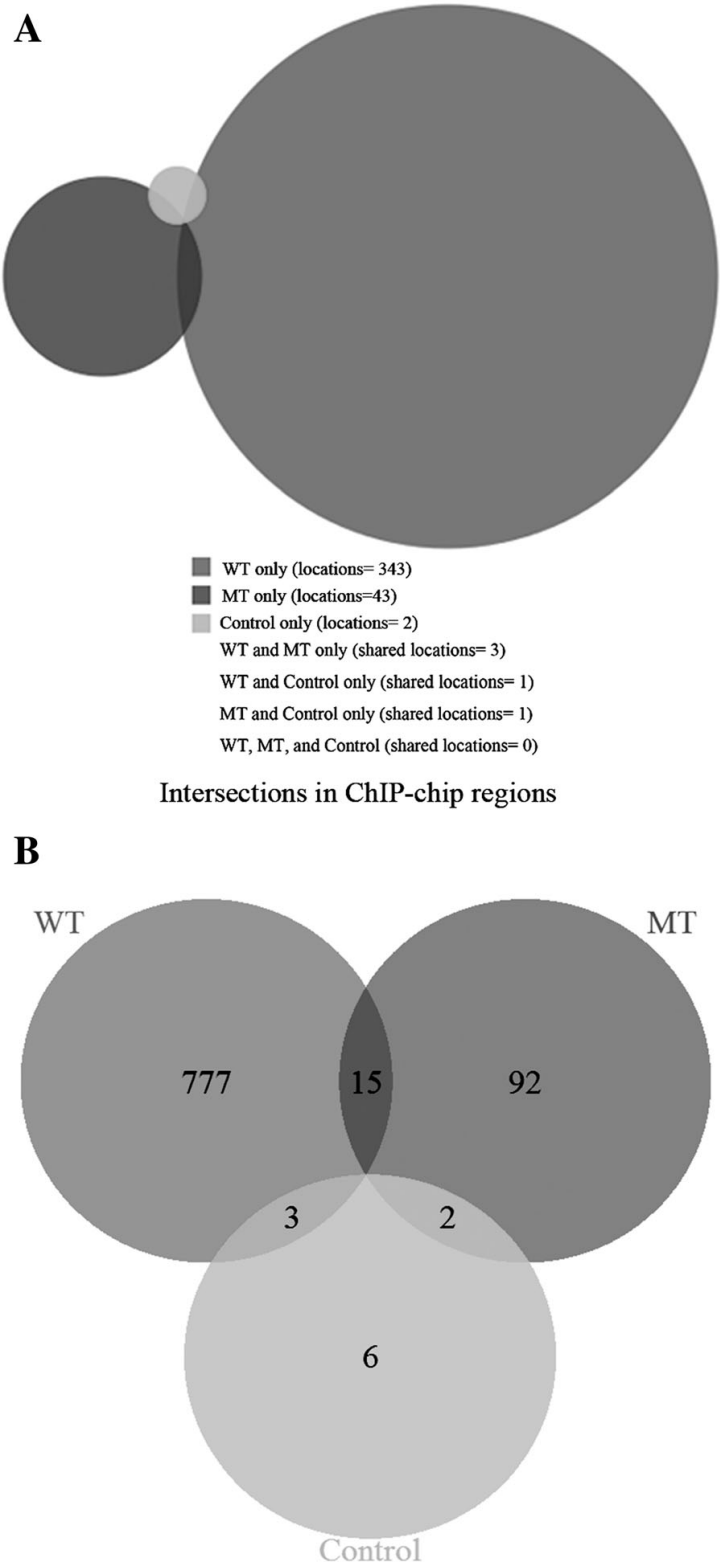
Results from ChIP-chip analysis. Results from ChIP-chip data indicated Gli1-mediated regulatory pattern in WT group had a significant difference from that in MT group. **a** Venn diagram of Gli1 binding sites (**b**) Venn diagram of predicted Gli1 downstream regulatory targets

Based on data from ChIP-chip experiments, we conducted the peak calling using MA2C algorithm [23] and identified 347 peaks in WT group, 47 peaks in MT group and 4 peaks in control group (peak selection threshold = *p*-value <10e-5) (Additional file 2). Among WT, MT and control group there was few overlapping of Gli1 binding sites (Fig. 2a), which indicated that the alteration of IHH signaling had a great impact on Gli1-mediated downstream regulation. We performed de novo motifs searching on the sequences derived from ChIP-chip peaks. However, no significant Gli1 binding consensus sequences were found in any dataset. To verify the ChIP-chip results, a subset of Gli1-binding sequences was validated by real time PCR (Additional file 1).

### Characterization of cis-regulation targets mediated by Gli1 in response to IHH signaling

Based on the NCBI RefSeq genes database, we annotated sequences within 25 k bp of Gli1 binding sites to characterize potential Gli1-regulated targets and identified 795 genes in WT group, 109 genes in MT group and 11 genes in control group using the methods described in earlier study [40] (Additional file 3). Gli1 appeared to regulate more targets in WT group than in MT group and there existed a very small number of overlapping candidate genes in two groups (Fig. 2b). The result of gene enrichment analysis showed that there were significant enrichments of cell-to-cell signaling and interaction related biological processes among candidate genes in WT group, while there were no significant enrichments found in MT and control group (Table 1).

**Table 1:** Gene Enrichment of targets in WT group

GO biological process complete	*Mus musculus* - REFLIST (22262)	Gene sets in WT	Expected	Over/under	Fold Enrichment	Raw *P*-value	FDR
Sensory perception of smell (GO:0007608)	1089	82	34.05	+	2.41	1.33E-12	2.05E-08
Sensory perception of chemical stimulus (GO:0007606)	1377	92	43.05	+	2.14	2.91E-11	1.49E-07
G-protein coupled receptor signaling pathway (GO:0007186)	1793	111	56.06	+	1.98	1.73E-11	1.33E-07
Sensory perception (GO:0007600)	1757	102	54.93	+	1.86	4.68E-09	1.20E-05
Nervous system process (GO:0050877)	2120	115	66.28	+	1.74	1.72E-08	3.77E-05
System process (GO:0003008)	2592	133	81.04	+	1.64	2.42E-08	4.14E-05
Signaling (GO:0023052)	4966	227	155.26	+	1.46	7.79E-10	3.00E-06
Cell communication (GO:0007154)	5084	231	158.95	+	1.45	1.04E-09	3.20E-06
Signal transduction (GO:0007165)	4629	206	144.72	+	1.42	6.62E-08	1.02E-04
Multicellular organismal process (GO:0032501)	7058	293	220.66	+	1.33	1.77E-08	3.41E-05
Cellular response to stimulus (GO:0051716)	6089	242	190.37	+	1.27	2.37E-05	2.80E-02
Cellular process (GO:0009987)	13,831	486	432.41	+	1.12	2.96E-05	3.25E-02
Biological_process (GO:0008150)	20,540	671	642.16	+	1.04	1.17E-05	1.50E-02
Unclassified (UNCLASSIFIED)	1722	25	53.84	–	0.46	1.17E-05	1.64E-02

Then we performed microarray-based analysis for mRNA expression profile. Differential genes expression (DEGs, fold change > 2 compared to control group) was analyzed in WT and MT group (Additional file 4). We identified genes with the largest differences in gene functions between WT group and MT group (Table 2). Expression of seven genes with this signature was validated by real-time PCR (Additional file 5).

**Table 2:** Top 5 Molecular and Cellular Functions

Both Up-regulated in WT and MT group	#Moleculars	Up-regulated in WT and Down-regulated in MT	#Moleculars
Cell Morphology	36	Cell-To-Cell Signaling and Interaction	10
Cellular Development	25	Cell Morphology	5
Cellular Function and Maintenance	22	Cellular Assembly and Organization	5
Cellular Assembly and Organization	18	Cellular Function and Maintance	4
Cellular Movement	11	Cellular Movement	3

To characterize targets of Gli1 cis-regulation, we matched up-regulated genes among DEGs with putative Gli1-targeting genes from ChIP-chip. The result showed that *Cfd* was the only one gene shared in the two datasets while 32 matched genes in WT group and 4 matched genes in MT group were found (Additional file 6).

### E95K mutant signaling impaired cellular migration and proliferation

**Fig. 3:**
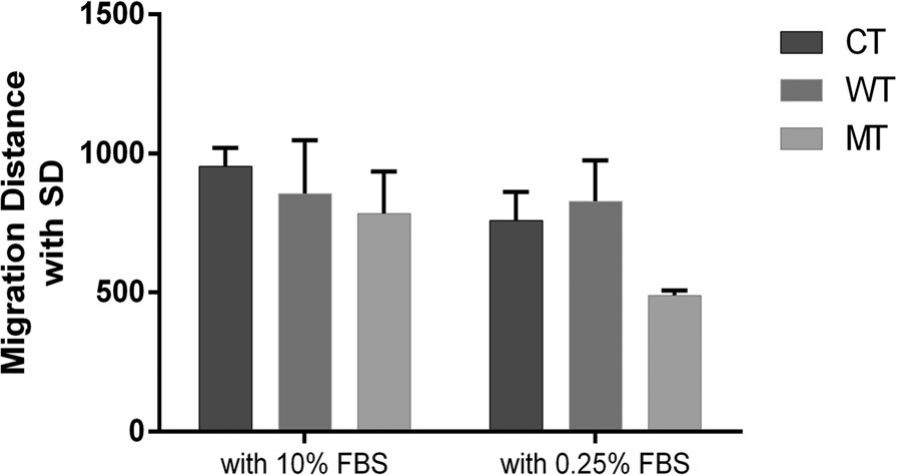
Cell migration assay. Cells in MT group showed lower migration speed than those in WT group (*p* = 0.64 with 10% FBS, *p* = 0.02 with 0.25% FBS)

**Fig. 4:**
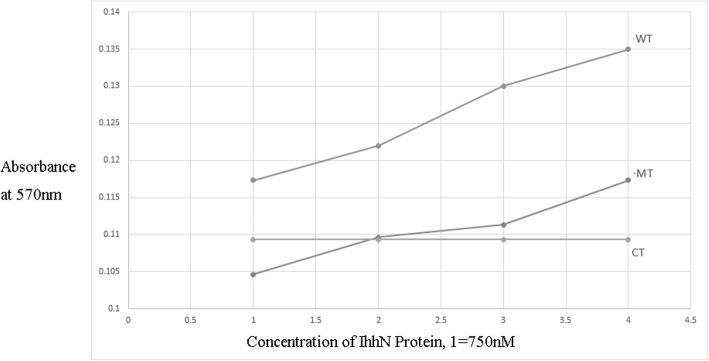
MTT proliferation assay. With increasing concentrations of IhhN protein, the rate of cellular proliferation also increased. Moreover, cells in WT group showed a higher rate than those in MT at the same concentration

According to our previous results, Gli1 targets and DEGs were enriched in cell-to-cell signaling and interaction as well as cellular activities (Tables 1 and 2). *Wnt5a*, an enhancer of cell migration and invasion, was down regulated in MT group. Cell migration and proliferation assay was performed to analyze the impact of altered IHH signaling on cellular activity. We measured the migration speed with high (10%) or low (0.25%) FBS concentration. We calculated the absorbance of MTT at 570 nm after 48 h of cell growth based on a gradient of IhhN protein incubation. Cells in WT group had a higher migration speed (Fig. 3) and proliferation speed (Fig. 4) than those in MT group. The ability of cellular proliferation increased when IhhN concentrations increased (Fig. 4).

### Pathway analysis reveals potential pathogenesis of E95K-induced BDA1

We used Reactome platform to conduct pathway analysis and showed top 25 significant pathways (Additional file 7 and Additional file 8) [33]. Predicted Gli1-target genes were enriched in *Runx2*-related pathways in MT group (6 of top 25 pathways, *p* < 0.05, Additional file 8) but not in WT group (Additional file 7).

The Gli1 binding position in MT group (chr17: 44,872,717-44,873,366) locates at exon1 or intron1/2 of some *Runx2* transcript variants. *Runx2* is a transcription factors and encodes a nuclear protein with a Runt DNA-binding domain. This protein is essential for osteoblastic differentiation and skeletal morphogenesis and acts as a scaffold for nucleic acids and regulatory factors involved in skeletal gene expression. Both loss-of-function and gain-of-function of *Runx2* would result in brachydactyly. It was reported that intragenic duplication encompassing exons 3–5 or 3–6 of *Runx2* gene was associated with brachydactyly phenotype [41, 42]. Real-time PCR results showed that *Runx2* had a lower expression level in both WT and WT groups compared to the control group. However, *Runx2* expression in MT appeared to be higher than in WT group (Additional file 9). And gene expression profile showed that *Mmp13*, the downstream target of *Runx2*, had a lower expression in WT than in MT group (Additional file 4). These results indicated that the E95K mutant IHH signaling had an impaired inhibition effect on *Runx2* expression and its downstream regulation, which was likely to cause brachydactyly.

## Discussion

In this study, we reported that the E95K mutant IHH signaling led to a significant alteration of Gli1-DNA binding pattern and weakened cellular function of migration and proliferation. Our results suggested that E95K mutation impaired the potency of IHH signaling at transcriptional regulation. This discovery was consistent with previous report that had shown that E95K mutant IHH impaired interactions with its partners in mouse model with digit abnormalities [5]. Our findings would be helpful to explain the mechanism of IHH/Gli1 downstream regulation altered by IHH p.E95K mutation.

In earlier studies, three mutant IHH protein, including E95K, D100E and E131K, were found in Chinese BDA1 families [17]. Further analysis of the processing and intracellular maintenance of BDA1 mutant IhhN proteins suggested that E95K mutant IHH protein had a similar stability as wild type IHH protein [19]. In this study, we minimized the possibility of differences caused by degradation of IHH proteins during induction. In addition, we focused on Gli1, a central positive transcriptional regulator without repressor forms among Gli genes [43], in order to simplify the analyses in our study. We also selected 750 nM concentration of IHH protein for signaling induction since it had been reported that activation of the signaling was most sensitive at this concentration [44].

Approaches for genome-scale transcription factor binding screening have been well developed in these years. The approaches had been successfully applied in researches on yeast [45], embryonic stem cells [46] and Drosophila embryos [47]. After that, integrative analysis by combining DNA-binding sites and gene expression data advanced the precise identification of transcription regulator target genes [12, 40, 48]. In this study, we managed to combine ChIP-chip data and gene expression data to propose a Gli1-mediated model of BDA1. There were some existing bioinformatics tools for integrative analysis such as BETA (Binding and Expression Target Analysis) for ChIP and mRNA expression data [37]. However, these methods required large datasets, such as experimental replications, to obtain credible results. Therefore, they were not applicable for a small number of specific samples in this study. It had been reported that integrative analyses like combining ChIP data with MCA analysis would greatly improve the optimal predictive rate of regulator target genes [12].

In this study, we failed to find Gli1 consensus binding sequence GACCACCCA from predicted peaks of ChIP-chip. This may indicated that Gli1 combined with co-transcriptors functioning as regulator in IHH/Gli1 signaling. This assumption was supported by a few reports [12, 49]. Our results suggested that transduction of E95K mutant IHH signaling altered Gli1’s interaction with transcriptional co-regulators and makes Gli1-DNA binding abnormal. Several studies had reported that graded SHH signaling resulted in distinct transcriptional regulation [50, 51]. However, there were few studies reported on how the alteration of IHH signaling affected its downstream transcriptional outputs. In this study, we demonstrated that mutant IHH signaling would lead to a distinct transcriptional pattern compared to wild type IHH signaling (Fig. 2).

We found that *Runx2*, a gene associated with brachydactyly, was differentially expressed between WT and MT group. Based on the evaluation of functional annotations of top 25 pathways from pathway analysis, *Runx2*-related pathway was shown to be one of the most relevant candidates to explain the variation in E95K-induced BDA1 and IHH downstream regulation. More functional analyses are to be conducted to confirm our discovery. Our future research plan will involve two experimental *Runx2*-target drugs, 3-Sulfinoalanine and O-Benzylsulfonyl-Serine, in order to rescue experiment of BDA1 cell model..

## Conclusions

Together, by integrative analyses of Gli1-binding sites scanning, mRNA expression profiling and cellular assays, this study revealed that the E95K mutant IHH signaling significantly changed the potency and effect of its Gli1-mediated downstream transcriptional regulation and identified potential novel targets of IHH/Gli1 pathway related to chondrogenesis. We proposed explanations of how in vivo the E95K mutation alters Gli1-mediated gene regulation and finally impairs IHH signaling capacity in BDA1 model. Our result would provide helpful information to understand the role of IHH signaling in chondrogenesis and pathogenesis in BDA1.

## Additional files

Additional file 1: ChIP Primers. Primer sequences used for ChIP validation. (XLSX 11 kb)

Additional file 2: Peak Calling of Gli1-binding sites. ChIP-chip peak calling. (XLS 66 kb)

Additional file 3: Predicted Gli1 target genes. ChIP-chip predicted genes. (XLS 569 kb)

Additional file 4: Gene Expression Profile. Differentially expressed genes between three groups. (XLS 2660 kb)

Additional file 5: Primers for Real Time PCR. Primers for gene expression validation. (XLSX 11 kb)

Additional file 6: Matched Genes. matched genes between ChIP and DEGs. (XLSX 9 kb)

Additional file 7: Pathway Analysis of Predicted Gli1 target genes in WT. Top 25 pathway analysis of predicted Gli1 target genes in WT. (PDF 1618 kb)

Additional file 8: Pathway Analysis of Predicted Gli1 target genes in MT. Top 25 pathway analysis of predicted Gli1 target genes in MT. (PDF 1633 kb)

Additional file 9: Relative expression of Runx2 with IHH concentration increasing. Relative expression of Runx2 with increased IHH concentrations in WT and MT group compared to control group. (TIFF 489 kb)

## Abbreviations

BDA1: Brachydactyly type A1
*E. coli*: *Escherichia coli*
IHH: Indian Hedgehog
IhhN: Indian Hedghog N-terminus protein
MA2C: Model-based Analysis of 2-Color Arrays
MT: Mutant
TSS: Transcription start site
WT: Wild type
