# Supplementary material for: p.E95K mutation in Indian hedgehog causing brachydactyly type A1 impairs IHH/Gli1 downstream transcriptional regulation
Source: BMC Genet. 2019 Jan 16;20:10. doi: 10.1186/s12863-018-0697-5 (PMC6335781; doi:10.1186/s12863-018-0697-5)
Supplement: Supplementary file 7 — Pathway Analysis of Predicted Gli1 target genes in WT. Top 25 pathway analysis of predicted Gli1 target genes in WT. (PDF 1618 kb) [file 12863_2018_697_MOESM7_ESM.pdf]

reactome

Pathway Analysis Report

This report contains the pathway analysis results for the submitted sample ''. Analysis was performed against Reactome version 66 on 22/10/2018 using any resource identifiers for the mapping. The web link to these results is:

https://reactome.org/PathwayBrowser/#/ANALYSIS=MjAxODEwMjIxMjE5MDBfMTMzMzk%3D

Please keep in mind that analysis results are temporarily stored on our server. The storage period depends on usage of the service but is at least 7 days. As a result, please note that this URL is only valid for a limited time period and it might have expired.

Table of Contents

1. Introduction
2. Properties
3. Genome-wide overview
4. Most significant pathways
5. Pathway details
6. Identifiers found
7. Identifiers not found

1. Introduction

Reactome is a curated database of pathways and reactions in human biology. Reactions can be considered as pathway 'steps'. Reactome defines a 'reaction' as any event in biology that changes the state of a biological molecule. Binding, activation, translocation, degradation and classical biochemical events involving a catalyst are all reactions. Information in the database is authored by expert biologists, entered and maintained by Reactome’s team of curators and editorial staff. Reactome content frequently cross-references other resources e.g. NCBI, Ensembl, UniProt, KEGG (Gene and Compound), ChEBI, PubMed and GO. Orthologous reactions inferred from annotation for Homo sapiens are available for 17 non-human species including mouse, rat, chicken, puffer fish, worm, fly, yeast, rice, and Arabidopsis. Pathways are represented by simple diagrams following an SBGN-like format.

Reactome's annotated data describe reactions possible if all annotated proteins and small molecules were present and active simultaneously in a cell. By overlaying an experimental dataset on these annotations, a user can perform a pathway over-representation analysis. By overlaying quantitative expression data or time series, a user can visualize the extent of change in affected pathways and its progression. A binomial test is used to calculate the probability shown for each result, and the p-values are corrected for the multiple testing (Benjamini–Hochberg procedure) that arises from evaluating the submitted list of identifiers against every pathway.

To learn more about our Pathway Analysis, please have a look at our relevant publications:

Fabregat A, Sidiropoulos K, Garapati P, Gillespie M, Hausmann K, Haw R, … D’Eustachio P (2016). The reactome pathway knowledgebase. Nucleic Acids Research, 44(D1), D481–D487. https://doi.org/10.1093/nar/gkv1351.

Fabregat A, Sidiropoulos K, Viteri G, Forner O, Marin-Garcia P, Arnau V, … Hermjakob H (2017). Reactome pathway analysis: a high-performance in-memory approach. BMC Bioinformatics, 18.

2. Properties

- This is an **overrepresentation** analysis: A statistical (hypergeometric distribution) test that determines whether certain Reactome pathways are over-represented (enriched) in the submitted data. It answers the question 'Does my list contain more proteins for pathway X than would be expected by chance?' This test produces a probability score, which is corrected for false discovery rate using the Benjamani-Hochberg method.
- 329 out of 770 identifiers in the sample were found in Reactome, where 898 pathways were hit by at least one of them.
- All non-human identifiers have been converted to their human equivalent.
- This report is filtered to show only results and pathway diagrams for Homo sapiens.
- The unique ID for this analysis (token) is MjAxODEwMjIxMjE5MDBfMTMzMzk%3D. This ID is valid for at least 7 days in Reactome's server. Use it to access Reactome services with your data.

3. Genome-wide overview

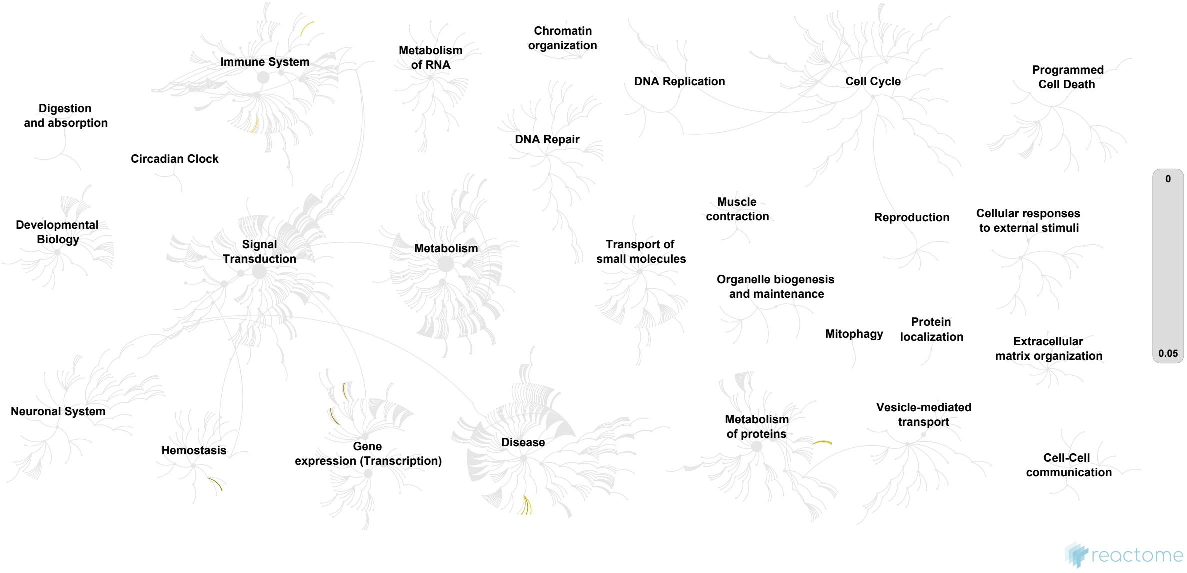

This figure shows a genome-wide overview of the results of your pathway analysis. Reactome pathways are arranged in a hierarchy. The center of each of the circular "bursts" is the root of one top-level pathway, for example "DNA Repair". Each step away from the center represents the next level lower in the pathway hierarchy. The color code denotes over-representation of that pathway in your input dataset. Light grey signifies pathways which are not significantly over-represented.

4. Top 25 pathways

Pathway name	Entities				Reactions	
	found	ratio	p-value	FDR*	found	ratio
TRAF6 mediated IRF7 activation	10 / 43	0.003	2.90e-04	0.279	2 / 11	9.33e-04
Loss of MECP2 binding ability to 5hmC-DNA	2 / 2	1.43e-04	0.006	0.893	1 / 1	8.48e-05
Regulation of IFNA signaling	6 / 28	0.002	0.007	0.893	4 / 5	4.24e-04
Loss of MECP2 binding ability to the NCoR/SMRT complex	3 / 8	5.72e-04	0.012	0.893	1 / 1	8.48e-05
Transport of gamma-carboxylated protein precursors from the endoplasmic reticulum to the Golgi apparatus	3 / 9	6.44e-04	0.017	0.893	3 / 9	7.63e-04
Removal of aminoterminal propeptides from gamma-carboxylated proteins	3 / 10	7.15e-04	0.022	0.893	3 / 9	7.63e-04
Loss of MECP2 binding ability to 5mC-DNA	2 / 5	3.58e-04	0.035	0.893	2 / 2	1.70e-04
TP53 Regulates Transcription of Genes Involved in G2 Cell Cycle Arrest	4 / 21	0.002	0.037	0.893	7 / 11	9.33e-04
MECP2 regulates transcription of genes involved in GABA signaling	2 / 6	4.29e-04	0.049	0.893	4 / 4	3.39e-04
Extrinsic Pathway of Fibrin Clot Formation	2 / 6	4.29e-04	0.049	0.893	7 / 8	6.79e-04
Acrosome Reaction	1 / 1	7.15e-05	0.057	0.893	2 / 2	1.70e-04
Gamma-carboxylation of protein precursors	3 / 15	0.001	0.06	0.893	3 / 9	7.63e-04
Regulation of gene expression by Hypoxia-inducible Factor	3 / 15	0.001	0.06	0.893	2 / 7	5.94e-04
DDX58/IFIH1-mediated induction of interferon-alpha/beta	10 / 96	0.007	0.061	0.893	2 / 46	0.004
PTK6 Expression	2 / 7	5.01e-04	0.064	0.893	3 / 3	2.54e-04
PTK6 Down-Regulation	2 / 7	5.01e-04	0.064	0.893	3 / 3	2.54e-04
Loss of phosphorylation of MECP2 at T308	2 / 7	5.01e-04	0.064	0.893	1 / 1	8.48e-05
Loss of function of MECP2 in Rett syndrome	3 / 16	0.001	0.069	0.893	5 / 5	4.24e-04
Pervasive developmental disorders	3 / 16	0.001	0.069	0.893	5 / 5	4.24e-04
Gamma-carboxylation, transport, and amino-terminal cleavage of proteins	3 / 16	0.001	0.069	0.893	9 / 27	0.002
TYSND1 cleaves peroxisomal proteins	2 / 8	5.72e-04	0.081	0.893	7 / 7	5.94e-04

Pathway name	Entities				Reactions	
	found	ratio	p-value	FDR*	found	ratio
Regulation of MECP2 expression and activity	5 / 39	0.003	0.082	0.893	12 / 14	0.001
TP53 Regulates Transcription of Cell Cycle Genes	7 / 65	0.005	0.092	0.893	11 / 42	0.004
TWIK-related spinal cord K+ channel (TRESK)	1 / 2	1.43e-04	0.111	0.893	1 / 1	8.48e-05
MECP2 regulates transcription factors	2 / 10	7.15e-04	0.118	0.893	8 / 8	6.79e-04

* False Discovery Rate

5. Pathway details

1. TRAF6 mediated IRF7 activation (R-HSA-933541)

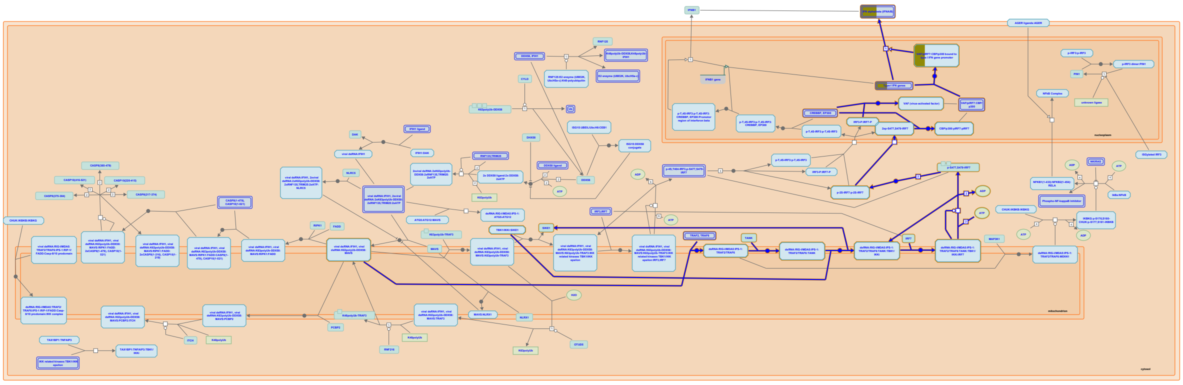

Cellular compartments: mitochondrial outer membrane.

TRAF6 is crucial for both RIG-I- and MDA5-mediated antiviral responses. The absence of TRAF6 resulted in enhanced viral replication and a significant reduction in the production of type I IFNs and IL6 after infection with RNA virus. Activation of NF-kB and IRF7, but not that of IRF3, was significantly impaired during RIG-like helicases (RLHs) signaling in the absence of TRAF6. TRAF6-induced activation of IRF is likely to be specific for IRF7, while TRAF3 is thought to activate both IRF3 and IRF7. These results strongly suggest that the TRAF6- and TRAF3-dependent pathways are likely to bifurcate at IPS-1, but to converge later at IRF7 in order to co-operatively induce sufficient production of type I IFNs during RLH signaling.

References

Konno H, Yamamoto T, Yamazaki K, Gohda J, Akiyama T, Semba K, ... Inoue J (2009). TRAF6 establishes innate immune responses by activating NF-kappaB and IRF7 upon sensing cytosolic viral RNA and DNA. PLoS One, 4, e5674.

Edit history

Date	Action	Author
2010-08-02	Edited	Garapati P V
2010-08-02	Authored	Garapati P V
2010-08-16	Created	Garapati P V
2010-10-30	Reviewed	Akira S, Kawai T
2018-08-23	Modified	Schmidt EE

Elements found in this pathway

Input	UniProt Id	Input	UniProt Id	Input	UniProt Id
Ifna11	P01570	Ifna4	P01569, P05014	Ifna5	P01569, P05013, P01563
Ifna6	P05013	Ifna7	P01567, P05013		

Input	Ensembl Id	Input	Ensembl Id	Input	Ensembl Id
Ifna4	ENSG00000236637	Ifna5	ENSG00000147873	Ifna6	ENSG00000120235

Input	Ensembl Id	Input	Ensembl Id	Input	Ensembl Id
Ifna7	ENSG00000214042				

2. Loss of MECP2 binding ability to 5hmC-DNA (R-HSA-9022534)

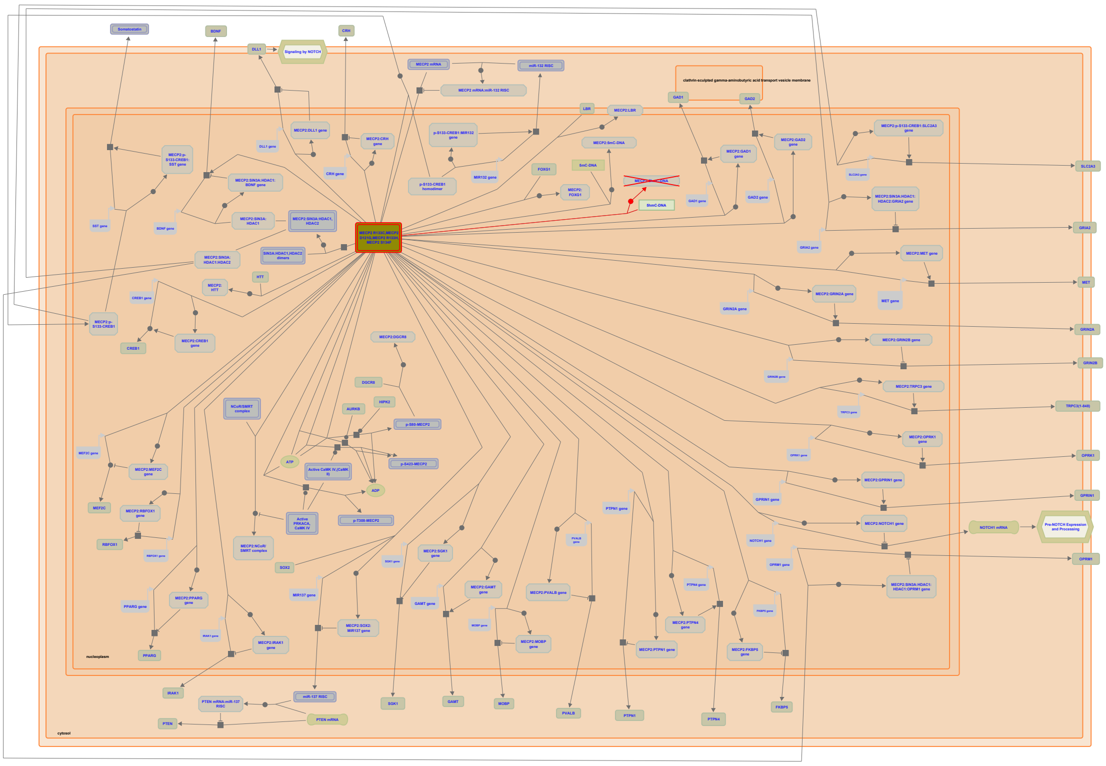

Cellular compartments: nucleoplasm.

Diseases: Rett syndrome.

Missense mutations in the methyl-CpG binding domain (MBD) of MECP2, spanning amino acids 90 to 162, negatively affect the binding ability of MECP2 to hydroxymethylated DNA (Mellen et al. 2012).

References

Mellén M, Ayata P, Dewell S, Kriaucionis S & Heintz N (2012). MeCP2 binds to 5hmC enriched within active genes and accessible chromatin in the nervous system. Cell, 151, 1417-30.

Edit history

Date	Action	Author
2017-09-25	Created	Orlic-Milacic M
2017-10-03	Authored	Orlic-Milacic M
2018-08-07	Reviewed	Christodoulou J, Krishnaraj R
2018-08-08	Modified	Orlic-Milacic M
2018-08-08	Edited	Orlic-Milacic M

Elements found in this pathway

Input	UniProt Id	Input	UniProt Id	Input	UniProt Id
Mecp2	P51608-2, P51608-1				

3. Regulation of IFNA signaling (R-HSA-912694)

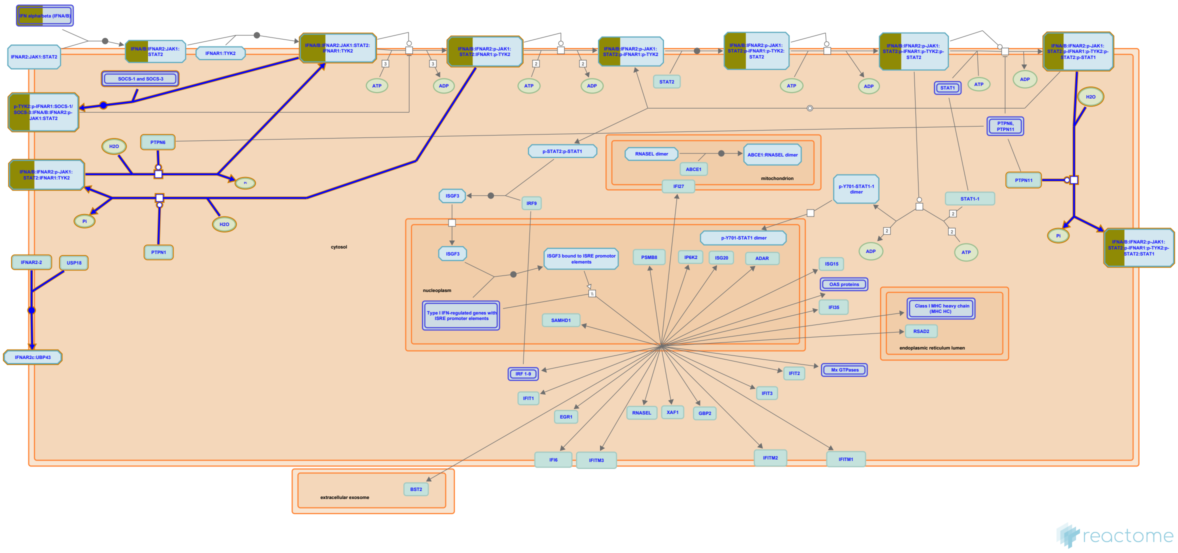

There are several proteins and mechanisms involved in controlling the extent of ligand stimulation of IFNA/B signaling. These mechanisms can effect every step of the IFNA/B cascade. Dephosphorylation of JAK and STAT by SHP protein phosphatases, inhibition of STAT function in the nucleus by protein inhibitors of activated STATs (PIAS) proteins, inhibition of tyrosine kinase activity of JAKs by SOCS as well as inhibition of JAK and IFNAR2 interaction by UBP43 are few of the negative regulation mechanisms in controling type I IFN signaling.

References

Fenner JE, Starr R, Cornish AL, Zhang JG, Metcalf D, Schreiber RD, ... Hertzog PJ (2006). Suppressor of cytokine signaling 1 regulates the immune response to infection by a unique inhibition of type I interferon activity. Nat Immunol, 7, 33-9.

Malakhova OA, Kim KI, Luo JK, Zou W, Kumar KG, Fuchs SY, ... Zhang DE (2006). UBP43 is a novel regulator of interferon signaling independent of its ISG15 isopeptidase activity. EMBO J, 25, 2358-67.

Hilton DJ (1999). Negative regulators of cytokine signal transduction. Cell Mol Life Sci, 55, 1568-77.

Edit history

Date	Action	Author
2010-07-07	Edited	Garapati P V
2010-07-07	Authored	Garapati P V
2010-07-12	Created	Garapati P V
2010-08-17	Reviewed	Abdul-Sater AA, Schindler C
2018-08-30	Modified	Croft D

Elements found in this pathway

Input	UniProt Id	Input	UniProt Id	Input	UniProt Id
Ifna11	P01570	Ifna4	P01569, P05014	Ifna5	P01569, P05013, P01563
Ifna6	P05013	Ifna7	P01567, P05013		

4. Loss of MECP2 binding ability to the NCoR/SMRT complex (R-HSA-9022537)

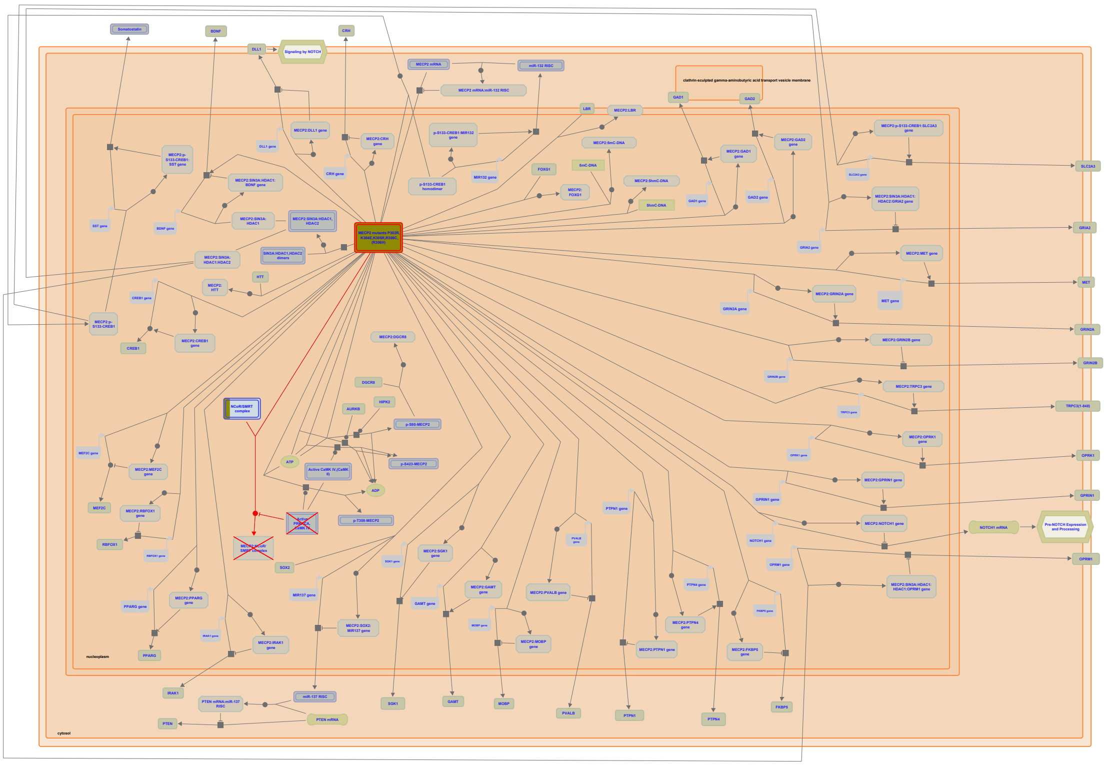

Cellular compartments: nucleoplasm.

Diseases: Rett syndrome.

Missense mutations in the transcriptional repression domain of methyl-CpG-binding protein 2 (MECP2) can negatively affect binding of MECP2 to the nuclear receptor co-repressor (NCoR/SMRT) complex (Lyst et al. 2013, Ebert et al. 2013).

References

Lyst MJ, Ekiert R, Ebert DH, Merusi C, Nowak J, Selfridge J, ... Bird A (2013). Rett syndrome mutations abolish the interaction of MeCP2 with the NCoR/SMRT co-repressor. Nat. Neurosci., 16, 898-902.

Ebert DH, Gabel HW, Robinson ND, Kastan NR, Hu LS, Cohen S, ... Greenberg ME (2013). Activity-dependent phosphorylation of MeCP2 threonine 308 regulates interaction with NCoR. Nature, 499, 341-5.

Edit history

Date	Action	Author
2017-09-25	Created	Orlic-Milacic M
2017-10-03	Authored	Orlic-Milacic M
2018-08-07	Reviewed	Christodoulou J, Krishnaraj R
2018-08-08	Modified	Orlic-Milacic M
2018-08-08	Edited	Orlic-Milacic M

Elements found in this pathway

Input	UniProt Id	Input	UniProt Id	Input	UniProt Id
Mecp2	P51608-2, P51608-1	Ncor2	Q9Y618		

5. Transport of gamma-carboxylated protein precursors from the endoplasmic reticulum to the Golgi apparatus (R-HSA-159763)

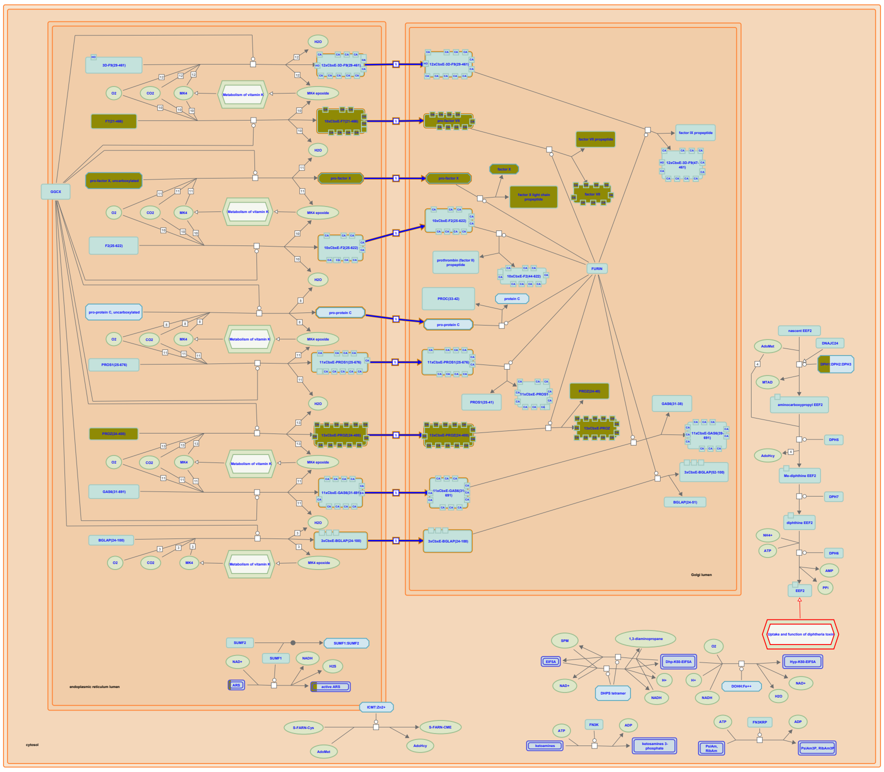

Cellular compartments: COPII-coated ER to Golgi transport vesicle.

Gamma-carboxylated proteins are moved by anterograde transport from the endoplasmic reticulum to the Golgi apparatus (Kirchhausen 2000).

References

Kirchhausen Tomas (2000). Three ways to make a vesicle. Nat Rev Mol Cell Biol, 1, 187-98.

Edit history

Date	Action	Author
2005-03-17	Authored	D'Eustachio P
2005-03-17	Created	D'Eustachio P
2018-08-21	Edited	D'Eustachio P
2018-08-30	Modified	Croft D

Elements found in this pathway

Input	UniProt Id	Input	UniProt Id	Input	UniProt Id
F10	P00742	F7	P08709	Proz	P22891

6. Removal of aminoterminal propeptides from gamma-carboxylated proteins (R-HSA-159782)

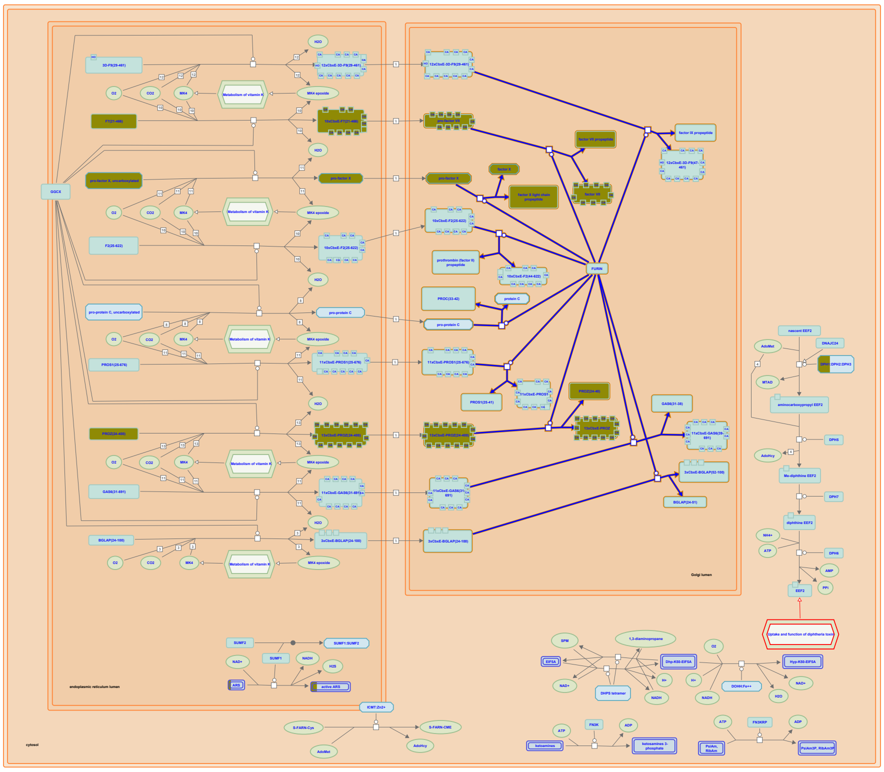

Cellular compartments: Golgi lumen.

Furin is an endopeptidase localized to the Golgi membrane that cleaves many proteins on the carboxyterminal side of the sequence motif Arg-[any residue]-(Lys or Arg)-Arg (Jones et al. 1995; Leduc et al. 1992). In the case of gamma-carboxylated proteins, if this cleavage does not occur, the proteins are still secreted but do not function properly (Bristol et al. 1993; Lind et al. 1997; Wasley et al. 1993). The aminoterminal fragments, "propeptides", generated in this reaction have no known function; the carboxylated, cleaved proteins are delivered to the cell membrane or secreted from the cell.

References

Leduc R, Molloy S S, Thorne BA & Thomas G (1992). Activation of human furin precursor processing endoprotease occurs by an intramolecular autoproteolytic cleavage. J Biol Chem, 267, 14304-8.

Jones BG, Thomas L, Molloy S S, Thulin CD, Fry MD, Walsh KA & Thomas G (1995). Intracellular trafficking of furin is modulated by the phosphorylation state of a casein kinase II site in its cytoplasmic tail. EMBO J, 14, 5869-83.

Lind B, Johnsen AH & Thorsen S (1997). Naturally occurring Arg(-1) to His mutation in human protein C leads to aberrant propeptide processing and secretion of dysfunctional protein C. Blood, 89, 2807-16.

Bristol JA, Furie BC & Furie B (1993). Propeptide processing during factor IX biosynthesis. Effect of point mutations adjacent to the propeptide cleavage site. J Biol Chem, 268, 7577-84.

Wasley LC, Rehemtulla A, Bristol JA & Kaufman RJ (1993). PACE/furin can process the vitamin K-dependent pro-factor IX precursor within the secretory pathway. J. Biol. Chem., 268, 8458-65.

Edit history

Date	Action	Author
2005-03-17	Authored	D'Eustachio P
2005-03-17	Created	D'Eustachio P
2018-08-21	Edited	D'Eustachio P
2018-08-30	Modified	Croft D

Elements found in this pathway

Input	UniProt Id	Input	UniProt Id	Input	UniProt Id
F10	P00742	F7	P08709	Proz	P22891

7. Loss of MECP2 binding ability to 5mC-DNA (R-HSA-9022538)

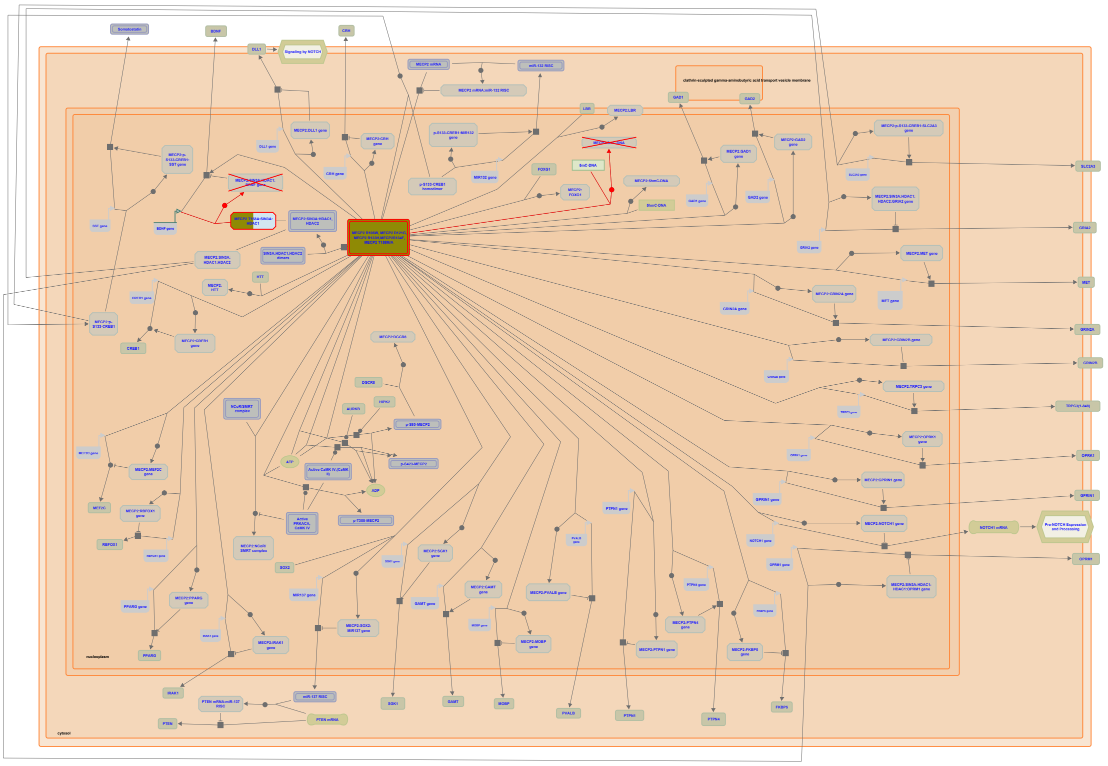

Cellular compartments: nucleoplasm.

Diseases: Rett syndrome.

Missense mutations in the methyl-CpG binding domain (MBD) of methyl-CpG-binding protein 2 (MECP2), spanning amino acids 90 to 162, negatively affect the binding ability of MECP2 to methylated DNA (Ghosh et al. 2008, Ho et al. 2008, Goffin et al. 2012, Mellen et al. 2012).

References

Ho KL, McNae IW, Schmiedeberg L, Klose RJ, Bird AP & Walkinshaw MD (2008). MeCP2 binding to DNA depends upon hydration at methyl-CpG. Mol. Cell, 29, 525-31.

Goffin D, Allen M, Zhang L, Amorim M, Wang IT, Reyes AR, ... Zhou Z (2011). Rett syndrome mutation MeCP2 T158A disrupts DNA binding, protein stability and ERP responses. Nat. Neurosci., 15 , 274-83.

Mellén M, Ayata P, Dewell S, Kriaucionis S & Heintz N (2012). MeCP2 binds to 5hmC enriched within active genes and accessible chromatin in the nervous system. Cell, 151, 1417-30.

Ghosh RP, Horowitz-Scherer RA, Nikitina T, Gierasch LM & Woodcock CL (2008). Rett syndrome-causing mutations in human MeCP2 result in diverse structural changes that impact folding and DNA interactions. J. Biol. Chem., 283, 20523-34.

Edit history

Date	Action	Author
2017-09-25	Created	Orlic-Milacic M
2017-10-03	Authored	Orlic-Milacic M

Date	Action	Author
2018-08-07	Reviewed	Christodoulou J, Krishnaraj R
2018-08-08	Modified	Orlic-Milacic M
2018-08-08	Edited	Orlic-Milacic M

Elements found in this pathway

Input	UniProt Id	Input	UniProt Id	Input	UniProt Id
Mecp2	P51608-2, P51608-1				

8. TP53 Regulates Transcription of Genes Involved in G2 Cell Cycle Arrest (R-HSA-6804114)

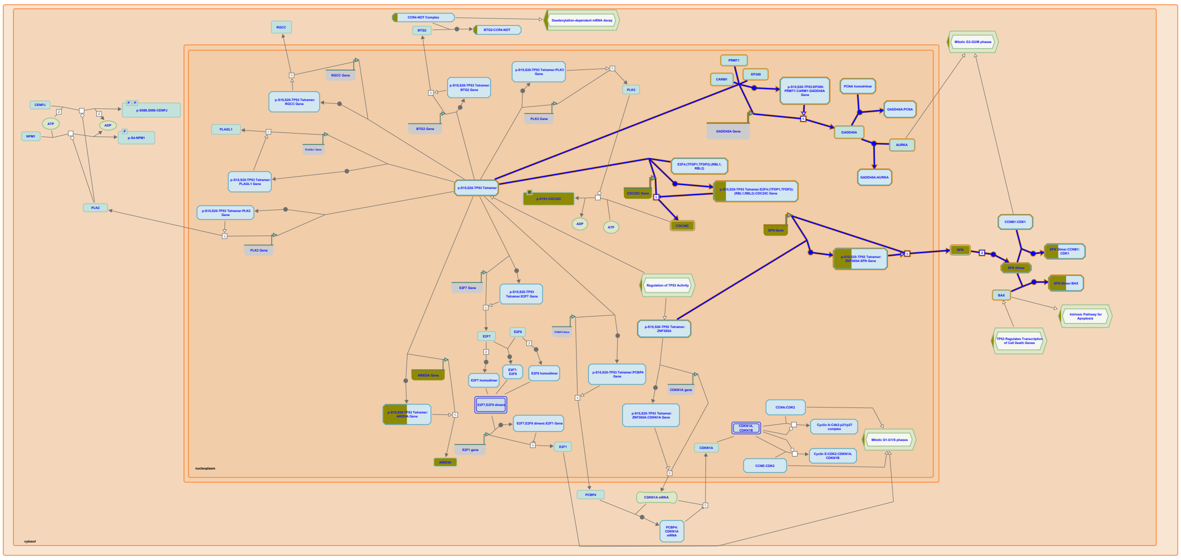

TP53 contributes to the establishment of G2 arrest by inducing transcription of GADD45A and SFN, and by inhibiting transcription of CDC25C. TP53 induces GADD45A transcription in cooperation with chromatin modifying enzymes EP300, PRMT1 and CARM1 (An et al. 2004). GADD45A binds Aurora kinase A (AURKA), inhibiting its catalytic activity and preventing AURKA-mediated G2/M transition (Shao et al. 2006, Sanchez et al. 2010). GADD45A also forms a complex with PCNA. PCNA is involved in both normal and repair DNA synthesis. The effect of GADD45 interaction with PCNA, if any, on S phase progression, G2 arrest and DNA repair is not known (Smith et al. 1994, Hall et al. 1995, Sanchez et al. 2010, Kim et al. 2013). SFN (14-3-3-sigma) is induced by TP53 (Hermeking et al. 1997) and contributes to G2 arrest by binding to the complex of CDK1 and CCNB1 (cyclin B1) and preventing its translocation to the nucleus. Phosphorylation of a number of nuclear proteins by the complex of CDK1 and CCNB1 is needed for G2/M transition (Chan et al. 1999). While promoting G2 arrest, SFN can simultaneously inhibit apoptosis by binding to BAX and preventing its translocation to mitochondria, a step involved in cytochrome C release (Samuel et al. 2001). TP53 binds the promoter of the CDC25C gene in cooperation with the transcriptional repressor E2F4 and represses CDC25C transcription, thus maintaining G2 arrest (St Clair et al. 2004, Benson et al. 2014). The zinc finger transcription factor ZNF385A (HZF) is a direct transcriptional target of TP53 that can form a complex with TP53 and facilitate TP53-mediated induction of SFN transcription (Das et al. 2007).

References

Shao S, Wang Y, Jin S, Song Y, Wang X, Fan W, ... Zhan Q (2006). Gadd45a interacts with aurora-A and inhibits its kinase activity. J. Biol. Chem., 281, 28943-50.

An W, Kim J & Roeder RG (2004). Ordered cooperative functions of PRMT1, p300, and CARM1 in transcriptional activation by p53. Cell, 117, 735-48.

Sánchez R, Pantoja-Uceda D, Prieto J, Diercks T, Marcaida MJ, Montoya G, ... Blanco FJ (2010). Solution structure of human growth arrest and DNA damage 45alpha (Gadd45alpha) and its interactions with proliferating cell nuclear antigen (PCNA) and Aurora A kinase. J. Biol. Chem., 285, 22196-201.

Kim HL, Kim SU & Seo YR (2013). A novel role for Gadd45 in base excision repair: modulation of APE1 activity by the direct interaction of Gadd45 with PCNA. Biochem. Biophys. Res. Commun., 434, 185-90.

Hall PA, Kearsey JM, Coates PJ, Norman DG, Warbrick E & Cox LS (1995). Characterisation of the interaction between PCNA and Gadd45. Oncogene, 10, 2427-33.

Edit history

Date	Action	Author
2015-10-08	Created	Orlic-Milacic M
2015-10-14	Edited	Orlic-Milacic M
2015-10-14	Authored	Orlic-Milacic M
2016-02-04	Reviewed	Zaccara S, Inga A
2018-08-30	Modified	Croft D

Elements found in this pathway

Input	UniProt Id	Input	UniProt Id	Input	UniProt Id
Cdc25c	P30307	Sfn	P31947		

Input	Ensembl Id	Input	Ensembl Id	Input	Ensembl Id
Cdc25c	ENSG00000158402	Sfn	ENSG00000175793		

9. MECP2 regulates transcription of genes involved in GABA signaling (R-HSA-9022927)

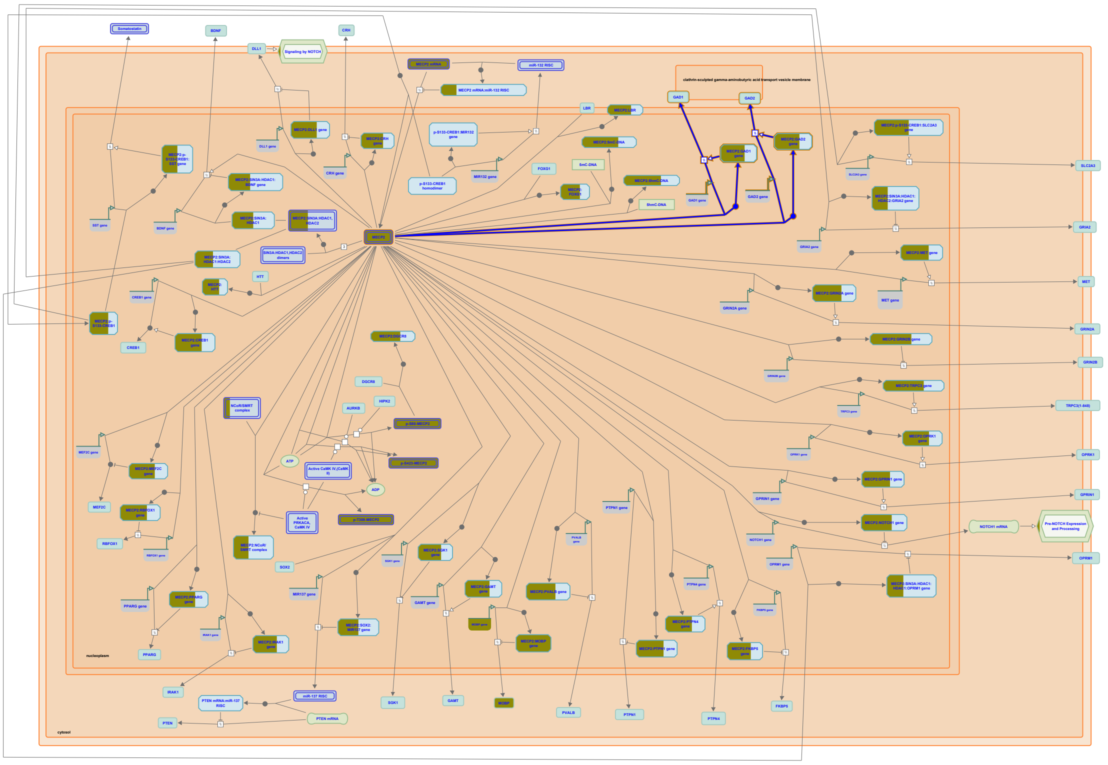

MECP2 regulates expression of several genes involved in GABA (gamma-aminobutyric acid) signaling. Transcription of GAD1 (GAD67) and GAD2 (GAD65) genes is directly positively regulated by MECP2. GAD1 and GAD2 are components of the glutamic acid decarboxylase complex involved in production of the neurotransmitter GABA. Mice lacking Mecp2 from GABA-releasing neurons have decreased GABA levels and exhibit multiple Rett syndrome features (Chao et al. 2010).

Mecp2 deletion in mouse GABAergic parvalbumin-expressing (PV) cells, cortical interneurons playing a key role in visual experience-induced ocular dominance plasticity, does not result in Rett-like phenotype, other than defects in motor coordination and motor learning. While functions of the visual cortex are preserved in mice lacking Mecp2 in GABAergic PV cells, the visual input-induced spiking responses are decreased. Mecp2 loss impairs maturation of membrane functions of cortical GABAergic PV cells. Mecp2 may be needed for PV cell-mediated cortical GABA inhibition. Mecp2-deficient cortical PV cells show reduced mRNA levels of several genes involved in GABA signaling, such as Parvalbumin, Gad2, Calretinin, Gabra1 and Gabra2, as well as reduced levels of Glu3, a glutamate receptor subunit, and Kv3.1, a potassium channel (He et al. 2014).

References

Chao HT, Chen H, Samaco RC, Xue M, Chahrour M, Yoo J, ... Zoghbi HY (2010). Dysfunction in GABA signalling mediates autism-like stereotypies and Rett syndrome phenotypes. Nature, 468, 263-9.

He LJ, Liu N, Cheng TL, Chen XJ, Li YD, Shu YS, ... Zhang XH (2014). Conditional deletion of Mecp2 in parvalbumin-expressing GABAergic cells results in the absence of critical period plasticity. Nat Commun, 5, 5036.

Edit history

Date	Action	Author
2017-09-26	Created	Orlic-Milacic M
2017-10-03	Authored	Orlic-Milacic M
2018-08-07	Reviewed	Christodoulou J, Krishnaraj R
2018-08-08	Modified	Orlic-Milacic M
2018-08-08	Edited	Orlic-Milacic M

Elements found in this pathway

Input	UniProt Id	Input	UniProt Id	Input	UniProt Id
Mecp2	P51608-2, P51608-1				

10. Extrinsic Pathway of Fibrin Clot Formation (R-HSA-140834)

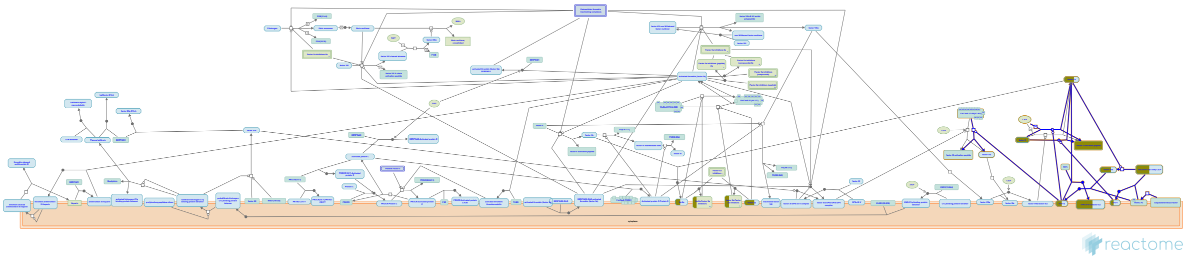

Cellular compartments: extracellular region.

Factor VII, the protease that initiates the normal blood clotting cascade, circulates in the blood in both its proenzyme (factor VII) and its activated (factor VIIa) forms. No clotting occurs, however, because neither form of the protein has any catalytic activity when free in solution. Blood clotting is normally initiated when tissue factor (TF), an intrinsic plasma membrane protein, is exposed to the blood by injury to the wall of a blood vessel. TF is then able to bind factor VIIa from plasma, and possibly also factor VII, to form complexes capable of catalyzing the conversion of factor X, from plasma, into its activated form, factor Xa. Factor Xa catalyzes the conversion of additional factor VII molecules to their activated form, increasing the amount of tissue factor:factor VIIa complex available at the site of injury, accelerating the generation of factor Xa, and allowing the activation of factor IXa as well. This process is self-limiting because as levels of factor Xa increase, tissue factor:factor VIIa complexes become trapped in the form of catalytically inactive heterotetramers with factor Xa and the protein TFPI (tissue pathway factor inhibitor). At this point the intinsic pathway, as an independent source of activated factor X, is thought to become critical for the continuation of clot formation (Broze 1995; Mann et al. 2003).

The nature of the initial tissue factor:factor VII complexes formed is controversial. One model, building on the observation that the complex of factor VII and TF has low but measurable proteolytic activity on factor X, suggests that this complex begins the activation of factor X, and that as factor VIIa accumulates, tissue factor:factor VIIa complexes also form, accelerating the process (Nemerson 1988). A second model, building on the observation that normal plasma contains low levels of activated factor VII constitutively, suggests that complexes with factor VIIa form immediately at the onset of clotting (Rapaport and Rao 1995). The two models are not mutually exclusive, and in any event, the central roles of tissue factor and factor VIIa in generating an initial supply of factors IXa and Xa, and the self-limiting nature of the process due to the action of TFPI, are all well-established.

These events are outlined in the drawing: black arrows connect the substrates (inputs) and products (outputs) of individual reactions, and blue lines connect output activated enzymes to the other reactions that they catalyze.

References

Broze GJ Jr (1995). Tissue factor pathway inhibitor and the revised theory of coagulation. Annu Rev Med, 46, 103-12.

Edit history

Date	Action	Author
2004-08-24	Authored	D'Eustachio P

Date	Action	Author
2004-08-24	Created	D'Eustachio P
2018-08-30	Modified	Croft D

Elements found in this pathway

Input	UniProt Id	Input	UniProt Id	Input	UniProt Id
F10	P00742	F7	P08709		

11. Acrosome Reaction (R-HSA-1300645)

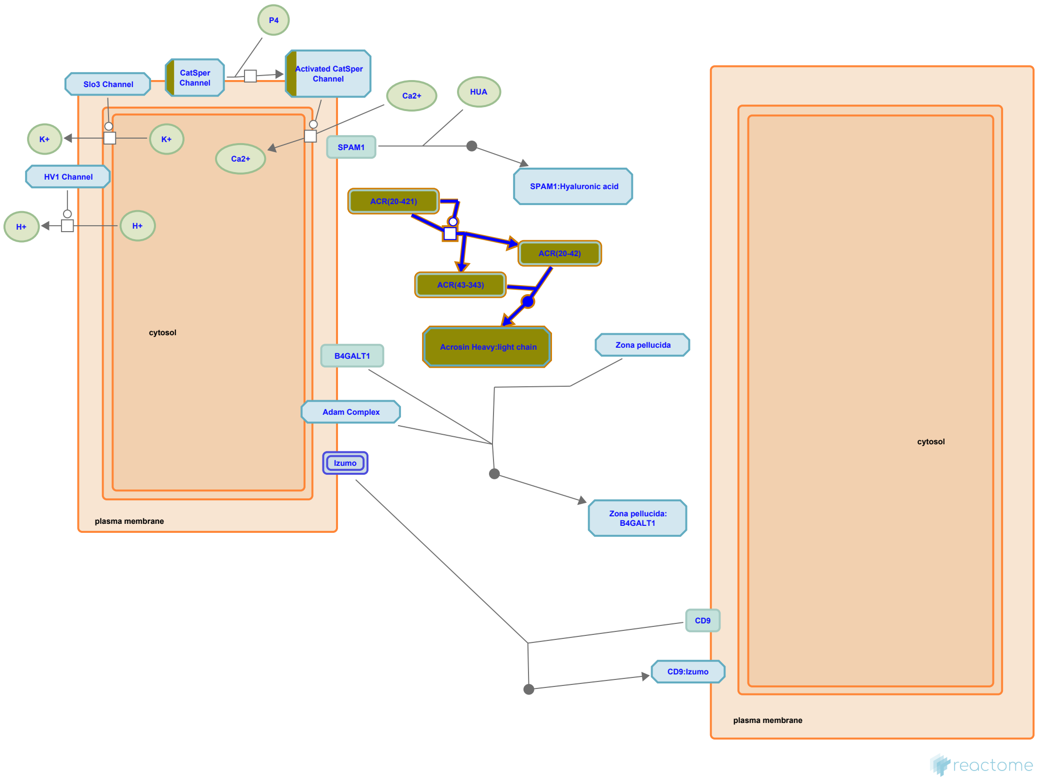

The acrosome reaction is stimulated by zona pellucida binding and subsequent downstream events, including Ca2+ influx. Proacrosin cleavage is the hallmark event of the acrosome reaction.

References

Ikawa M, Inoue N, Benham AM & Okabe M (2010). Fertilization: a sperm's journey to and interaction with the oocyte. J Clin Invest, 120, 984-94.

Edit history

Date	Action	Author
2011-05-24	Created	Gillespie ME
2013-02-13	Authored	Gillespie ME
2013-05-21	Reviewed	Lishko PV
2013-05-23	Edited	Gillespie ME
2018-08-23	Modified	Schmidt EE

Elements found in this pathway

Input	UniProt Id	Input	UniProt Id	Input	UniProt Id
Acr	P10323				

12. Gamma-carboxylation of protein precursors (R-HSA-159740)

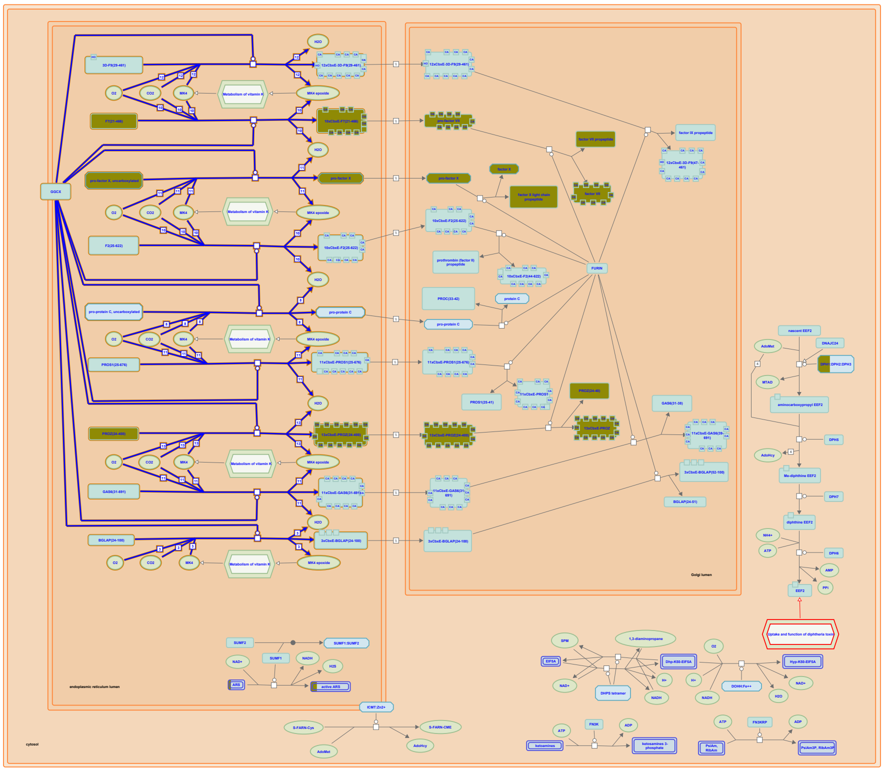

Cellular compartments: endoplasmic reticulum membrane.

Gamma-carboxylation of a cluster of glutamate residues near the amino termini of thrombin, factor VII, factor IX, factor X, protein C, protein S, protein Z, and Gas 6 is required for these proteins to bind Ca++ and function efficiently in blood clotting. A single enzyme, vitamin K-dependent gamma-carboxylase, catalyzes the gamma-carboxylation of all eight proteins involved in clotting (Morris et al. 1995; Brenner et al. 1998; Spronk et al. 2000). In the carboxylation reaction, the enzyme binds its substrate protein via a sequence motif on the amino terminal side of the glutamate residues to be carboxylated (Furie et al. 1999), then processively carboxylates all glutamates in the cluster before releasing the substrate (Morris et al. 1995; Berkner 2000; Stenina et al. 2001). The reaction occurs in the endoplasmic reticulum (Bristol et al. 1996).

References

Berkner KL (2000). The vitamin K-dependent carboxylase. J Nutr, 130, 1877-80.

Brenner B, Sanchez-Vega B, Wu SM, Lanir N, Stafford DW & Solera J (1998). A missense mutation in gamma-glutamyl carboxylase gene causes combined deficiency of all vitamin K-dependent blood coagulation factors. Blood, 92, 4554-9.

Furie B, Bouchard BA & Furie BC (1999). Vitamin K-dependent biosynthesis of gamma-carboxyglutamic acid. Blood, 93, 1798-808.

Stenina O, Pudota BN, McNally BA, Hommema EL & Berkner KL (2001). Tethered processivity of the vitamin K-dependent carboxylase: factor IX is efficiently modified in a mechanism which distinguishes Gla's from Glu's and which accounts for comprehensive carboxylation in vivo. Biochemistry, 40, 10301-9.

Spronk HM, Farah RA, Buchanan GR, Vermeer C & Soute BA (2000). Novel mutation in the gamma-glutamyl carboxylase gene resulting in congenital combined deficiency of all vitamin K-dependent blood coagulation factors. Blood, 96, 3650-2.

Edit history

Date	Action	Author
2005-03-17	Authored	D'Eustachio P
2005-03-17	Created	D'Eustachio P
2018-08-21	Edited	D'Eustachio P
2018-08-30	Modified	Croft D

Elements found in this pathway

Input	UniProt Id	Input	UniProt Id	Input	UniProt Id
F10	P00742	F7	P08709	Proz	P22891

13. Regulation of gene expression by Hypoxia-inducible Factor (R-HSA-1234158)

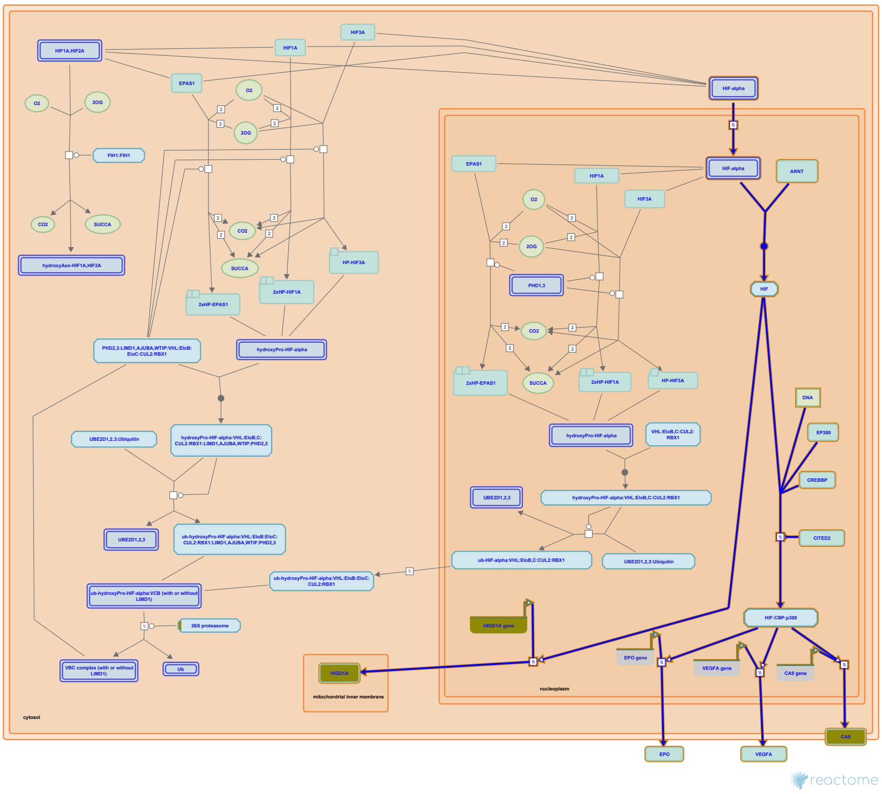

Cellular compartments: nucleoplasm.

HIF-alpha (HIF1A, HIF2A (EPAS1), HIF3A) is translocated to the nucleus, possibly by two pathways: importin 4/7 (Chachami et al. 2009) and importin alpha/beta (Depping et al. 2008). Once in the nucleus HIF-alpha heterodimerizes with HIF-beta (ARNT) (Wang et al. 1995, Jiang et al. 1996, Tian et al. 1997, Gu et al. 1998, Erbel et al. 2003) and recruits CBP and p300 to promoters of target genes (Ebert and Bunn 1998, Kallio et al. 1998, Ema et al. 1999, Gu et al. 2001, Dames et al. 2002, Freedman et al. 2002).

References

Chachami G, Paraskeva E, Mingot JM, Braliou GG, Görlich D & Simos G (2009). Transport of hypoxia-inducible factor HIF-1alpha into the nucleus involves importins 4 and 7. Biochem Biophys Res Commun, 390, 235-40.

Kallio PJ, Okamoto K, O'Brien S, Carrero P, Makino Y, Tanaka H & Poellinger L (1998). Signal transduction in hypoxic cells: inducible nuclear translocation and recruitment of the CBP/p300 coactivator by the hypoxia-inducible factor-1alpha. EMBO J, 17, 6573-86.

Depping R, Steinhoff A, Schindler SG, Friedrich B, Fagerlund R, Metzen E, ... Köhler M (2008). Nuclear translocation of hypoxia-inducible factors (HIFs): involvement of the classical importin alpha/beta pathway. Biochim Biophys Acta, 1783, 394-404.

Tian H, McKnight SL & Russell DW (1997). Endothelial PAS domain protein 1 (EPAS1), a transcription factor selectively expressed in endothelial cells. Genes Dev, 11, 72-82.

Jiang BH, Rue E, Wang GL, Roe R & Semenza GL (1996). Dimerization, DNA binding, and transactivation properties of hypoxia-inducible factor 1. J Biol Chem, 271, 17771-8.

Edit history

Date	Action	Author
2011-03-09	Edited	May B
2011-03-09	Authored	May B
2011-03-18	Created	May B
2012-05-19	Reviewed	Rantanen K
2018-08-30	Modified	Croft D

Elements found in this pathway

Input	UniProt Id	Input	UniProt Id	Input	UniProt Id
Car9	Q16790	Higd1a	Q9Y241		

Input	Ensembl Id	Input	Ensembl Id	Input	Ensembl Id
Higd1a	ENSG00000181061				

14. DDX58/IFIH1-mediated induction of interferon-alpha/beta (R-HSA-168928)

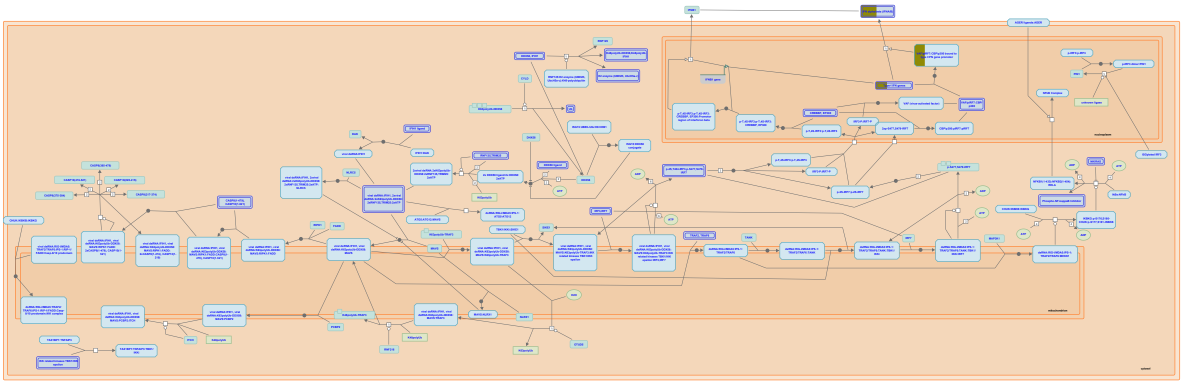

Cellular compartments: mitochondrial outer membrane.

RIG-I-like helicases (RLHs) the retinoic acid inducible gene-I (RIG-I) and melanoma differentiation associated gene 5 (MDA5) are RNA helicases that recognize viral RNA present within the cytoplasm. Functionally RIG-I and MDA5 positively regulate the IFN genes in a similar fashion, however they differ in their response to different viral species. RIG-I is essential for detecting influenza virus, Sendai virus, VSV and Japanese encephalitis virus (JEV), whereas MDA5 is essential in sensing encephalomyocarditis virus (EMCV), Mengo virus and Theiler's virus, all of which belong to the picornavirus family. RIG-I and MDA5 signalling results in the activation of IKK epsilon and (TKK binding kinase 1) TBK1, two serine/threonine kinases that phosphorylate interferon regulatory factor 3 and 7 (IRF3 and IRF7). Upon phosphorylation, IRF3 and IRF7 translocate to the nucleus and subsequently induce interferon alpha (IFNA) and interferon beta (IFNB) gene transcription.

References

Honda K, Yanai H, Takaoka A & Taniguchi T (2005). Regulation of the type I IFN induction: a current view. Int Immunol, 17, 1367-78.

Loo YM, Fornek J, Crochet N, Bajwa G, Perwitasari O, Martinez-Sobrido L, ... Gale M Jr (2008). Distinct RIG-I and MDA5 signaling by RNA viruses in innate immunity. J Virol, 82, 335-45.

Bowie AG & Unterholzner L (2008). Viral evasion and subversion of pattern-recognition receptor signalling. Nat Rev Immunol, 8, 911-22.

Yoneyama M & Fujita T (2008). Structural mechanism of RNA recognition by the RIG-I-like receptors. Immunity, 29, 178-81.

Yoneyama M & Fujita T (2007). RIG-I family RNA helicases: cytoplasmic sensor for antiviral innate immunity. Cytokine Growth Factor Rev, 18, 545-51.

Edit history

Date	Action	Author
2005-11-22	Created	de Bono B
2010-08-02	Edited	Garapati P V
2010-08-02	Authored	Garapati P V
2010-10-30	Reviewed	Akira S, Kawai T
2018-08-23	Modified	Schmidt EE

Elements found in this pathway

Input	UniProt Id	Input	UniProt Id	Input	UniProt Id
Ifna11	P01570	Ifna4	P01569, P05014	Ifna5	P01569, P05013, P01563
Ifna6	P05013	Ifna7	P01567, P05013		

Input	Ensembl Id	Input	Ensembl Id	Input	Ensembl Id
Ifna4	ENSG00000236637	Ifna5	ENSG00000147873	Ifna6	ENSG00000120235
Ifna7	ENSG00000214042				

15. PTK6 Expression (R-HSA-8849473)

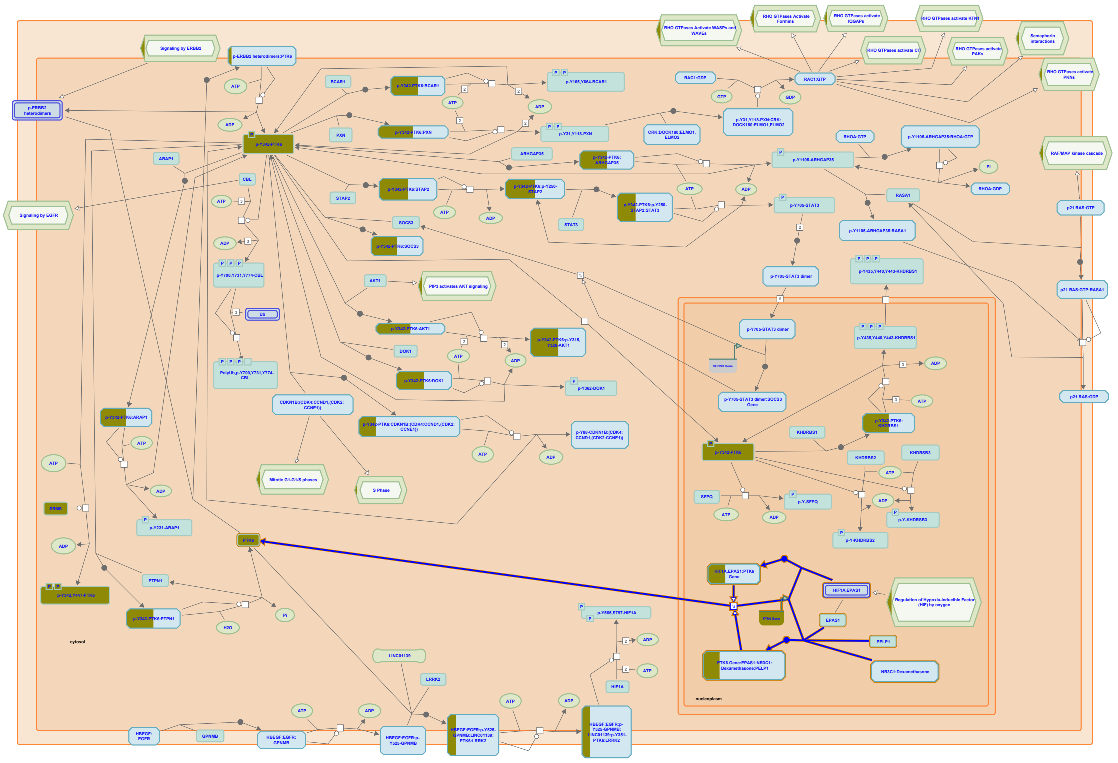

Levels of PTK6 increase under hypoxic conditions due to direct transcriptional regulation of PTK6 gene by hypoxia inducible transcription factors (HIFs) (Regan Anderson et al. 2013). PTK6 protein levels are also rapidly stabilized in hypoxic conditions in a HIF-independent manner (Pires et al. 2014). It has also been shown that PTK6 is ubiquitinated in normoxic conditions by a so far unknown E3 ligase (Pires et al. 2014).

References

Regan Anderson TM, Peacock DL, Daniel AR, Hubbard GK, Lofgren KA, Girard BJ, ... Lange CA (2013). Breast tumor kinase (Brk/PTK6) is a mediator of hypoxia-associated breast cancer progression. Cancer Res., 73, 5810-20.

Pires IM, Blokland NJ, Broos AW, Poujade FA, Senra JM, Eccles SA, ... Hammond EM (2014). HIF-1-independent hypoxia-induced rapid PTK6 stabilization is associated with increased motility and invasion. Cancer Biol. Ther., 15, 1350-7.

Edit history

Date	Action	Author
2015-12-16	Created	Orlic-Milacic M
2016-01-05	Edited	Orlic-Milacic M
2016-01-05	Authored	Orlic-Milacic M
2016-02-07	Reviewed	Pires IM
2018-08-30	Modified	Croft D

Elements found in this pathway

Input	UniProt Id	Input	UniProt Id	Input	UniProt Id
Ptk6	Q13882				

Input	Ensembl Id	Input	Ensembl Id	Input	Ensembl Id
Ptk6	ENSG00000101213				

16. PTK6 Down-Regulation (R-HSA-8849472)

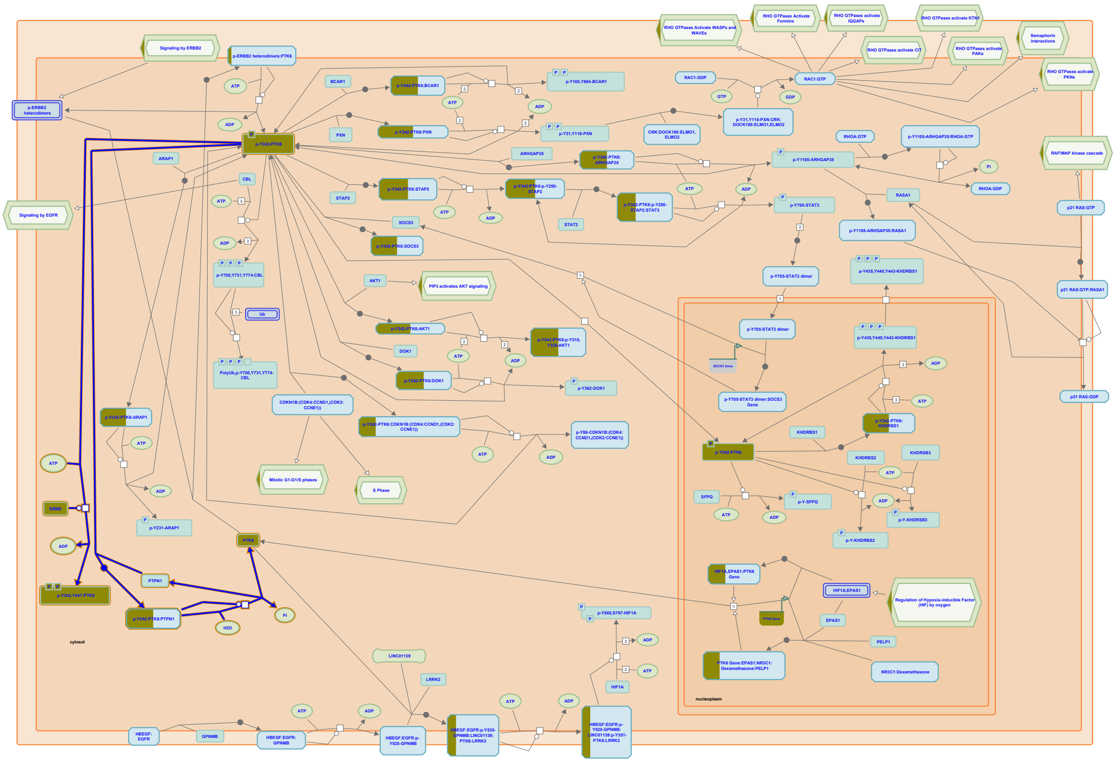

The kinase activity of PTK6 is negatively regulated by both PTPN1 phosphatase (Fan et al. 2013), which dephosphorylates tyrosine Y342 of PTK6, and SRMS kinase (Fan et al. 2015), which phosphorylates PTK6 on tyrosine residue Y447.

References

Fan G, Aleem S, Yang M, Miller WT & Tonks NK (2015). Protein-tyrosine Phosphatase and Kinase Specificity in Regulation of SRC and Breast Tumor Kinase. J. Biol. Chem., 290, 15934-47.

Fan G, Lin G, Lucito R & Tonks NK (2013). Protein-tyrosine phosphatase 1B antagonized signaling by insulin-like growth factor-1 receptor and kinase BRK/PTK6 in ovarian cancer cells. J. Biol. Chem., 288, 24923-34.

Edit history

Date	Action	Author
2015-12-16	Created	Orlic-Milacic M
2016-01-05	Edited	Orlic-Milacic M
2016-01-05	Authored	Orlic-Milacic M
2016-02-07	Reviewed	Pires IM
2018-08-23	Modified	Schmidt EE

Elements found in this pathway

Input	UniProt Id	Input	UniProt Id	Input	UniProt Id
Ptk6	Q13882	Srms	Q9H3Y6		

17. Loss of phosphorylation of MECP2 at T308 (R-HSA-9022535)

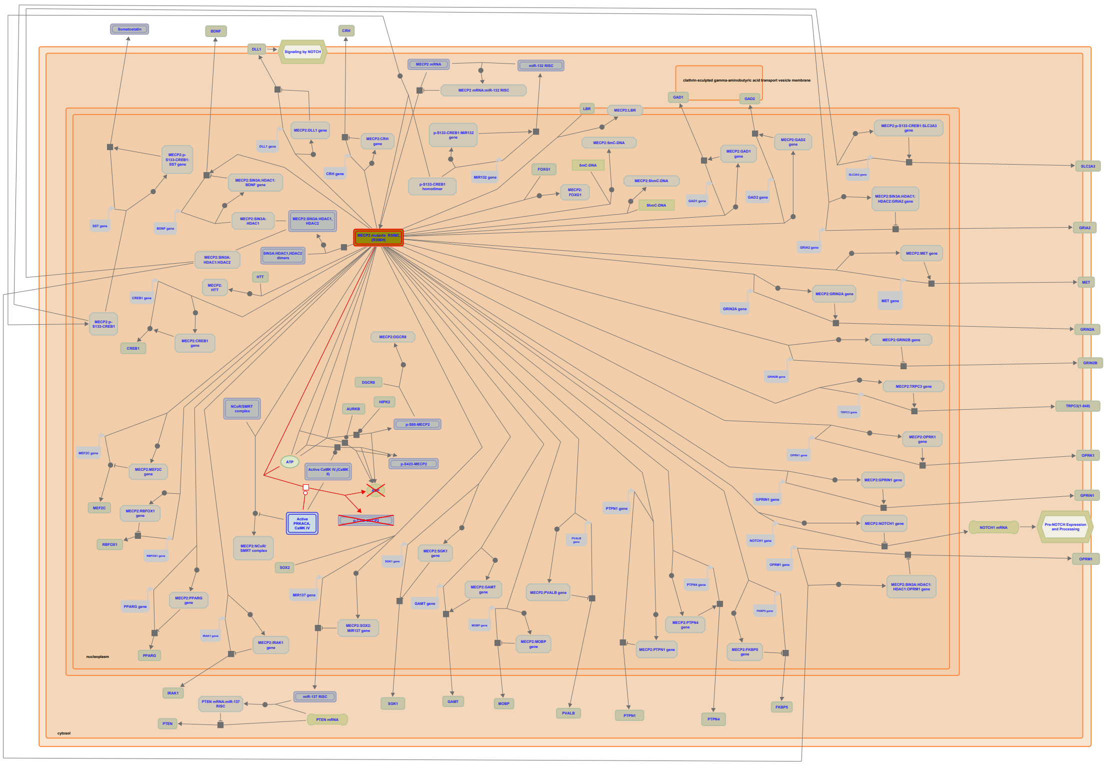

reactome

Cellular compartments: nucleoplasm.

Diseases: Rett syndrome.

Missense mutations of methyl-CpG-binding protein 2 (MECP2) in the vicinity of its threonine T308 phosphorylation site can negatively affect the ability of MECP2 to be phosphorylated at T308 in response to neuronal membrane depolarization (neuronal activity) (Ebert et al. 2013).

References

Ebert DH, Gabel HW, Robinson ND, Kastan NR, Hu LS, Cohen S, ... Greenberg ME (2013). Activity-dependent phosphorylation of MeCP2 threonine 308 regulates interaction with NCoR. Nature, 499, 341-5.

Edit history

Date	Action	Author
2017-09-25	Created	Orlic-Milacic M
2017-10-03	Authored	Orlic-Milacic M
2018-08-07	Reviewed	Christodoulou J, Krishnaraj R
2018-08-08	Edited	Orlic-Milacic M
2018-08-30	Modified	Croft D

Elements found in this pathway

Input	UniProt Id	Input	UniProt Id	Input	UniProt Id
Mecp2	P51608-2, P51608-1				

18. Loss of function of MECP2 in Rett syndrome (R-HSA-9005891)

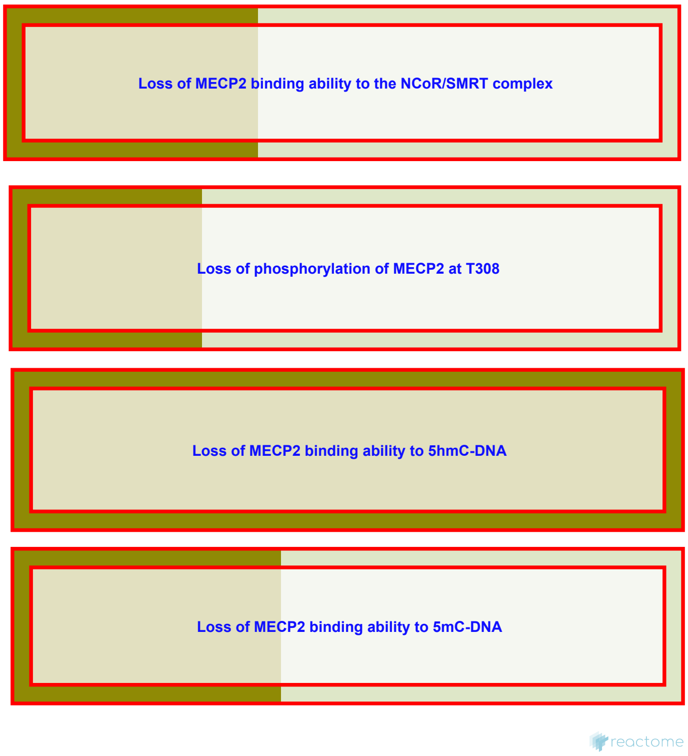

Diseases: Rett syndrome.

Loss of function mutations in methyl-CpG-binding protein 2 (MECP2), an epigenetic regulator of transcription, are the major cause of Rett syndrome, a neurodevelopmental disorder that affects 1 in 10,000-15,000 female births. The symptoms of Rett syndrome appear after 6-18 months of apparently normal postnatal development and include regression of acquired language and motor skills, stereotypic hand movements, intellectual disability, epileptic seizures and respiratory disturbances. Besides Rett syndrome, aberrant MECP2 expression is implicated as an underlying cause of other neuropsychiatric disorders (reviewed by Banerjee et al. 2012, Ebert and Greenberg 2013, Lyst and Bird 2015). Only functionally characterized MECP2 mutations are annotated. For a comprehensive list of MECP2 mutations reported in Rett syndrome, please refer to the RettBASE (http://mecp2.chw.edu.au), a database dedicated to curation of disease variants of MECP2, CDKL5 and FOXG1 in Rett syndrome (Krishnaraj et al. 2017).

References

Banerjee A, Castro J & Sur M (2012). Rett syndrome: genes, synapses, circuits, and therapeutics. Front Psychiatry, 3, 34.

Lyst MJ & Bird A (2015). Rett syndrome: a complex disorder with simple roots. Nat. Rev. Genet., 16, 261-75.

Ebert DH & Greenberg ME (2013). Activity-dependent neuronal signalling and autism spectrum disorder. Nature, 493, 327-37.

Krishnaraj R, Ho G & Christodoulou J (2017). RettBASE: Rett syndrome database update. Hum. Mutat., 38, 922-931.

RettBASE. Retrieved from http://mecp2.chw.edu.au/

Edit history

Date	Action	Author
2017-05-11	Created	Orlic-Milacic M
2017-10-03	Authored	Orlic-Milacic M
2018-08-07	Reviewed	Christodoulou J, Krishnaraj R
2018-08-08	Edited	Orlic-Milacic M
2018-09-05	Modified	Shorser S

Elements found in this pathway

Input	UniProt Id	Input	UniProt Id	Input	UniProt Id
Mecp2	P51608-2, P51608-1	Ncor2	Q9Y618		

19. Pervasive developmental disorders (R-HSA-9005895)

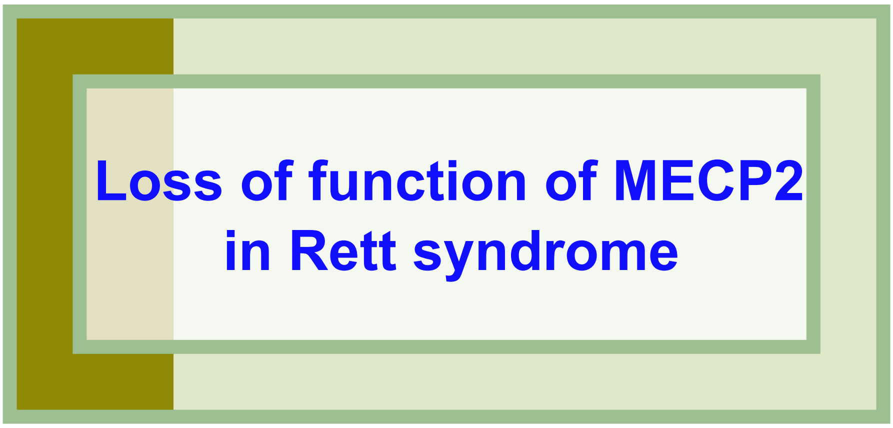

Diseases: pervasive developmental disorder.

Pervasive developmental disorders (PDDs) largely overlap with the autism spectrum disorders (ASDs). PDDs manifest in childhood and mainly affect social interaction, including communication and behavior. PDDs can be caused by mutations in genes involved in brain development and function, environmental insults, or the combination of environmental factors and genetic susceptibility. For review of this topic, please refer to Picket and London 2005, Currenti 2010, Elsabbagh et al. 2012, Ferreri 2014.

References

Pickett J & London E (2005). The neuropathology of autism: a review. J. Neuropathol. Exp. Neurol., 64, 925-35.

Ferreri M (2014). [Pervasive developmental disorders]. Rev Prat, 64, 481-6.

Elsabbagh M, Divan G, Koh YJ, Kim YS, Kauchali S, Marcín C, ... Fombonne E (2012). Global prevalence of autism and other pervasive developmental disorders. Autism Res, 5, 160-79.

Currenti SA (2010). Understanding and determining the etiology of autism. Cell. Mol. Neurobiol., 30 , 161-71.

Edit history

Date	Action	Author
2017-05-11	Created	Orlic-Milacic M
2017-10-03	Authored	Orlic-Milacic M
2018-08-08	Edited	Orlic-Milacic M

Date	Action	Author
2018-08-09	Modified	Orlic-Milacic M

Elements found in this pathway

Input	UniProt Id	Input	UniProt Id	Input	UniProt Id
Mecp2	P51608-2, P51608-1	Ncor2	Q9Y618		

20. Gamma-carboxylation, transport, and amino-terminal cleavage of proteins (R-HSA-159854)

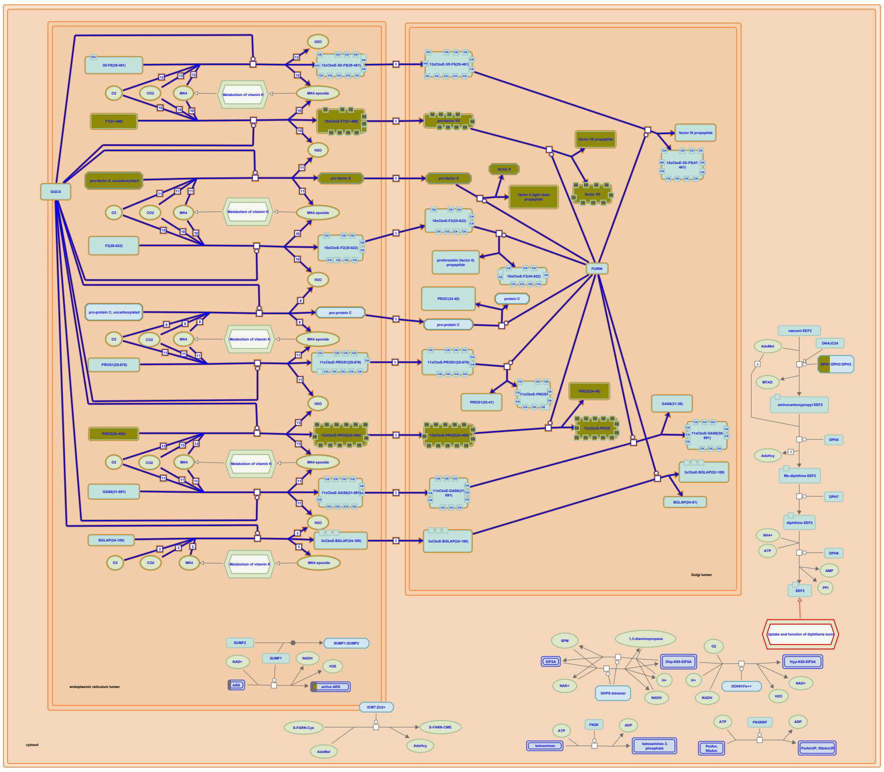

A number of proteins, including eight required for normal blood clot formation and its regulation (Prothrombin (factor II), factor VII, factor IX, factor X, protein C, protein S, protein Z, and Gas6) share a sequence motif rich in glutamate residues near their amino termini. Carboxylation of the glutamate residues within this motif followed by removal of an aminoterminal propeptide is required for each of these proteins to function. These modifications occur as the proteins move through the endoplasmic reticulum and Golgi apparatus.

Edit history

Date	Action	Author
2005-03-17	Authored	D'Eustachio P
2005-03-17	Created	D'Eustachio P
2018-08-21	Edited	D'Eustachio P
2018-08-21	Reviewed	Stafford DW
2018-08-23	Modified	Schmidt EE

Elements found in this pathway

Input	UniProt Id	Input	UniProt Id	Input	UniProt Id
F10	P00742	F7	P08709	Proz	P22891

21. TYSND1 cleaves peroxisomal proteins (R-HSA-9033500)

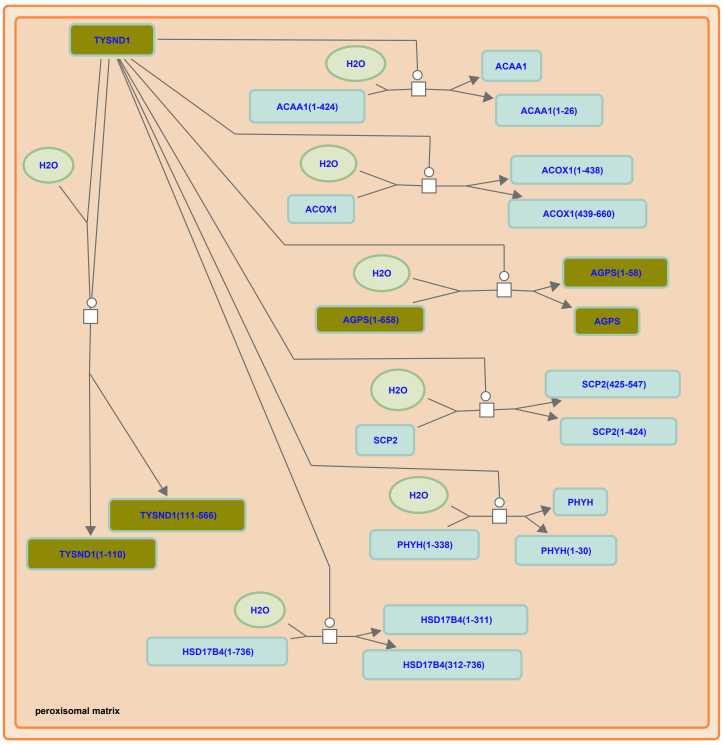

Cellular compartments: peroxisomal matrix.

After proteins are imported into the peroxisome a subset of proteins are cleaved by the protease TYSND1 (Okumoto et al. 2011). Based onmutagenesis of human TYSND1 (Okumoto et al. 2011) and the homolog in Arabidopsis (Schuhmann et al. 2008), TYSND1 appears to be a trypsin-like serine protease containing a conserved histidine aspartate serine triad essential for catalysis. Mice lacking Tysnd1 have reduced peroxisomal localization of some peroxisomal enzymes and exhibit reduced beta-oxidation of fatty acids and metabolism of phytanic acid (Mizuno et al. 2013). Male mice lacking Tysnd1 are sterile due to sperm that lack acrosomal caps.

References

Okumoto K, Kametani Y & Fujiki Y (2011). Two proteases, trypsin domain-containing 1 (Tysnd1) and peroxisomal lon protease (PsLon), cooperatively regulate fatty acid -oxidation in peroxisomal matrix. J. Biol. Chem., 286, 44367-79.

Mizuno Y, Ninomiya Y, Nakachi Y, Iseki M, Iwasa H, Akita M, ... Okazaki Y (2013). Tysnd1 deficiency in mice interferes with the peroxisomal localization of PTS2 enzymes, causing lipid metabolic abnormalities and male infertility. PLoS Genet., 9, e1003286.

Schuhmann H, Huesgen PF, Gietl C & Adamska I (2008). The DEG15 serine protease cleaves peroxisomal targeting signal 2-containing proteins in Arabidopsis. Plant Physiol., 148, 1847-56.

Edit history

Date	Action	Author
2017-12-22	Edited	May B
2017-12-22	Authored	May B

Date	Action	Author
2017-12-22	Created	May B
2018-02-13	Reviewed	Van Veldhoven PP, Fransen M
2018-03-12	Reviewed	Azevedo JE
2018-08-24	Modified	Schmidt EE

Elements found in this pathway

Input	UniProt Id	Input	UniProt Id	Input	UniProt Id
Agps	O00116	Sar1a	Q2T9J0	Tysnd1	Q2T9J0

22. Regulation of MECP2 expression and activity (R-HSA-9022692)

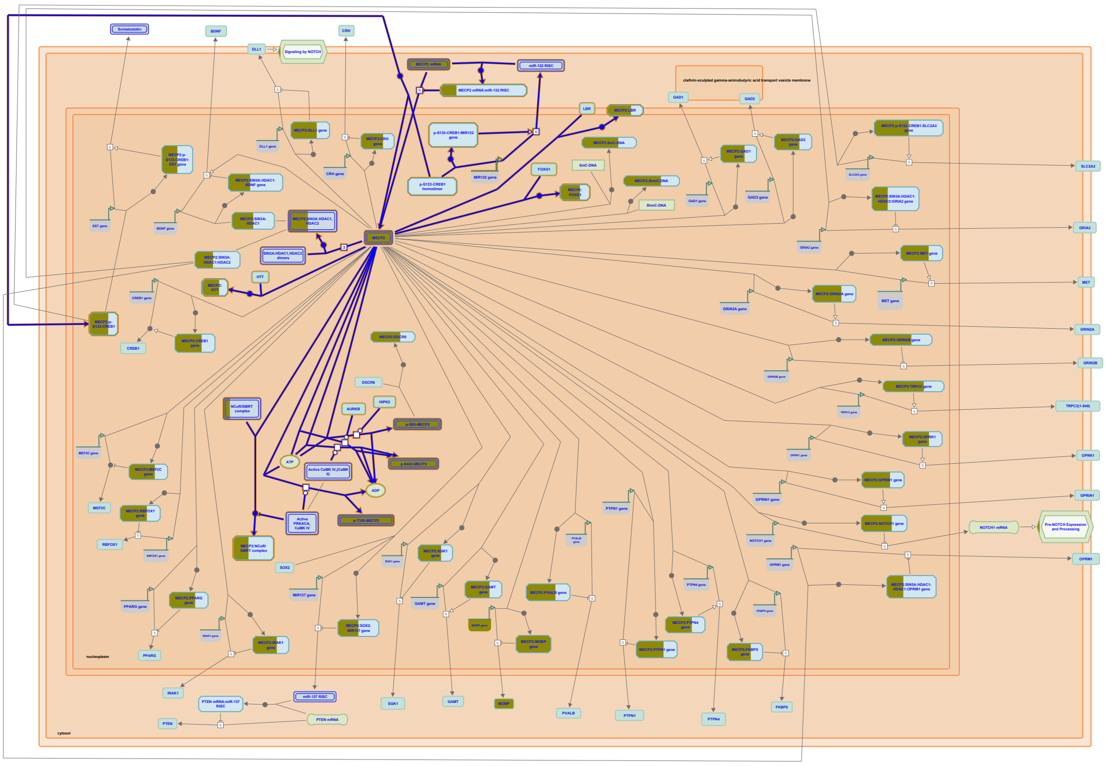

Transcription of the MECP2 gene is known to be regulated by methylation of the promoter and the first intron, but the responsible methyltransferases are not known (Nagarajan et al. 2008, Franklin et al. 2010, Liyanage et al. 2013).

Translation of MECP2 mRNA is negatively regulated by the microRNA miR-132. Transcription of miR-132 is regulated by BDNF signaling, through an unknown mechanism (Klein et al. 2007, Su et al. 2015).

Binding of MECP2 to other proteins and to DNA is regulated by posttranslational modifications, of which phosphorylation has been best studied. Calcium dependent protein kinases, PKA and CaMK IV, activated by neuronal membrane depolarization, phosphorylate MECP2 at threonine residue T308 (corresponding to T320 in the longer MECP2 splicing isoform, MECP2_e1). Phosphorylation at T308 correlates with neuronal activity and inhibits binding of MECP2 to the nuclear receptor co-repressor complex (NCoR/SMRT) (Ebert et al. 2013). In resting neurons, MECP2 is phosphorylated at serine residue S80, which results in a decreased association of MECP2 with chromatin. Nuclear serine/threonine protein kinase HIPK2 phosphorylates MECP2 on serine residue S80 (Bracaglia et al. 2009). In activity-induced neurons, upon neuronal membrane depolarization, MECP2 S80 becomes dephosphorylated, and MECP2 acquires phosphorylation on serine S423 (corresponding to mouse Mecp2 serine S421). CaMK IV is one of the kinases that can phosphorylate MECP2 on S423. Phosphorylation of MECP2 at S423 increases MECP2 binding to chromatin (Zhou et al. 2006, Tao et al. 2009, Qiu et al. 2012). AURKB phosphorylates MECP2 at serine residue S423 in dividing adult neuronal progenitor cells (Li et al. 2014).

Besides binding to the NCoR/SMRT co-repressor complex (Lyst et al. 2013, Ebert et al. 2013), MECP2 binds the SIN3A co-repressor complex. This interaction involves the transcriptional repressor domain of MECP2 and the amino terminal part of the HDAC interaction domain (HID) of SIN3A. HDAC1 and HDAC2 are part of the SIN3A co-repressor complex that co-immunoprecipitates with MECP2 (Nan et al. 1998). While binding of MECP2 to SIN3A at target genes is associated with transcriptional repression, binding to CREB1 at target genes is associated with transcriptional activation (Chahrour et al. 2008, Chen et al. 2013). Function of MECP2 can be affected by binding to FOXG1, another gene mutated in Rett syndrome besides MECP2 and CDKL5 (Dastidar et al. 2012), and HTT (Huntingtin) (McFarland et al. 2013). The subnuclear localization of MECP2 may be affected by binding to the Lamin B receptor (LBR) (Guarda et al. 2009).

References

Bracaglia G, Conca B, Bergo A, Rusconi L, Zhou Z, Greenberg ME, ... Kilstrup-Nielsen C (2009). Methyl-CpG-binding protein 2 is phosphorylated by homeodomain-interacting protein kinase 2 and contributes to apoptosis. EMBO Rep., 10, 1327-33.

Chahrour M, Jung SY, Shaw C, Zhou X, Wong ST, Qin J & Zoghbi HY (2008). MeCP2, a key contributor to neurological disease, activates and represses transcription. Science, 320, 1224-9.

Chen Y, Shin BC, Thamotharan S & Devaskar SU (2013). Creb1-Mecp2-(m)CpG complex transactivates postnatal murine neuronal glucose transporter isoform 3 expression. Endocrinology, 154, 1598-611.

Dastidar SG, Bardai FH, Ma C, Price V, Rawat V, Verma P, ... D'Mello SR (2012). Isoform-specific toxicity of Mecp2 in postmitotic neurons: suppression of neurotoxicity by FoxG1. J. Neurosci., 32, 2846-55.

Ebert DH, Gabel HW, Robinson ND, Kastan NR, Hu LS, Cohen S, ... Greenberg ME (2013). Activity-dependent phosphorylation of MeCP2 threonine 308 regulates interaction with NCoR. Nature, 499, 341-5.

Edit history

Date	Action	Author
2017-09-25	Created	Orlic-Milacic M
2017-10-03	Authored	Orlic-Milacic M
2018-08-07	Reviewed	Christodoulou J, Krishnaraj R
2018-08-08	Edited	Orlic-Milacic M
2018-08-23	Modified	Schmidt EE

Elements found in this pathway

Input	UniProt Id	Input	UniProt Id	Input	UniProt Id
Mecp2	P51608-2, P51608-1	Ncor2	Q9Y618		

Input	Ensembl Id	Input	Ensembl Id	Input	Ensembl Id
Mecp2	ENST00000303391, ENST00000453960				

23. TP53 Regulates Transcription of Cell Cycle Genes (R-HSA-6791312)

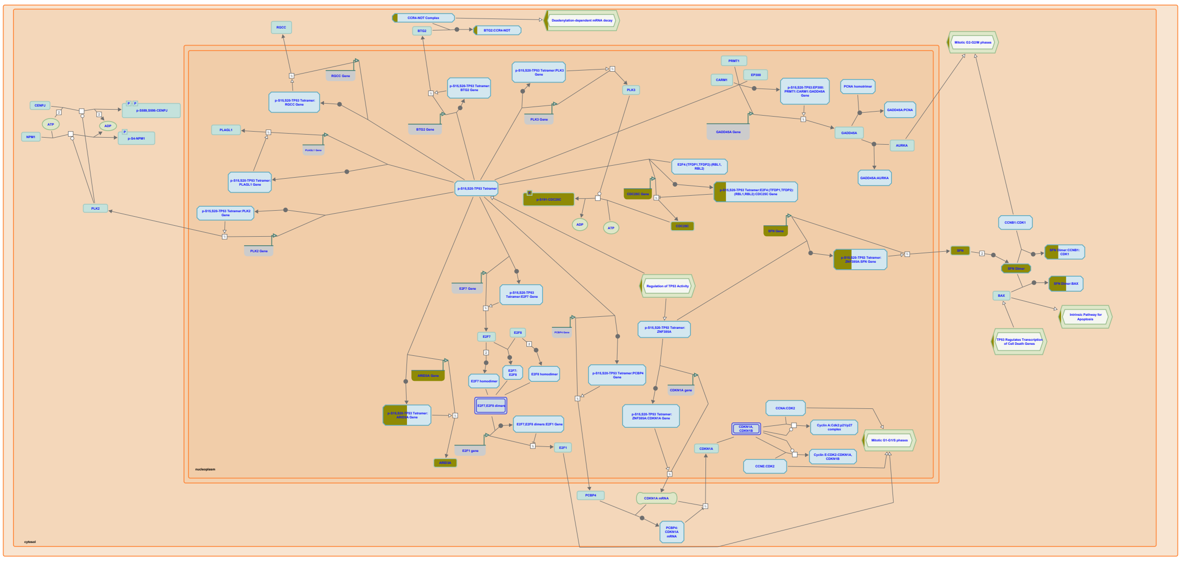

Under a variety of stress conditions, TP53 (p53), stabilized by stress-induced phosphorylation at least on S15 and S20 serine residues, can induce the transcription of genes involved in cell cycle arrest. Cell cycle arrest provides cells an opportunity to repair the damage before division, thus preventing the transmission of genetic errors to daughter cells. In addition, it allows cells to attempt a recovery from the damage and survive, preventing premature cell death.

TP53 controls transcription of genes involved in both G1 and G2 cell cycle arrest. The most prominent TP53 target involved in G1 arrest is the inhibitor of cyclin-dependent kinases CDKN1A (p21). CDKN1A is one of the earliest genes induced by TP53 (El-Deiry et al. 1993). CDKN1A binds and inactivates CDK2 in complex with cyclin A (CCNA) or E (CCNE), thus preventing G1/S transition (Harper et al. 1993). Nevertheless, under prolonged stress, the cell destiny may be diverted towards an apoptotic outcome. For instance, in case of an irreversible damage, TP53 can induce transcription of an RNA binding protein PCBP4, which can bind and destabilize CDKN1A mRNA, thus alleviating G1 arrest and directing the affected cell towards G2 arrest and, possibly, apoptosis (Zhu and Chen 2000, Scoumanne et al. 2011). Expression of E2F7 is directly induced by TP53. E2F7 contributes to G1 cell cycle arrest by repressing transcription of E2F1, a transcription factor that promotes expression of many genes needed for G1/S transition (Aksoy et al. 2012, Carvajal et al. 2012). ARID3A is a direct transcriptional target of TP53 (Ma et al. 2003) that may promote G1 arrest by cooperating with TP53 in induction of CDKN1A transcription (Lestari et al. 2012). However, ARID3A may also promote G1/S transition by stimulating transcriptional activity of E2F1 (Suzuki et al. 1998, Peeper et al. 2002).

TP53 contributes to the establishment of G2 arrest by inducing transcription of GADD45A and SFN, and by inhibiting transcription of CDC25C. TP53 induces GADD45A transcription in cooperation with chromatin modifying enzymes EP300, PRMT1 and CARM1 (An et al. 2004). GADD45A binds Aurora kinase A (AURKA), inhibiting its catalytic activity and preventing AURKA-mediated G2/M transition (Shao et al. 2006, Sanchez et al. 2010). GADD45A also forms a complex with PCNA. PCNA is involved in both normal and repair DNA synthesis. The effect of GADD45 interaction with PCNA, if any, on S phase progression, G2 arrest and DNA repair is not known (Smith et al. 1994, Hall et al. 1995, Sanchez et al. 2010, Kim et al. 2013). SFN (14-3-3-sigma) is induced by TP53 (Hermeking et al. 1997) and contributes to G2 arrest by binding to the complex of CDK1 and CCNB1 (cyclin B1) and preventing its translocation to the nucleus. Phosphorylation of a number of nuclear proteins by the complex of CDK1 and CCNB1 is needed for G2/M transition (Chan et al. 1999). While promoting G2 arrest, SFN can simultaneously inhibit apoptosis by binding to BAX and preventing its translocation to mitochondria, a step involved in cytochrome C release (Samuel et al. 2001). TP53 binds the promoter of the CDC25C gene in cooperation with the transcriptional repressor E2F4 and represses CDC25C transcription, thus maintaining G2 arrest (St Clair et al. 2004, Benson et al. 2014).

Several direct transcriptional targets of TP53 are involved in cell cycle arrest but their mechanism of action is still unknown. BTG2 is induced by TP53, leading to cessation of cellular proliferation (Rouault et al. 1996, Duriez et al. 2002). BTG2 binds to the CCR4-NOT complex and promotes mRNA deadenylation activity of this complex. Interaction between BTG2 and CCR4-NOT is needed for the antiproliferative activity of BTG2, but the underlying mechanism has not been elucidated (Rouault et al. 1998, Mauxion et al. 2008, Horiuchi et al. 2009, Doidge et al. 2012, Ezzeddine et al. 2012). Two polo-like kinases, PLK2 and PLK3, are direct transcriptional targets of TP53. TP53-mediated induction of PLK2 may be important for prevention of mitotic catastrophe after spindle damage (Burns et al. 2003). PLK2 is involved in the regulation of centrosome duplication through phosphorylation of centrosome-related proteins CENPJ (Chang et al. 2010) and NPM1 (Krause and Hoffmann 2010). PLK2 is frequently transcriptionally silenced through promoter methylation in B-cell malignancies (Syed et al. 2006). Induction of PLK3 transcription by TP53 (Jen and Cheung 2005) may be important for coordination of M phase events through PLK3-mediated nuclear accumulation of CDC25C (Bahassi et al. 2004). RGCC is induced by TP53 and implicated in cell cycle regulation, possibly through its association with PLK1 (Saigusa et al. 2007). PLAGL1 (ZAC1) is a zinc finger protein directly transcriptionally induced by TP53 (Rozenfeld-Granot et al. 2002). PLAGL1 expression is frequently lost in cancer (Varrault et al. 1998) and PLAGL1 has been implicated in both cell cycle arrest and apoptosis (Spengler et al. 1997), but its mechanism of action remains unknown.

The zinc finger transcription factor ZNF385A (HZF) is a direct transcriptional target of TP53 that can form a complex with TP53 and facilitate TP53-mediated induction of CDKN1A and SFN (14-3-3 sigma) transcription (Das et al. 2007).

For a review of the role of TP53 in cell cycle arrest and cell cycle transcriptional targets of TP53, please refer to Riley et al. 2008, Murray-Zmijewski et al. 2008, Bieging et al. 2014, Kruiswijk et al. 2015.

References

el-Deiry WS, Tokino T, Velculescu VE, Levy DB, Parsons R, Trent JM, ... Vogelstein B (1993). WAF1, a potential mediator of p53 tumor suppression. Cell, 75, 817-25.

Harper JW, Adami GR, Wei N, Keyomarsi K & Elledge SJ (1993). The p21 Cdk-interacting protein Cip1 is a potent inhibitor of G1 cyclin-dependent kinases. Cell, 75, 805-16.

Zhu J & Chen X (2000). MCG10, a novel p53 target gene that encodes a KH domain RNA-binding protein, is capable of inducing apoptosis and cell cycle arrest in G(2)-M. Mol. Cell. Biol., 20, 5602-18.

Scoumanne A, Cho SJ, Zhang J & Chen X (2011). The cyclin-dependent kinase inhibitor p21 is regulated by RNA-binding protein PCBP4 via mRNA stability. Nucleic Acids Res., 39, 213-24.

Aksoy O, Chicas A, Zeng T, Zhao Z, McCurrach M, Wang X & Lowe SW (2012). The atypical E2F family member E2F7 couples the p53 and RB pathways during cellular senescence. Genes Dev., 26, 1546-57.

Edit history

Date	Action	Author
2015-08-20	Created	Orlic-Milacic M
2015-10-14	Edited	Orlic-Milacic M
2015-10-14	Authored	Orlic-Milacic M
2016-02-04	Reviewed	Zaccara S, Inga A
2018-08-24	Modified	Schmidt EE

Elements found in this pathway

Input	UniProt Id
Arid3a	Q99856
Sfn	P31947

Input	UniProt Id
Cdc25c	P30307

Input	UniProt Id
Rqcd1	Q92600

Input	Ensembl Id
Arid3a	ENSG00000116017

Input	Ensembl Id
Cdc25c	ENSG00000158402

Input	Ensembl Id
Sfn	ENSG00000175793

24. TWIK-related spinal cord K+ channel (TRESK) (R-HSA-1299344)

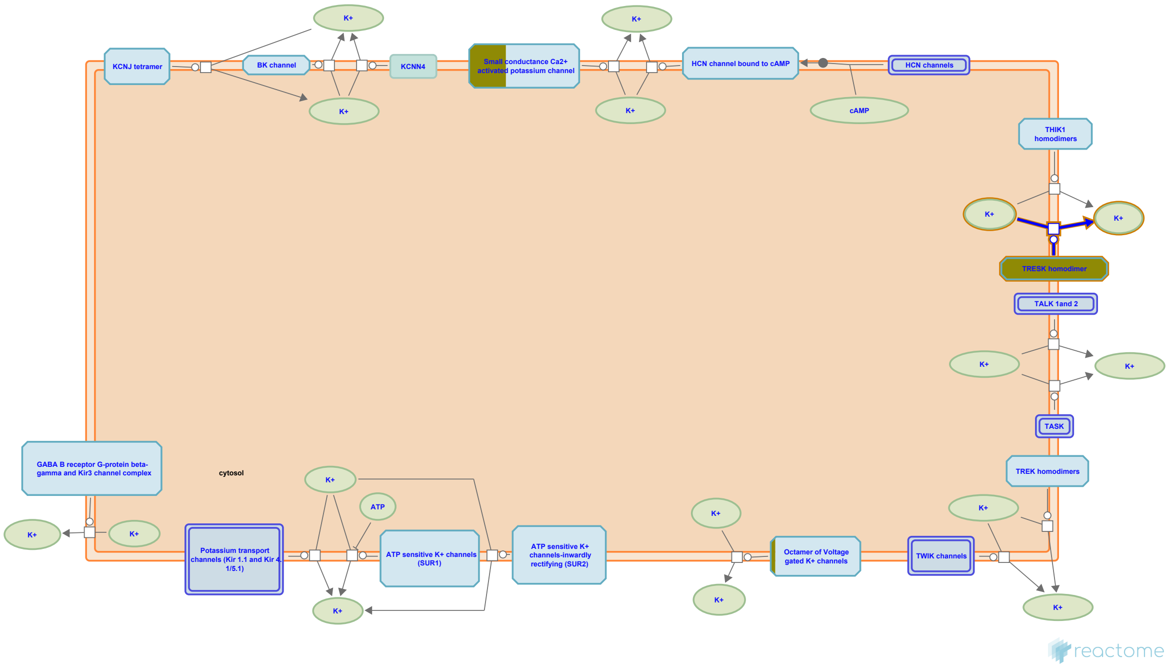

Cellular compartments: plasma membrane, cytosol, extracellular region.

TRESK subfamily of tandem domain K+ channels has one only member. TRESK is regulated by Ca/calmodulin dependent protein phosphatase, calcineurin.

References

Czirják G & Enyedi P (2010). TRESK background K(+) channel is inhibited by phosphorylation via two distinct pathways. J Biol Chem, 285, 14549-57.

Edit history

Date	Action	Author
2010-09-23	Reviewed	Jassal B
2011-05-22	Authored	Mahajan SS
2011-05-23	Edited	Mahajan SS
2011-05-23	Created	Mahajan SS
2018-08-23	Modified	Schmidt EE

Elements found in this pathway

Input	UniProt Id	Input	UniProt Id	Input	UniProt Id
Kcnk18	Q7Z418				

25. MECP2 regulates transcription factors (R-HSA-9022707)

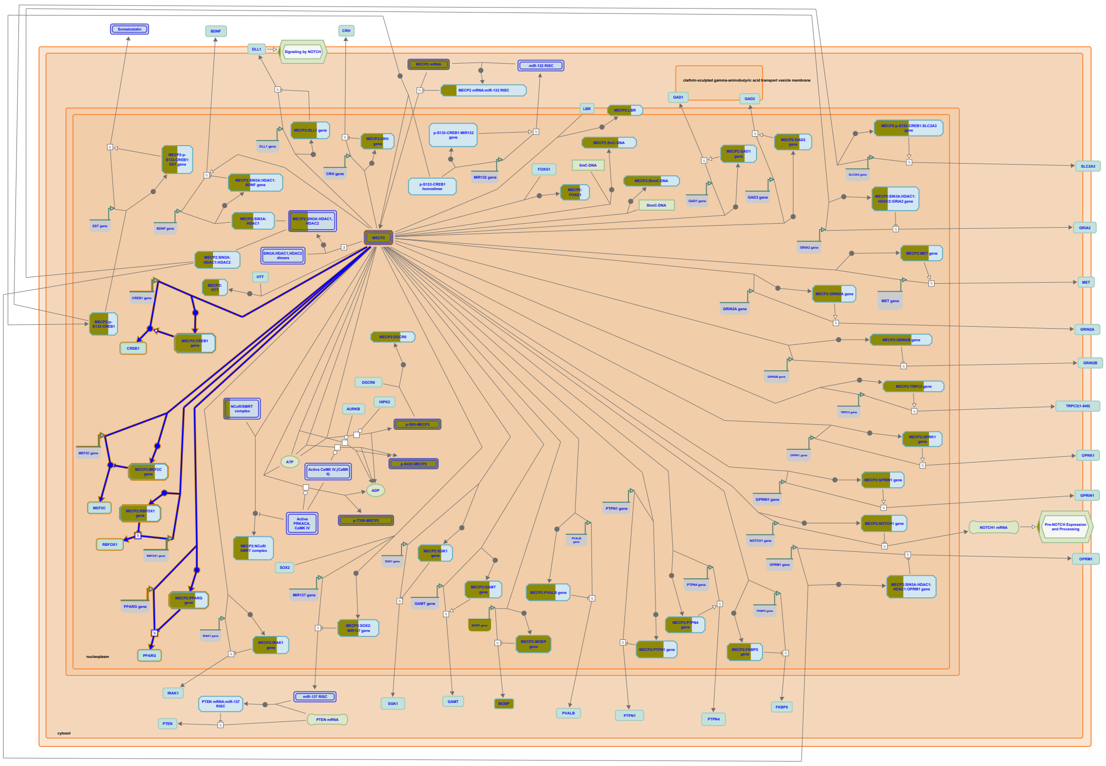

MECP2 regulates transcription of several transcription factors involved in functioning of the nervous system, such as CREB1, MEF2C, RBFOX1 (Chahrour et al. 2008) and PPARG (Mann et al. 2010, Joss Moore et al. 2011).

References

Chahrour M, Jung SY, Shaw C, Zhou X, Wong ST, Qin J & Zoghbi HY (2008). MeCP2, a key contributor to neurological disease, activates and represses transcription. Science, 320, 1224-9.

Joss-Moore LA, Wang Y, Ogata EM, Sainz AJ, Yu X, Callaway CW, ... Lane RH (2011). IUGR differentially alters MeCP2 expression and H3K9Me3 of the PPAR gene in male and female rat lungs during alveolarization. Birth Defects Res. Part A Clin. Mol. Teratol., 91, 672-81.

Mann J, Chu DC, Maxwell A, Oakley F, Zhu NL, Tsukamoto H & Mann DA (2010). MeCP2 controls an epigenetic pathway that promotes myofibroblast transdifferentiation and fibrosis. Gastroenterology, 138, 705-14, 714.e1-4.

Edit history

Date	Action	Author
2017-09-25	Created	Orlic-Milacic M
2017-10-03	Authored	Orlic-Milacic M
2018-08-07	Reviewed	Christodoulou J, Krishnaraj R
2018-08-08	Modified	Orlic-Milacic M
2018-08-08	Edited	Orlic-Milacic M

Elements found in this pathway

Input	UniProt Id	Input	UniProt Id	Input	UniProt Id
Mecp2	P51608-2, P51608-1				

6. Identifiers found

Input	UniProt Id	Input	UniProt Id	Input	UniProt Id
Acr	P10323	Agps	O00116	Arid3a	Q99856
Car9	Q16790	Cdc25c	P30307	F10	P00742
F7	P08709	Higd1a	Q9Y241	Ifna11	P01570
Ifna4	P01569, P05014	Ifna5	P01569, P05013, P01563	Ifna6	P05013
Ifna7	P01567, P05013	Kcnk18	Q7Z418	Mecp2	P51608-2, P51608-1
Ncor2	Q9Y618	Proz	P22891	Ptk6	Q13882
Rqcd1	Q92600	Sar1a	Q2T9J0	Sfn	P31947
Srms	Q9H3Y6	Tysnd1	Q2T9J0		

Input	Ensembl Id	Input	Ensembl Id	Input	Ensembl Id
Arid3a	ENSG00000116017	Cdc25c	ENSG00000158402	Higd1a	ENSG00000181061
Ifna4	ENSG00000236637	Ifna5	ENSG00000147873	Ifna6	ENSG00000120235
Ifna7	ENSG00000214042	Mecp2	ENST00000303391, ENST00000453960	Ptk6	ENSG00000101213
Sfn	ENSG00000175793				

Input	MIRBASE Id	Input	MIRBASE Id	Input	MIRBASE Id

7. Identifiers not found

1-Mar	1500015O10Rik	1600010M07Rik	1700012B09Rik	1700022H01Rik	1700031M16Rik	1700123M08Rik	2010005H15Rik
2310002F09Rik	2310007L24Rik	2310035C23Rik	2410015M20Rik	2610016A17Rik	2610206C17Rik	2900005J15Rik	3010001F23Rik
4833427G06Rik	4930415L06Rik	4930474N05Rik	4930511A02Rik	4931431F19Rik	4933402P03Rik	4933405L10Rik	4933422A05Rik
6430548M08Rik	6430584L05Rik	9230116N13Rik	A330040F15Rik	A430078G23Rik	A930018P22Rik	AU019823	AY358078
Abhd2	Adam26b	Agap1	Amer3	Ankib1	Ankrd13d	Ankrd49	Ap5m1
Arl15	Arrdc2	Astn2	Atpaf2	BC023829	BC100530	Bola3	Bpifb3
Brd9	Brip1os	Bsph2	C230037L18Rik	Ccdc124	Ccdc13	Ccdc130	Ccdc33
Ccser2	Cd209d	Cd59a	Cdpf1	Cdv3	Cend1	Cep112	Ces2e
Ces2f	Ces2g	Ces3a	ChkbCpt1b	Cnnm1	Cntnap5c	Cpxcr1	Cracr2b
Cramp1l	Crocc	Cyp3a11	Cypt10	Cypt4	Cypt9	D430041D05Rik	D8Ertd738e
Dach1	Dcaf12	Ddx43	Ddx59	Defb33	Dmrt2	Dnaic1	Dnal1
Dnttip1	Dpy19l2	E030002O03Rik	E130112N10Rik	Ehbp1l1	Enho	Enkd1	Enox1
Erp27	Fam111a	Fam118b	Fam131c	Fam166b	Fam26e	Fam46b	Fam53c
Fam65b	Fam71d	Fam76b	Fam89b	Fastkd1	Flcn	Foxi1	Foxp2
Fras1	Gfod2	Gid4	Gimap1	Gimap5	Gimap7	Gipc1	Glipr1l1
Glipr1l2	Gltpd2	Gm10639	Gm10778	Gm11346	Gm12295	Gm13275	Gm13285
Gm13287	Gm13288	Gm13289	Gm13290	Gm13939	Gm14744	Gm15056	Gm15772
Gm16062	Gm16501	Gm2087	Gm21944	Gm37013	Gm38666	Gm4861	Gm4952
Gm6026	Gm6040	Gm648	Gm8369	Gpatch3	Gpn2	Gpr173	Gpr88
H2-DMb1	Hn1l	Igsf5	Igsf6	Immp2l	Ina	Ipo7	Irf2bp1
Itgb2l	Jakmip3	Jtb	Kantr	Kctd14	Kdf1	Klhl34	Klk10
Klk11	Klk15	Klk1b1	Klk1b7-ps	Klk1b8	Klk9	Krit1	Layn
Lca5	Lonp1	Lrp3	Lrrc28	Lrrc39	Lrrc48	Lrrc63	Mamdc2
Map3k9	Mapk8ip2	Mapk8ip3	Mettl9	Mfsd1	Mfsd7a	Mir142	Mir142b
Mir152	Mir3969	Mir6942	Mir6985	Mir7036	Mir7067	Mir7239	Mir8113
Mir8116	Mmp27	Mrgprb8	Mrgprg	Mro	Ms4a4d	Ms4a6b	Mxd4
Myocd	Mypop	Myrfl	Myt1l	Nckap5	Nfe2l1	Nkain2	Nkx1-2
Npcd	Nup210l	Nutf2	Nutf2-ps1	Nutm2	Odf4	Ogfod3	Olfr1047
Olfr1048	Olfr1094	Olfr1095	Olfr1099	Olfr1100	Olfr1101	Olfr1102	Olfr1105
Olfr1106	Olfr1135	Olfr1136	Olfr1137	Olfr1182	Olfr1183	Olfr129	Olfr130
Olfr1305	Olfr131	Olfr132	Olfr133	Olfr1336	Olfr1344	Olfr1346	Olfr1348
Olfr1349	Olfr1388	Olfr1424	Olfr1425	Olfr1427	Olfr1499	Olfr1501	Olfr1537
Olfr170	Olfr176	Olfr177	Olfr178	Olfr196	Olfr197	Olfr281	Olfr282
Olfr320	Olfr367-ps	Olfr411	Olfr472	Olfr479	Olfr480	Olfr481	Olfr482
Olfr483	Olfr5	Olfr516	Olfr517	Olfr518	Olfr519	Olfr521	Olfr531
Olfr532	Olfr533	Olfr535	Olfr536	Olfr584	Olfr585	Olfr767	Olfr768
Olfr769	Olfr936	Olfr937	Olfr938	Olfr944	Olfr97	Omt2a	Omt2b
Oxnad1	Pax2	Pbsn	Pcdha7-g	Pcdhb18	Pcdhb19	Pcdhb20	Pcdhb21
Pcdhb22	Pcdhga1	Pcdhga2	Pcdhga3	Pcdhga4	Pcdhga5	Pcdhga6	Pcdhga7
Pcdhgb1	Pcdhgb2	Pcgf3	Pcid2	Pcp4	Pde6h	Pdlim4	Pgbd1
Pibf1	Pih1d2	Pkdrej	Pllp	Plscr3	Pogz	Ppdpf	Ppig
Ppp1r17	Prickle2	Prox1	Prss40	Psd3	Ptchd3	R3hdm4	Rabl2
Rad51ap2	Rbm45	Rem2	Rfx5	Rims3	Rltpr	Rnase1	Rnase2b
Rpusd4	Rsph1	Rufy2	Rusc2	Samt3	Sass6	Scgb2b26	Scp2d1
Scyl1	Sec14l1	Selenbp1	Senp7	Serpinb9g	Shcbp1	Shisa3	Slc12a9
Slc25a38	Smap1	Smtnl1	Snora52	Snora62	Snora68	Snrk	Snx21
Spem1	Spert	Spink12	Sprr2i	Sprr2j-ps	Sprr2k	Ssh3	Ssscal
Stfa1	Stk32c	Stx7	Sult1d1	Supt20	Tapbpl	Tas2r106	Tas2r107

Tas2r114	Tex19.1	Tex36	Thap11	Tm4sf5	Tmem225	Tmem25	Tmem256
Tmem87b	Tmem98	Tnk1	Tox2	Trappc3l	Trim54	Trip13	Trip6
Trnp1	Tsnaxip1	Tspan4	Ttc23	Ttc36	Ttc38	Ttc7b	Ubac2
Ubap2	Ubqln3	Ubqlnl	Ufsp1	Ugt2b34	Uxs1	Vmn1r222	Vmn1r223
Vmn1r76	Vmn2r3	Vmn2r67	Vmn2r82	Vmn2r93	Vmn2r94	Vmo1	Vsnl1
Zc4h2	Zdhhc11	Zdhhc18	Zdhhc7	Zeb2	Zeb2os	Zfhx2	Zfhx2os
Zfp142	Zfp35	Zfp386	Zfp397	Zfp618	Zfyve28	Zkscan2	Zxda
Zxdb							
