# Supplementary material for: p.E95K mutation in Indian hedgehog causing brachydactyly type A1 impairs IHH/Gli1 downstream transcriptional regulation
Source: BMC Genet. 2019 Jan 16;20:10. doi: 10.1186/s12863-018-0697-5 (PMC6335781; doi:10.1186/s12863-018-0697-5)
Supplement: Supplementary file 8 — Pathway Analysis of Predicted Gli1 target genes in MT. Top 25 pathway analysis of predicted Gli1 target genes in MT. (PDF 1633 kb) [file 12863_2018_697_MOESM8_ESM.pdf]

reactome

Pathway Analysis Report

This report contains the pathway analysis results for the submitted sample ''. Analysis was performed against Reactome version 66 on 22/10/2018 using any resource identifiers for the mapping. The web link to these results is:

https://reactome.org/PathwayBrowser/#/ANALYSIS=MjAxODEwMjIxMjI1MzNfMTMzNDI%3D

Please keep in mind that analysis results are temporarily stored on our server. The storage period depends on usage of the service but is at least 7 days. As a result, please note that this URL is only valid for a limited time period and it might have expired.

Table of Contents

1. Introduction
2. Properties
3. Genome-wide overview
4. Most significant pathways
5. Pathway details
6. Identifiers found
7. Identifiers not found

1. Introduction

Reactome is a curated database of pathways and reactions in human biology. Reactions can be considered as pathway 'steps'. Reactome defines a 'reaction' as any event in biology that changes the state of a biological molecule. Binding, activation, translocation, degradation and classical biochemical events involving a catalyst are all reactions. Information in the database is authored by expert biologists, entered and maintained by Reactome’s team of curators and editorial staff. Reactome content frequently cross-references other resources e.g. NCBI, Ensembl, UniProt, KEGG (Gene and Compound), ChEBI, PubMed and GO. Orthologous reactions inferred from annotation for Homo sapiens are available for 17 non-human species including mouse, rat, chicken, puffer fish, worm, fly, yeast, rice, and Arabidopsis. Pathways are represented by simple diagrams following an SBGN-like format.

Reactome's annotated data describe reactions possible if all annotated proteins and small molecules were present and active simultaneously in a cell. By overlaying an experimental dataset on these annotations, a user can perform a pathway over-representation analysis. By overlaying quantitative expression data or time series, a user can visualize the extent of change in affected pathways and its progression. A binomial test is used to calculate the probability shown for each result, and the p-values are corrected for the multiple testing (Benjamini–Hochberg procedure) that arises from evaluating the submitted list of identifiers against every pathway.

To learn more about our Pathway Analysis, please have a look at our relevant publications:

Fabregat A, Sidiropoulos K, Garapati P, Gillespie M, Hausmann K, Haw R, … D’Eustachio P (2016). The reactome pathway knowledgebase. Nucleic Acids Research, 44(D1), D481–D487. https://doi.org/10.1093/nar/gkv1351.

Fabregat A, Sidiropoulos K, Viteri G, Forner O, Marin-Garcia P, Arnau V, … Hermjakob H (2017). Reactome pathway analysis: a high-performance in-memory approach. BMC Bioinformatics, 18.

2. Properties

- This is an **overrepresentation** analysis: A statistical (hypergeometric distribution) test that determines whether certain Reactome pathways are over-represented (enriched) in the submitted data. It answers the question 'Does my list contain more proteins for pathway X than would be expected by chance?' This test produces a probability score, which is corrected for false discovery rate using the Benjamani-Hochberg method.
- 51 out of 109 identifiers in the sample were found in Reactome, where 231 pathways were hit by at least one of them.
- All non-human identifiers have been converted to their human equivalent.
- This report is filtered to show only results and pathway diagrams for Homo sapiens.
- The unique ID for this analysis (token) is MjAxODEwMjIxMjI1MzNfMTMzNDI%3D. This ID is valid for at least 7 days in Reactome's server. Use it to access Reactome services with your data.

3. Genome-wide overview

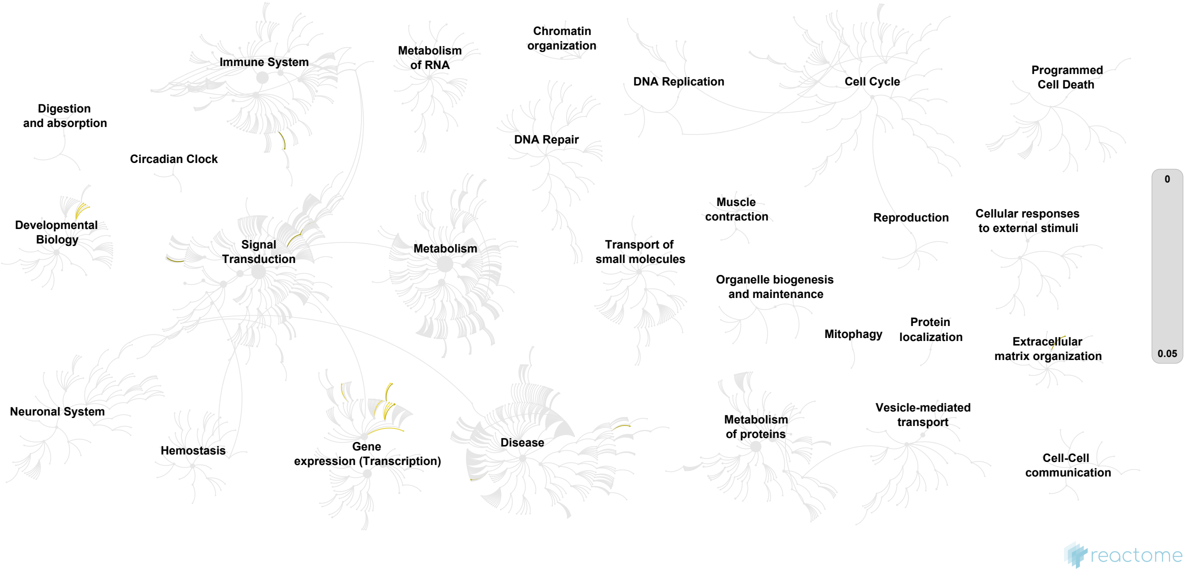

This figure shows a genome-wide overview of the results of your pathway analysis. Reactome pathways are arranged in a hierarchy. The center of each of the circular "bursts" is the root of one top-level pathway, for example "DNA Repair". Each step away from the center represents the next level lower in the pathway hierarchy. The color code denotes over-representation of that pathway in your input dataset. Light grey signifies pathways which are not significantly over-represented.

4. Top 25 pathways

Pathway name	Entities				Reactions	
	found	ratio	p-value	FDR*	found	ratio
RUNX2 regulates genes involved in differentiation of myeloid cells	2 / 6	4.29e-04	0.001	0.192	2 / 2	1.70e-04
RUNX2 regulates chondrocyte maturation	2 / 7	5.01e-04	0.002	0.192	4 / 4	3.39e-04
RUNX1 regulates transcription of genes involved in differentiation of myeloid cells	2 / 11	7.87e-04	0.004	0.307	1 / 7	5.94e-04
RUNX2 regulates genes involved in cell migration	2 / 14	0.001	0.006	0.318	7 / 7	5.94e-04
CRMPs in Sema3A signaling	2 / 18	0.001	0.01	0.318	5 / 5	4.24e-04
YAP1- and WWTR1 (TAZ)-stimulated gene expression	2 / 18	0.001	0.01	0.318	1 / 9	7.63e-04
SEMA3A-Plexin repulsion signaling by inhibiting Integrin adhesion	2 / 19	0.001	0.011	0.318	5 / 8	6.79e-04
Sema3A PAK dependent Axon repulsion	2 / 19	0.001	0.011	0.318	3 / 6	5.09e-04
TP53 Regulates Transcription of Genes Involved in G1 Cell Cycle Arrest	2 / 20	0.001	0.012	0.318	2 / 17	0.001
Regulation of RUNX2 expression and activity	3 / 80	0.006	0.029	0.527	32 / 32	0.003
Defective ALG14 causes congenital myasthenic syndrome (ALG14-CMS)	1 / 4	2.86e-04	0.033	0.527	1 / 1	8.48e-05
Synthesis and processing of ENV and VPU	1 / 4	2.86e-04	0.033	0.527	1 / 10	8.48e-04
RUNX2 regulates osteoblast differentiation	2 / 34	0.002	0.033	0.527	21 / 24	0.002
Activation of Matrix Metalloproteinases	2 / 35	0.003	0.035	0.527	5 / 27	0.002
Expression and Processing of Neurotrophins	1 / 5	3.58e-04	0.041	0.527	1 / 5	4.24e-04
NGF processing	1 / 5	3.58e-04	0.041	0.527	1 / 5	4.24e-04
Muscarinic acetylcholine receptors	1 / 6	4.29e-04	0.049	0.527	1 / 2	1.70e-04
Alternative complement activation	1 / 6	4.29e-04	0.049	0.527	2 / 9	7.63e-04
RUNX2 regulates bone development	2 / 43	0.003	0.05	0.527	29 / 32	0.003
Elastic fibre formation	2 / 46	0.003	0.056	0.527	7 / 17	0.001
Intestinal saccharidase deficiencies	1 / 8	5.72e-04	0.064	0.527	4 / 5	4.24e-04
Carboxyterminal post-translational modifications of tubulin	2 / 52	0.004	0.07	0.527	2 / 6	5.09e-04

Pathway name	Entities				Reactions	
	found	ratio	p-value	FDR*	found	ratio
Removal of aminoterminal propeptides from gamma-carboxylated proteins	1 / 10	7.15e-04	0.08	0.527	9 / 9	7.63e-04
Neurotransmitter uptake and metabolism In glial cells	1 / 10	7.15e-04	0.08	0.527	1 / 3	2.54e-04
Astrocytic Glutamate-Glutamine Uptake And Metabolism	1 / 10	7.15e-04	0.08	0.527	1 / 3	2.54e-04

* False Discovery Rate

5. Pathway details

1. RUNX2 regulates genes involved in differentiation of myeloid cells (R-HSA-8941333)

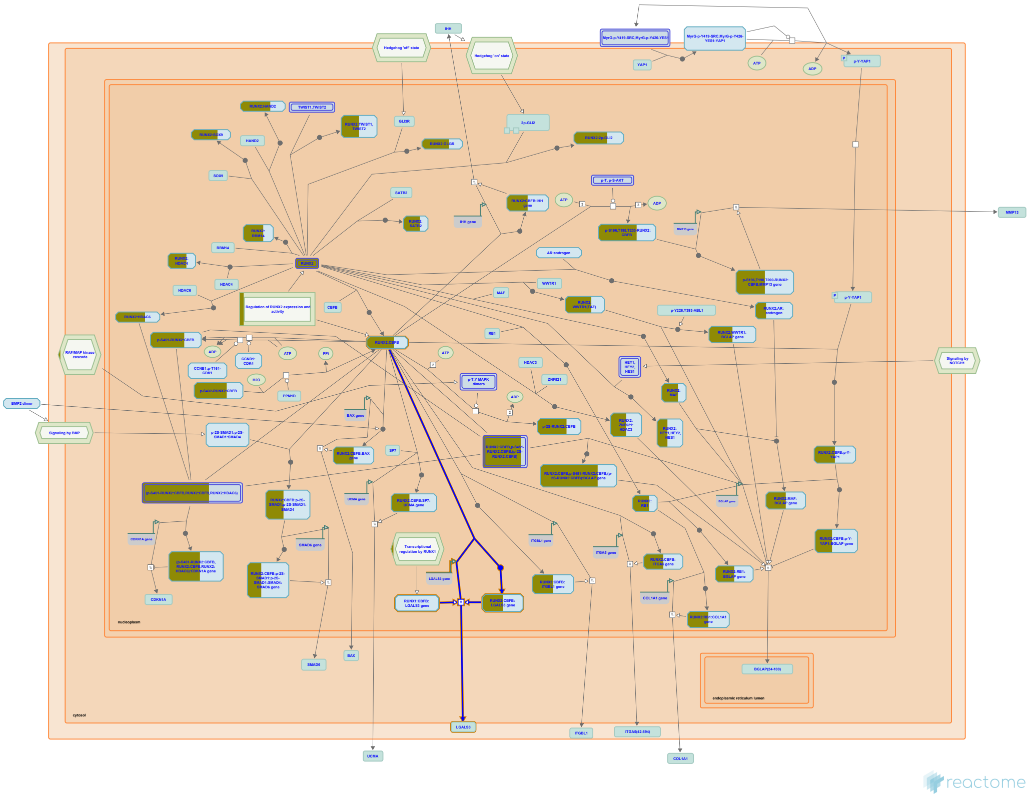

Both RUNX2 and RUNX1 can stimulate transcription of the LGALS3 gene, encoding Galectin-3 (Vladimirova et al. 2008, Zhang et al. 2009). Galectin 3 is expressed in myeloid progenitors and its levels increase during the maturation process (Le Marer 2000). Galectin 3 is highly expressed in pituitary tumors and glioma (Vladimirova et al. 2008, Zhang et al. 2009).

References

Zhang HY, Jin L, Stilling GA, Ruebel KH, Coonse K, Tanizaki Y, ... Lloyd RV (2009). RUNX1 and RUNX2 upregulate Galectin-3 expression in human pituitary tumors. Endocrine, 35, 101-11.

Vladimirova V, Waha A, Lückerath K, Pesheva P & Probstmeier R (2008). Runx2 is expressed in human glioma cells and mediates the expression of galectin-3. J. Neurosci. Res., 86, 2450-61.

Le Marer N (2000). GALECTIN-3 expression in differentiating human myeloid cells. Cell Biol. Int., 24, 245-51.

Edit history

Date	Action	Author
2016-09-30	Authored	Orlic-Milacic M
2016-09-30	Created	Orlic-Milacic M
2017-08-04	Reviewed	Ducy P

Date	Action	Author
2017-08-09	Edited	Orlic-Milacic M
2018-08-30	Modified	Croft D

Elements found in this pathway

Input	UniProt Id	Input	UniProt Id	Input	UniProt Id
Runx2	Q13950-2, Q13950-1				

2. RUNX2 regulates chondrocyte maturation (R-HSA-8941284)

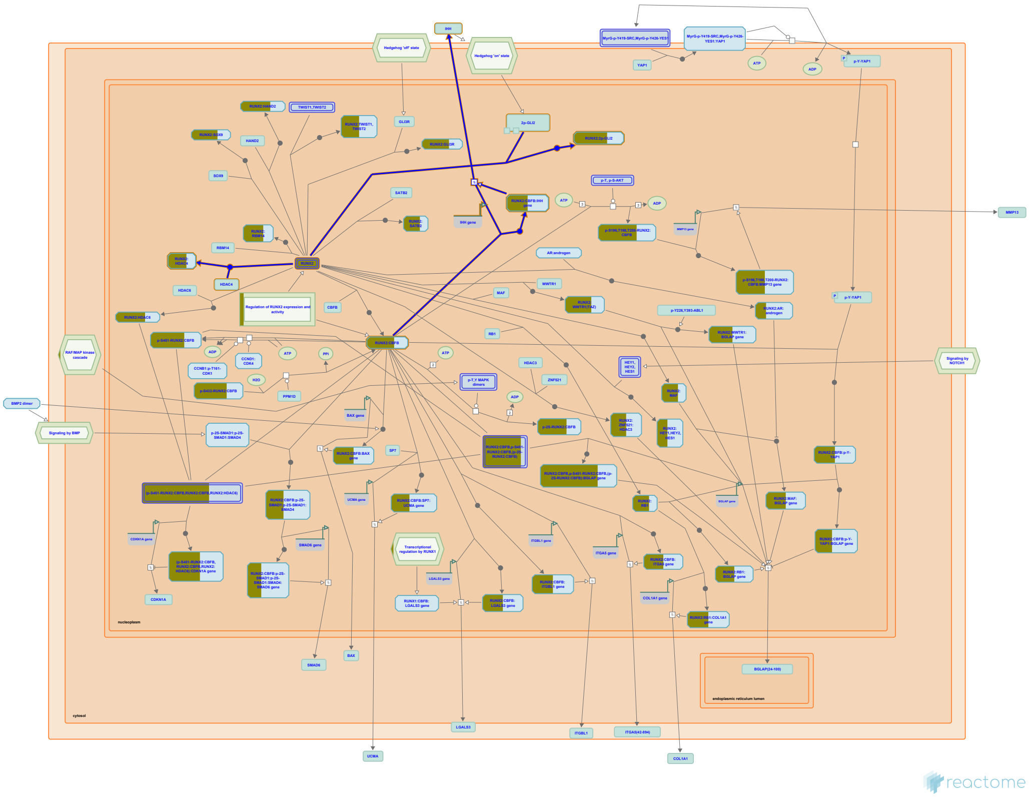

In addition to regulating osteoblast differentiation, RUNX2 regulates skeletal development by regulating maturation of chondrocytes (Takeda et al. 2001). Chondrocyte maturation happens during the process of endochondral ossification. Expression of the parathyroid hormone receptor (PTHR1) and Indian hedgehog (IHH) are hallmarks of chondrocyte maturation. Mice that are double knockouts for Runx2 and Runx3 show a complete absence of chondrocyte maturation and, hence, aberrant limb growth. Based on mouse studies, RUNX2 directly regulates transcription of the IHH gene. RUNX2 binding sites in the IHH gene promoter are conserved in humans (Yoshida et al. 2004). Also based on mouse studies, RUNX2 positively regulates transcription of NELL1 (neural EGFL-like 1), a key functional mediator of chondrogenesis, but direct binding of RUNX2 to the NELL1 gene locus has not been demonstrated (Li et al. 2017). Runx2 binding sites exist in the enhancer of the mouse Col10a1 gene, encoding type X collagen, a marker of hypertrophic chondrocytes, which is critical for endochondral bone formation. While Runx2 binding is required, it is not sufficient to trigger Col10a1 transcription (Gu et al. 2014).

References

Yoshida CA, Yamamoto H, Fujita T, Furuichi T, Ito K, Inoue K, ... Komori T (2004). Runx2 and Runx3 are essential for chondrocyte maturation, and Runx2 regulates limb growth through induction of Indian hedgehog. Genes Dev., 18, 952-63.

Li C, Jiang J, Zheng Z, Lee KS, Zhou Y, Chen E, ... Soo C (2017). Neural EGFL-Like 1 Is a Downstream Regulator of Runt-Related Transcription Factor 2 in Chondrogenic Differentiation and Maturation. Am. J. Pathol., 187, 963-972.

Gu J, Lu Y, Li F, Qiao L, Wang Q, Li N, ... Zheng Q (2014). Identification and characterization of the novel Col10a1 regulatory mechanism during chondrocyte hypertrophic differentiation. Cell Death Dis, 5, e1469.

Takeda S, Bonnamy JP, Owen MJ, Ducy P & Karsenty G (2001). Continuous expression of Cbfa1 in nonhypertrophic chondrocytes uncovers its ability to induce hypertrophic chondrocyte differentiation and partially rescues Cbfa1-deficient mice. Genes Dev., 15, 467-81.

Edit history

Date	Action	Author
2016-09-30	Authored	Orlic-Milacic M
2016-09-30	Created	Orlic-Milacic M
2017-08-04	Reviewed	Ducy P
2017-08-09	Edited	Orlic-Milacic M
2018-08-30	Modified	Croft D

Elements found in this pathway

Input	UniProt Id	Input	UniProt Id	Input	UniProt Id
Runx2	Q13950-2, Q13950-1				

3. RUNX1 regulates transcription of genes involved in differentiation of myeloid cells (R-HSA-8939246)

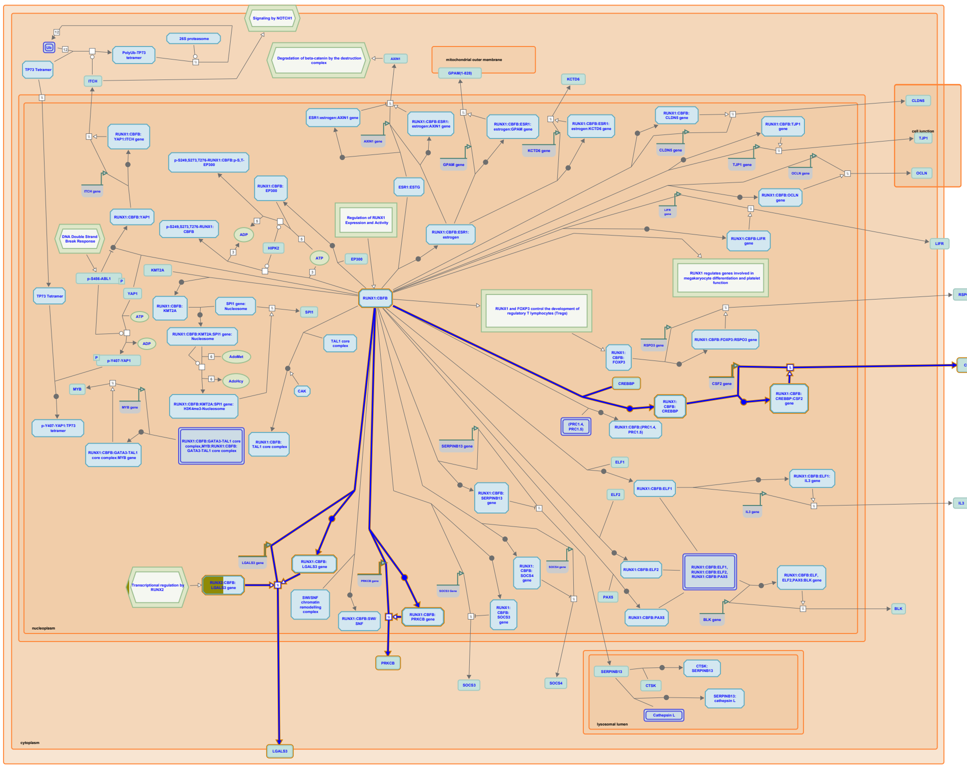

The RUNX1:CBFB complex regulates expression of genes involved in differentiation of myeloid progenitors which can commit to hematopoietic lineages that lead to generation of platelets, erythrocytes, leukocytes or monocytes.

The RUNX1:CBFB complex recruits histone acetyltransferase CREBBP (CBP) to the promoter of the CSF2 gene, encoding Granulocyte-macrophage colony stimulating factor (GM-CSF), thus inducing GM-CSF expression (Oakford et al. 2010). GM-CSF induces growth, differentiation and survival of macrophages, granulocytes, erythrocytes and megakaryocytes from myeloid progenitors (Barreda et al. 2004).

The RUNX1:CBFB complex directly stimulates transcription of the LGALS3 gene, encoding galectin-3 (Zhang et al. 2009). Galectin-3 is expressed in myeloid progenitors and its levels increase during the maturation process (Le Marer 2000).

The PRKCB gene, encoding protein kinase C-beta, which regulates apoptosis of myeloid cells, is directly transactivated by the RUNX1:CBFB complex (Hu et al. 2004).

References

Oakford PC, James SR, Qadi A, West AC, Ray SN, Bert AG, ... Holloway AF (2010). Transcriptional and epigenetic regulation of the GM-CSF promoter by RUNX1. Leuk. Res., 34, 1203-13.

Barreda DR, Hanington PC & Belosevic M (2004). Regulation of myeloid development and function by colony stimulating factors. Dev. Comp. Immunol., 28, 509-54.

Zhang HY, Jin L, Stilling GA, Ruebel KH, Coonse K, Tanizaki Y, ... Lloyd RV (2009). RUNX1 and RUNX2 upregulate Galectin-3 expression in human pituitary tumors. Endocrine, 35, 101-11.

Le Marer N (2000). GALECTIN-3 expression in differentiating human myeloid cells. Cell Biol. Int., 24, 245-51.

Hug BA, Ahmed N, Robbins JA & Lazar MA (2004). A chromatin immunoprecipitation screen reveals protein kinase Cbeta as a direct RUNX1 target gene. J. Biol. Chem., 279, 825-30.

Edit history

Date	Action	Author
2016-09-14	Authored	Orlic-Milacic M
2016-09-17	Created	Orlic-Milacic M
2016-12-20	Reviewed	Ito Y, Chuang LS
2017-05-09	Edited	Orlic-Milacic M
2018-08-23	Modified	Schmidt EE

Elements found in this pathway

Input	UniProt Id	Input	UniProt Id	Input	UniProt Id
Runx2	Q13950-2, Q13950-1				

4. RUNX2 regulates genes involved in cell migration (R-HSA-8941332)

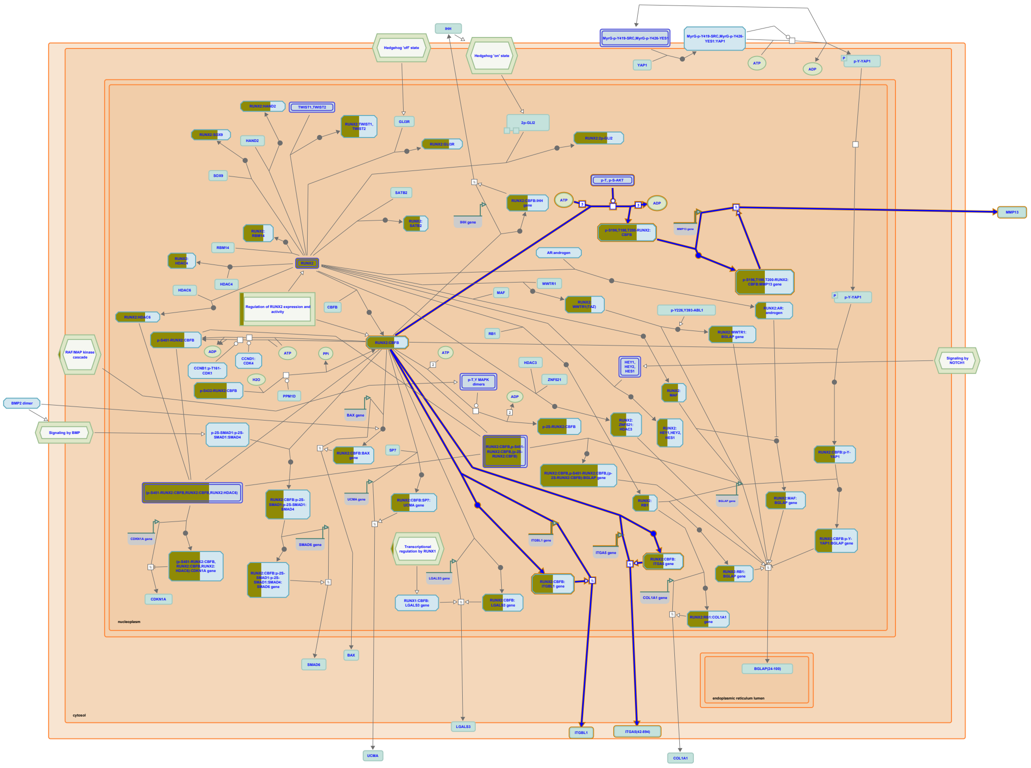

RUNX2 regulates expression of several genes implicated in cell migration during normal development and bone metastasis of breast cancer cells.

RUNX2 stimulates transcription of the ITGA5 gene, encoding Integrin alpha 5. Integrin alpha-5 promotes adhesion of breast cancer cells to the bone, thus facilitating formation of bone metastases (Li et al. 2016). ITGA5 is implicated in migration of human dental pulp stem cells (Xu et al. 2015). In zebrafish, Integrin alpha-5 coordinates cell migration during development of sensory organs (Bhat et al. 2011). During mouse retinal angiogenesis, Integrin alpha-5 regulates migration of endothelial cells (Stenzel et al. 2011).

The ITGBL1 gene encodes Integrin beta like protein 1, which is implicated in regulation of TGF-beta signaling and RUNX2-induced bone metastasis of breast cancer (Li et al. 2015).

RUNX2 mediated transcription of the MMP13 gene, encoding Colagenase 3 (Matrix metalloproteinase 13), is stimulated by AKT mediated phosphorylation of RUNX2 and is implicated in invasiveness of breast cancer cells (Pande et al. 2013). MMP13 is involved in migration of innate immune system cells in response to injury (Zhang et al. 2008) and in remodelling of skeletal tissues (Ortega et al. 2003).

References

Li XQ, Lu JT, Tan CC, Wang QS & Feng YM (2016). RUNX2 promotes breast cancer bone metastasis by increasing integrin 5-mediated colonization. Cancer Lett., 380, 78-86.

Li XQ, Du X, Li DM, Kong PZ, Sun Y, Liu PF, ... Feng YM (2015). ITGBL1 Is a Runx2 Transcriptional Target and Promotes Breast Cancer Bone Metastasis by Activating the TGF Signaling Pathway. Cancer Res., 75, 3302-13.

Pande S, Browne G, Padmanabhan S, Zaidi SK, Lian JB, van Wijnen AJ, ... Stein GS (2013). Oncogenic cooperation between PI3K/Akt signaling and transcription factor Runx2 promotes the invasive properties of metastatic breast cancer cells. J. Cell. Physiol., 228, 1784-92.

Xu S, Cui L, Ma D, Sun W & Wu B (2015). Effect of ITGA5 down-regulation on the migration capacity of human dental pulp stem cells. Int J Clin Exp Pathol, 8, 14425-32.

Bhat N & Riley BB (2011). Integrin-5 coordinates assembly of posterior cranial placodes in zebrafish and enhances Fgf-dependent regulation of otic/epibranchial cells. PLoS ONE, 6, e27778.

Edit history

Date	Action	Author
2016-09-30	Authored	Orlic-Milacic M
2016-09-30	Created	Orlic-Milacic M
2017-08-04	Reviewed	Ducy P
2017-08-09	Edited	Orlic-Milacic M
2018-08-30	Modified	Croft D

Elements found in this pathway

Input	UniProt Id	Input	UniProt Id	Input	UniProt Id
Runx2	Q13950-2, Q13950-1				

5. CRMPs in Sema3A signaling (R-HSA-399956)

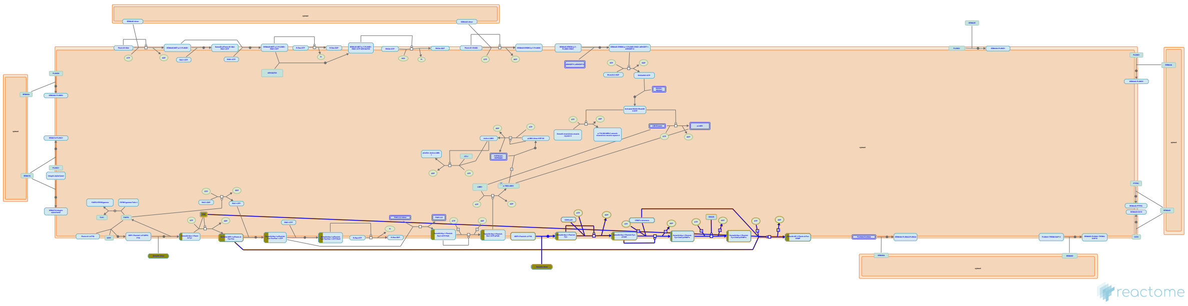

CRMPs are a small family of plexinA-interacting cytosolic phosphoproteins identified as mediators of Sema3A signaling and neuronal differentiation. After Sema3A activation Plexin-A bound CRMP's undergo phosphorylation by Cdk5, GSK3beta and Fes kinases. Phosphorylation of CRMPs by these kinases blocks the ability of CRMP to bind to tubulin dimers, subsequently induces depolymerization of F-actin, and ultimately leads to growth cone collapse.

References

Schmidt EF & Strittmatter SM (2007). The CRMP family of proteins and their role in Sema3A signaling. Adv Exp Med Biol, 600, 1-11.

Edit history

Date	Action	Author
2009-03-23	Edited	Garapati P V
2009-03-23	Authored	Garapati P V
2009-03-23	Created	Garapati P V
2009-09-02	Reviewed	Kumanogoh A, Kikutani H
2018-08-23	Modified	Schmidt EE

Elements found in this pathway

Input	UniProt Id	Input	UniProt Id	Input	UniProt Id
Fes	P07332	Sema3a	Q14563		

6. YAP1- and WWTR1 (TAZ)-stimulated gene expression (R-HSA-2032785)

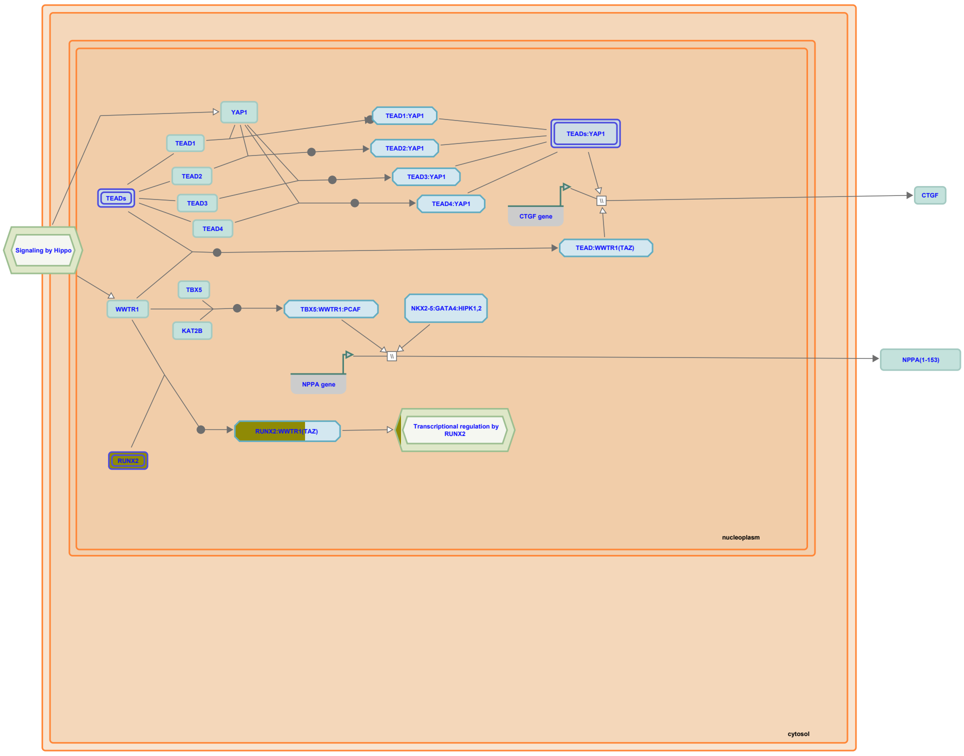

Cellular compartments: cytosol, nucleoplasm.

YAP1 and WWTR1 (TAZ) are transcriptional co-activators, both homologues of the Drosophila Yorkie protein. They both interact with members of the TEAD family of transcription factors, and WWTR1 interacts as well with TBX5 and RUNX2, to promote gene expression. Their transcriptional targets include genes critical to regulation of cell proliferation and apoptosis. Their subcellular location is regulated by the Hippo signaling cascade: phosphorylation mediated by this cascade leads to the cytosolic sequestration of both proteins (Murakami et al. 2005; Oh and Irvine 2010).

References

Murakami M, Nakagawa M, Olson EN & Nakagawa O (2005). A WW domain protein TAZ is a critical coactivator for TBX5, a transcription factor implicated in Holt-Oram syndrome. Proc Natl Acad Sci U S A, 102, 18034-9.

Oh H & Irvine KD (2010). Yorkie: the final destination of Hippo signaling. Trends Cell Biol, 20, 410-7.

Edit history

Date	Action	Author
2012-01-07	Edited	D'Eustachio P
2012-01-07	Created	D'Eustachio P
2012-02-03	Reviewed	Sudol M
2012-02-03	Authored	D'Eustachio P
2018-08-23	Modified	Schmidt EE

Elements found in this pathway

Input	UniProt Id	Input	UniProt Id	Input	UniProt Id
Runx2	Q13950-2, Q13950-1				

7. SEMA3A-Plexin repulsion signaling by inhibiting Integrin adhesion (R-HSA-399955)

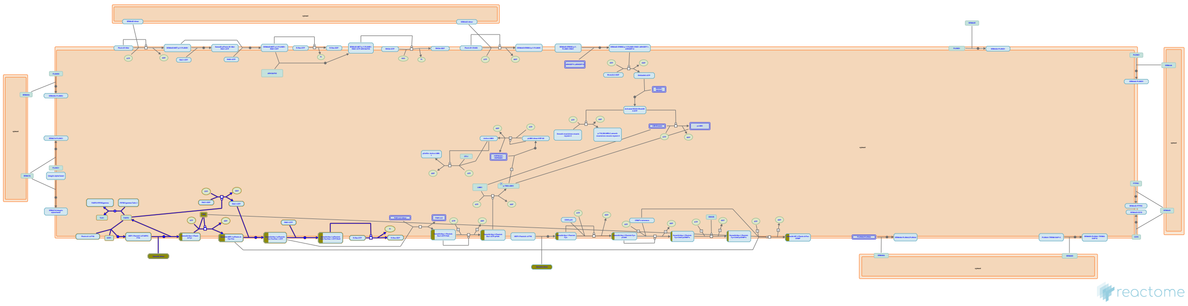

Sema3A, a prototypical semaphorin, acts as a chemorepellent or a chemoattractant for axons by activating a receptor complex comprising neuropilin-1 as the ligand-binding subunit and plexin-A1 as the signal-transducing subunit. Sema3A inhibits cell migration by inhibiting integrin ligand-binding activity.

References

Neufeld G & Kessler O (2008). The semaphorins: versatile regulators of tumour progression and tumour angiogenesis. Nat Rev Cancer, 8, 632-45.

Toyofuku T, Yoshida J, Sugimoto T, Zhang H, Kumanogoh A, Hori M & Kikutani H (2005). FARP2 triggers signals for Sema3A-mediated axonal repulsion. Nat Neurosci, 8, 1712-9.

Zhou Y, Gunput RA & Pasterkamp RJ (2008). Semaphorin signaling: progress made and promises ahead. Trends Biochem Sci, 33, 161-70.

Edit history

Date	Action	Author
2009-03-23	Edited	Garapati P V
2009-03-23	Authored	Garapati P V
2009-03-23	Created	Garapati P V
2009-09-02	Reviewed	Kumanogoh A, Kikutani H
2018-08-23	Modified	Schmidt EE

Elements found in this pathway

Input	UniProt Id	Input	UniProt Id	Input	UniProt Id
Fes	P07332	Sema3a	Q14563		

8. Sema3A PAK dependent Axon repulsion (R-HSA-399954)

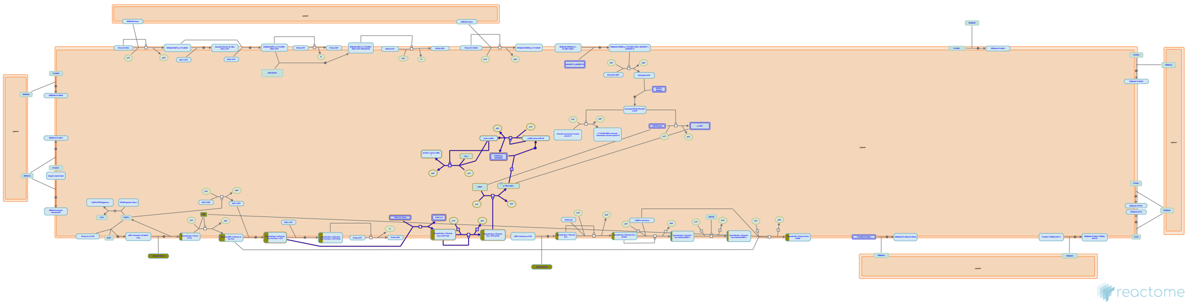

Activated Rac1 bound to plexin-A might modulate actin dynamics through the sequential phosphorylation and activation of PAK, LIMK1 and cofilin.

References

Pasterkamp RJ & Kolodkin AL (2003). Semaphorin junction: making tracks toward neural connectivity. Curr Opin Neurobiol, 13, 79-89.

Zhou Y, Gunput RA & Pasterkamp RJ (2008). Semaphorin signaling: progress made and promises ahead. Trends Biochem Sci, 33, 161-70.

Whitford KL & Ghosh A (2001). Plexin signaling via off-track and rho family GTPases. Neuron, 32, 1-3.

Aizawa H, Wakatsuki S, Ishii A, Moriyama K, Sasaki Y, Ohashi K, ... Yahara I (2001). Phosphorylation of cofilin by LIM-kinase is necessary for semaphorin 3A-induced growth cone collapse. Nat Neurosci, 4, 367-73.

Edit history

Date	Action	Author
2009-03-23	Edited	Garapati P V
2009-03-23	Authored	Garapati P V
2009-03-23	Created	Garapati P V
2009-09-02	Reviewed	Kumanogoh A, Kikutani H
2018-08-23	Modified	Schmidt EE

Elements found in this pathway

Input	UniProt Id	Input	UniProt Id	Input	UniProt Id
Fes	P07332	Sema3a	Q14563		

9. TP53 Regulates Transcription of Genes Involved in G1 Cell Cycle Arrest (R-HSA-6804116)

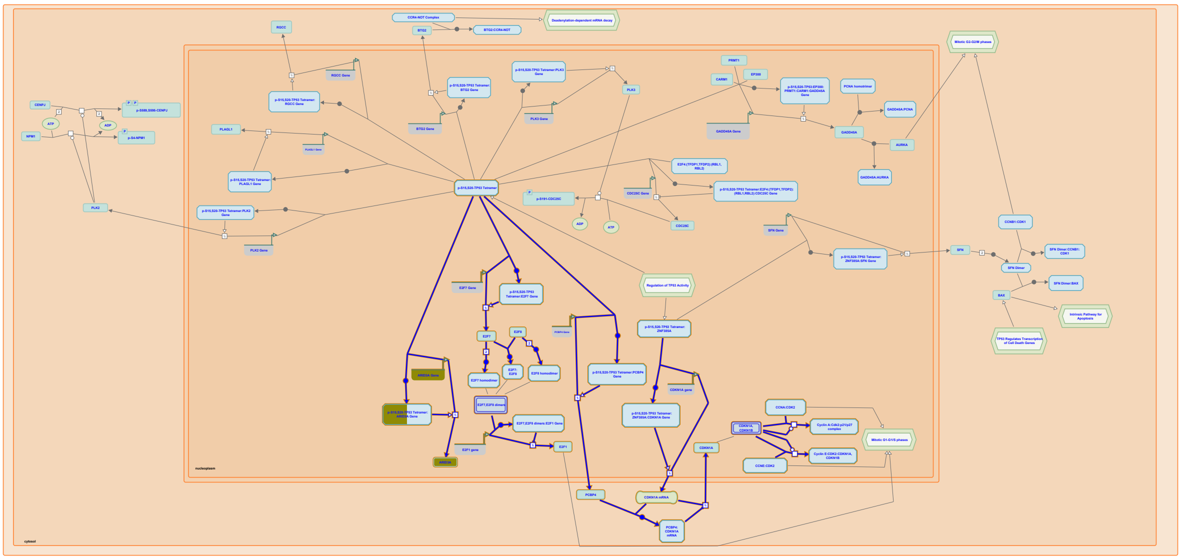

The most prominent TP53 target involved in G1 arrest is the inhibitor of cyclin-dependent kinases CDKN1A (p21). CDKN1A is one of the earliest genes induced by TP53 (El-Deiry et al. 1993). CDKN1A binds and inactivates CDK2 in complex with cyclin A (CCNA) or E (CCNE), thus preventing G1/S transition (Harper et al. 1993). Considering its impact on the cell cycle outcome, CDKN1A expression levels are tightly regulated. For instance, under prolonged stress, TP53 can induce the transcription of an RNA binding protein PCBP4, which can bind and destabilize CDKN1A mRNA, thus alleviating G1 arrest and directing the affected cell towards G2 arrest and, possibly, apoptosis (Zhu and Chen 2000, Scoumanne et al. 2011). Expression of E2F7 is directly induced by TP53. E2F7 contributes to G1 cell cycle arrest by repressing transcription of E2F1, a transcription factor that promotes expression of many genes needed for G1/S transition (Aksoy et al. 2012, Carvajal et al. 2012). ARID3A is a direct transcriptional target of TP53 (Ma et al. 2003) that may promote G1 arrest by cooperating with TP53 in induction of CDKN1A transcription (Lestari et al. 2012). However, ARID3A may also promote G1/S transition by stimulating transcriptional activity of E2F1 (Suzuki et al. 1998, Peeper et al. 2002).

TP53 has co-factors that are key determinants of transcriptional selectivity within the p53 network. For instance, the zinc finger transcription factor ZNF385A (HZF) is a direct transcriptional target of TP53 that can form a complex with TP53 and facilitate TP53-mediated induction of CDKN1A, strongly favouring cell cycle arrest over apoptosis (Das et al. 2007).

References

el-Deiry WS, Tokino T, Velculescu VE, Levy DB, Parsons R, Trent JM, ... Vogelstein B (1993). WAF1, a potential mediator of p53 tumor suppression. Cell, 75, 817-25.

Harper JW, Adami GR, Wei N, Keyomarsi K & Elledge SJ (1993). The p21 Cdk-interacting protein Cip1 is a potent inhibitor of G1 cyclin-dependent kinases. Cell, 75, 805-16.

Zhu J & Chen X (2000). MCG10, a novel p53 target gene that encodes a KH domain RNA-binding protein, is capable of inducing apoptosis and cell cycle arrest in G(2)-M. Mol. Cell. Biol., 20, 5602-18.

Scoumanne A, Cho SJ, Zhang J & Chen X (2011). The cyclin-dependent kinase inhibitor p21 is regulated by RNA-binding protein PCBP4 via mRNA stability. Nucleic Acids Res., 39, 213-24.

Aksoy O, Chicas A, Zeng T, Zhao Z, McCurrach M, Wang X & Lowe SW (2012). The atypical E2F family member E2F7 couples the p53 and RB pathways during cellular senescence. Genes Dev., 26, 1546-57.

Edit history

Date	Action	Author
2015-10-08	Created	Orlic-Milacic M
2015-10-14	Edited	Orlic-Milacic M
2015-10-14	Authored	Orlic-Milacic M
2016-02-04	Reviewed	Zaccara S, Inga A
2017-01-03	Revised	Orlic-Milacic M
2018-08-30	Modified	Croft D

Elements found in this pathway

Input	UniProt Id	Input	UniProt Id	Input	UniProt Id
Arid3a	Q99856				

Input	Ensembl Id	Input	Ensembl Id	Input	Ensembl Id
Arid3a	ENSG00000116017				

10. Regulation of RUNX2 expression and activity (R-HSA-8939902)

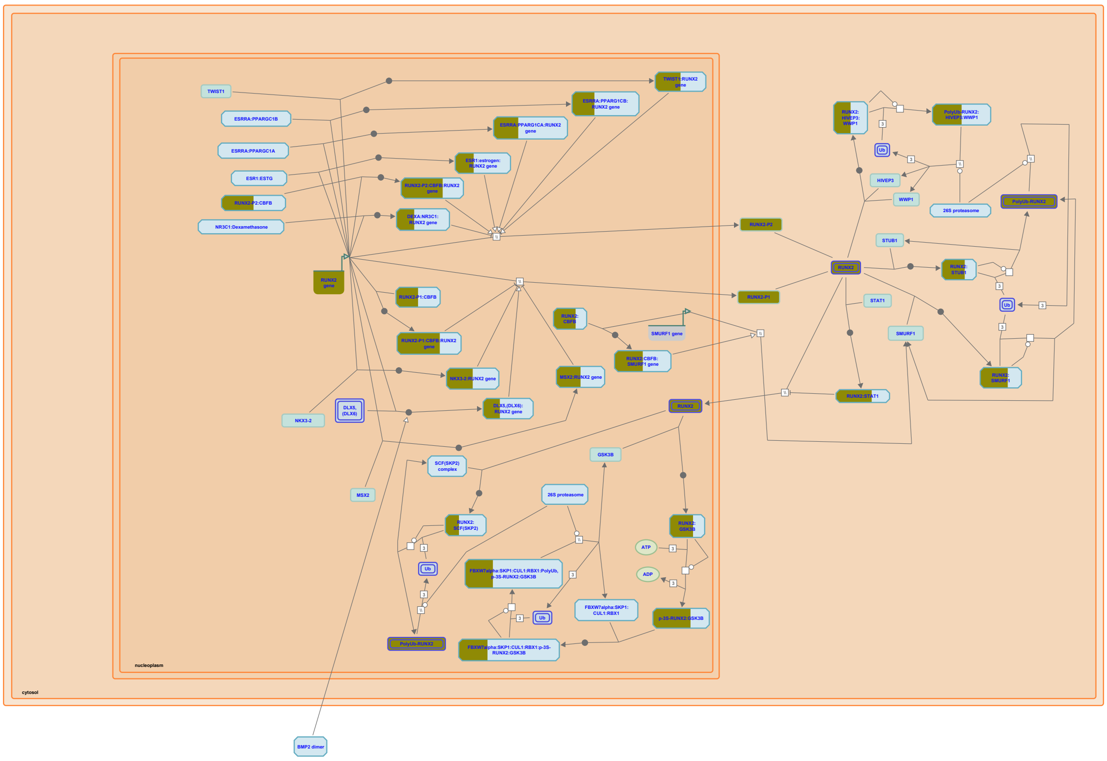

Cellular compartments: nucleoplasm.

Several transcription factors have been implicated in regulation of the RUNX2 gene transcription. Similar to the RUNX1 gene, the RUNX2 gene expression can be regulated from the proximal P2 promoter or the distal P1 promoter (reviewed in Li and Xiao 2007).

Activated estrogen receptor alpha (ESR1) binds estrogen response elements (EREs) in the P2 promoter and stimulates RUNX2 transcription (Kammerer et al. 2013). Estrogen-related receptor alpha (ERRA) binds EREs or estrogen-related response elements (ERREs) in the P2 promoter of RUNX2. When ERRA is bound to its co-factor PPARG1CA (PGC1A), it stimulates RUNX2 transcription. When bound to its co-factor PPARG1CB (PGC1B), ERRA represses RUNX2 transcription (Kammerer et al. 2013).

TWIST1, a basic helix-loop-helix (bHLH) transcription factor, stimulates RUNX2 transcription by binding to the E1-box in the P2 promoter (Yang, Yang et al. 2011). TWIST proteins also interact with the DNA-binding domain of RUNX2 to modulate its activity during skeletogenesis (Bialek et al. 2004). Schnurri-3 (SHN3) is another protein that interacts with RUNX2 to decrease its availability in the nucleus and therefore its activity (Jones et al. 2006). In contrast, RUNX2 and SATB2 interact to enhance the expression of osteoblast-specific genes (Dobreva et al. 2006). Formation of the heterodimer with CBFB (CBF-beta) also enhances the transcriptional activity of RUNX2 (Kundu et al. 2002, Yoshida et al. 2002, Otto et al. 2002).

Transcription of RUNX2 from the proximal promoter is inhibited by binding of the glucocorticoid receptor (NR3C1) activated by dexamethasone (DEXA) to a glucocorticoid receptor response element (GRE), which is also present in the human promoter (Zhang et al. 2012).

NKX3-2 (BAPX1), required for embryonic development of the axial skeleton (Tribioli and Lufkin 1999), binds the distal (P1) promoter of the RUNX2 gene and inhibits its transcription (Lengner et al. 2005). RUNX2-P1 transcription is also autoinhibited by RUNX2-P1, which binds to RUNX2 response elements in the P1 promoter of RUNX2 (Drissi et al. 2000). In contrast, binding of RUNX2-P2 to the proximal P2 promoter autoactivates transcription of RUNX2-P2 (Ducy et al. 1999). Binding of a homeodomain transcription factor DLX5, and possibly DLX6, to the RUNX2 P1 promoter stimulates RUNX2 transcription (Robledo et al. 2002, Lee et al. 2005). The homeobox transcription factor MSX2 can bind to DLX5 sites in the promoter of RUNX2 and inhibit transcription of RUNX2-P1 (Lee et al. 2005).

Translocation of RUNX2 protein to the nucleus is inhibited by binding to non-activated STAT1 (Kim et al. 2003).

Several E3 ubiquitin ligases were shown to polyubiquitinate RUNX2, targeting it for proteasome-mediated degradation: FBXW7a (Kumar et al. 2015), STUB1 (CHIP) (Li et al. 2008), SMURF1 (Zhao et al. 2003, Yang et al. 2014), WWP1 (Jones et al. 2006), and SKP2 (Thacker et al. 2016).

References

Kammerer M, Gutzwiller S, Stauffer D, Delhon I, Seltenmeyer Y & Fournier B (2013). Estrogen Receptor (ER) and Estrogen Related Receptor (ERR) are both transcriptional regulators of the Runx2-I isoform. Mol. Cell. Endocrinol., 369, 150-60.

Yang DC, Yang MH, Tsai CC, Huang TF, Chen YH & Hung SC (2011). Hypoxia inhibits osteogenesis in human mesenchymal stem cells through direct regulation of RUNX2 by TWIST. PLoS ONE, 6, e23965.

Li YL & Xiao ZS (2007). Advances in Runx2 regulation and its isoforms. Med. Hypotheses, 68, 169-75.

Zhang YY, Li X, Qian SW, Guo L, Huang HY, He Q, ... Tang QQ (2012). Down-regulation of type I Runx2 mediated by dexamethasone is required for 3T3-L1 adipogenesis. Mol. Endocrinol., 26, 798-808.

Lengner CJ, Hassan MQ, Serra RW, Lepper C, van Wijnen AJ, Stein JL, ... Stein GS (2005). Nkx3.2-mediated repression of Runx2 promotes chondrogenic differentiation. J. Biol. Chem., 280, 15872-9.

Edit history

Date	Action	Author
2016-09-22	Authored	Orlic-Milacic M
2016-09-22	Created	Orlic-Milacic M
2017-08-04	Reviewed	Ducy P
2017-08-09	Edited	Orlic-Milacic M
2018-08-23	Modified	Schmidt EE

Elements found in this pathway

Input	UniProt Id	Input	UniProt Id	Input	UniProt Id
Runx2	Q13950-2, Q13950-1				

Input	Ensembl Id	Input	Ensembl Id	Input	Ensembl Id
Runx2	ENSG00000124813				

11. Defective ALG14 causes congenital myasthenic syndrome (ALG14-CMS) (R-HSA-5633231)

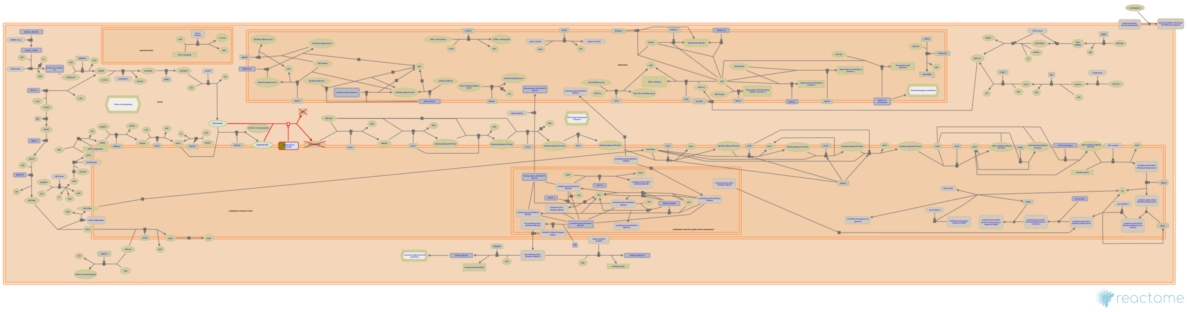

Diseases: congenital myasthenic syndrome.

UDP-N-acetylglucosamine transferase subunit ALG14 homolog (ALG14) forms a complex with ALG13 protein and is required for the addition of the second N-acetylglucosamine (GlcNAc) to the lipid linked oligosaccharide (LLO) intermediate (GlcNAcDOLDP) (Gao et al. 2005). Defects in ALG14 can cause congenital myasthenic syndrome (ALG14-CMS), which is due to a defect in neuromuscular signal transmission (Cossins et al. 2013). The most commonly affected muscles include proximal limb muscles. Mutations causing ALG14-CMS include p.P65L and p.R104* (Cossins et al. 2013).

References

Gao XD, Tachikawa H, Sato T, Jigami Y & Dean N (2005). Alg14 recruits Alg13 to the cytoplasmic face of the endoplasmic reticulum to form a novel bipartite UDP-N-acetylglucosamine transferase required for the second step of N-linked glycosylation. J Biol Chem, 280, 36254-62.

Cossins J, Belaya K, Hicks D, Salih MA, Finlayson S, Carboni N, ... Seidhamed MZ (2013). Congenital myasthenic syndromes due to mutations in ALG2 and ALG14. Brain, 136, 944-56.

Edit history

Date	Action	Author
2014-10-31	Edited	Jassal B
2014-10-31	Reviewed	Belaya K
2014-10-31	Authored	Jassal B
2014-10-31	Created	Jassal B
2016-11-08	Modified	Shorser S

Elements found in this pathway

Input	UniProt Id	Input	UniProt Id	Input	UniProt Id
Alg13	Q9NP73				

12. Synthesis and processing of ENV and VPU (R-HSA-171286)

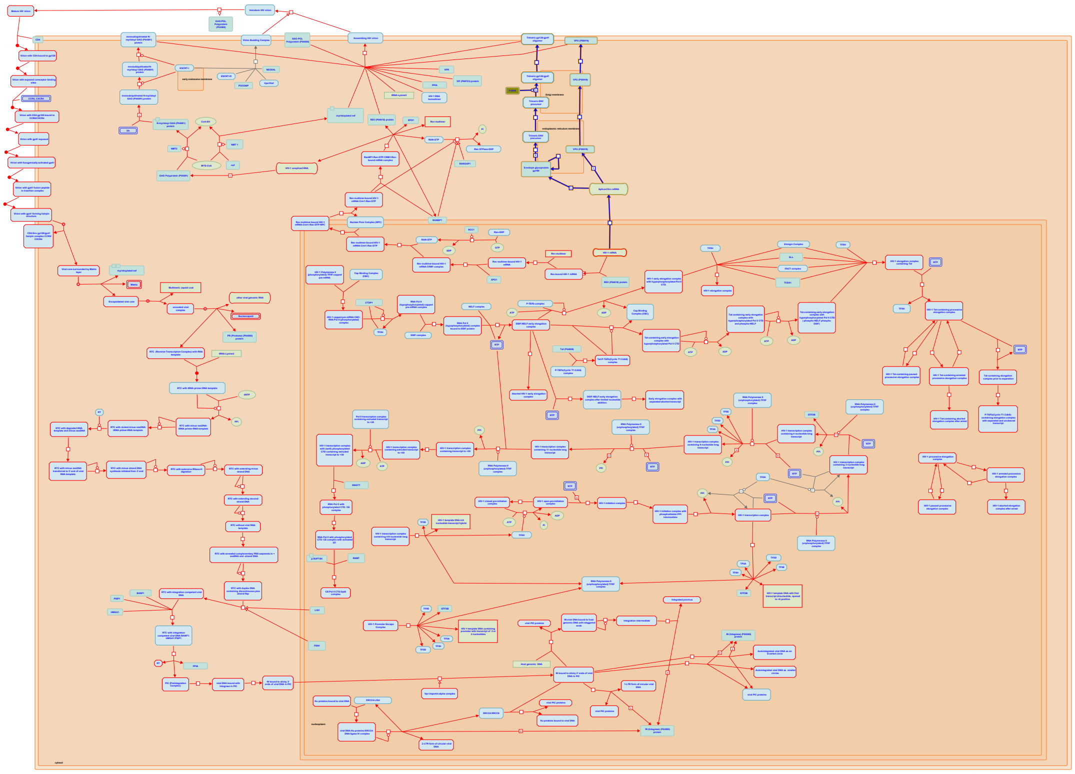

reactome

Diseases: Human immunodeficiency virus infectious disease.

The two viral membrane proteins, Env and the accessory protein Vpu, which are encoded by the same mRNA, are translated on the rough ER. All virion components need to traffic from their point of synthesis to sites of assembly on the plasma membrane. Env is an integral membrane protein. It is inserted cotranslationally into ER membranes and then travels through the cellular secretory pathway where it is glycosylated, assembled into trimeric complexes, processed into the gp41 and gp120 subunits by the cellular protease furin.

References

Sundquist WI & Kräusslich HG (2012). HIV-1 Assembly, Budding, and Maturation. Cold Spring Harb Perspect Med, 2, a006924.

Edit history

Date	Action	Author
2006-02-03	Created	Gopinathrao G
2013-01-30	Edited	Gillespie ME
2013-03-07	Authored	Gillespie ME
2013-05-21	Reviewed	Dube M
2014-08-29	Modified	Matthews L

Elements found in this pathway

Input	UniProt Id	Input	UniProt Id	Input	UniProt Id
Furin	P09958				

13. RUNX2 regulates osteoblast differentiation (R-HSA-8940973)

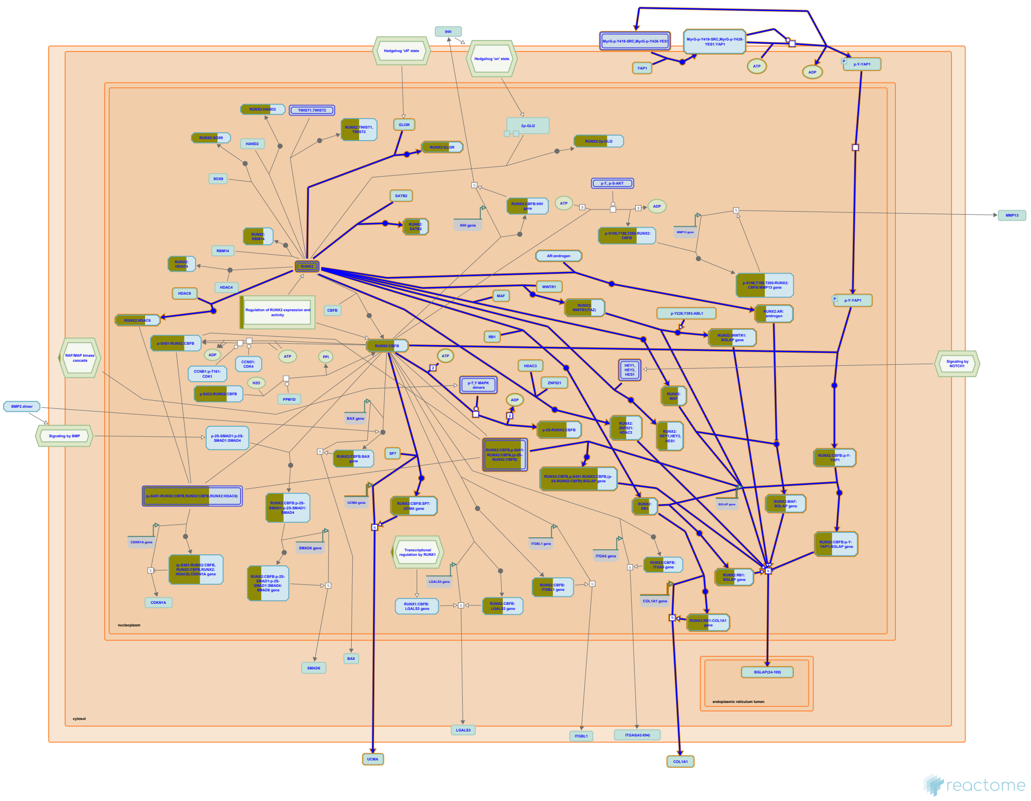

The complex of RUNX2 and CBFB regulates transcription of genes involved in differentiation of osteoblasts.

RUNX2 stimulates transcription of the BGLAP gene, encoding osteocalcin (Ducy and Karsenty 1995, Ducy et al. 1997). Binding of the RUNX2:CBFB complex to the BGLAP gene promoter is increased when RUNX2 is phosphorylated on serine residue S451 (Wee et al. 2002). Osteocalcin, a bone-derived hormone, is one of the most abundant non-collagenous proteins of the bone extracellular matrix (reviewed in Karsenty and Olson 2016). Association of the activated androgen receptor (AR) with RUNX2 prevents binding of RUNX2 to the BGLAP promoter (Baniwal et al. 2009). When YAP1, tyrosine phosphorylated by SRC and/or YES1, binds to RUNX2 at the BGLAP gene promoter, transcription of the BGLAP gene is inhibited (Zaidi et al. 2004). Signaling by SRC is known to inhibit osteoblast differentiation (Marzia et al. 2000).

Simultaneous binding of RUNX2 and SP7 (Osterix, also known as OSX) to adjacent RUNX2 and SP7 binding sites, respectively, in the UCMA promoter, synergistically activates UCMA transcription. UCMA stimulates osteoblast differentiation and formation of mineralized nodules (Lee et al. 2015).

The SCF(SKP2) E3 ubiquitin ligase complex inhibits differentiation of osteoblasts by polyubiquitinating RUNX2 and targeting it for proteasome-mediated degradation (Thacker et al. 2016). This process is inhibited by glucose uptake in osteoblasts (Wei et al. 2015).

References

Baniwal SK, Khalid O, Sir D, Buchanan G, Coetzee GA & Frenkel B (2009). Repression of Runx2 by androgen receptor (AR) in osteoblasts and prostate cancer cells: AR binds Runx2 and abrogates its recruitment to DNA. Mol. Endocrinol., 23, 1203-14.

Lee YJ, Park SY, Lee SJ, Boo YC, Choi JY & Kim JE (2015). Ucma, a direct transcriptional target of Runx2 and Osterix, promotes osteoblast differentiation and nodule formation. Osteoarthr. Cartil., 23, 1421-31.

Marzia M, Sims NA, Voit S, Migliaccio S, Taranta A, Bernardini S, ... Teti A (2000). Decreased c-Src expression enhances osteoblast differentiation and bone formation. J. Cell Biol., 151, 311-20.

Thacker G, Kumar Y, Khan MP, Shukla N, Kapoor I, Kanaujiya JK, ... Trivedi AK (2016). Skp2 inhibits osteogenesis by promoting ubiquitin-proteasome degradation of Runx2. Biochim. Biophys. Acta, 1863, 510-9.

Zaidi SK, Sullivan AJ, Medina R, Ito Y, van Wijnen AJ, Stein JL, ... Stein GS (2004). Tyrosine phosphorylation controls Runx2-mediated subnuclear targeting of YAP to repress transcription. EMBO J., 23, 790-9.

Edit history

Date	Action	Author
2016-09-27	Authored	Orlic-Milacic M
2016-09-27	Created	Orlic-Milacic M
2017-08-04	Reviewed	Ducy P
2017-08-09	Edited	Orlic-Milacic M
2018-08-23	Modified	Schmidt EE

Elements found in this pathway

Input	UniProt Id	Input	UniProt Id	Input	UniProt Id
Runx2	Q13950-2, Q13950-1				

14. Activation of Matrix Metalloproteinases (R-HSA-1592389)

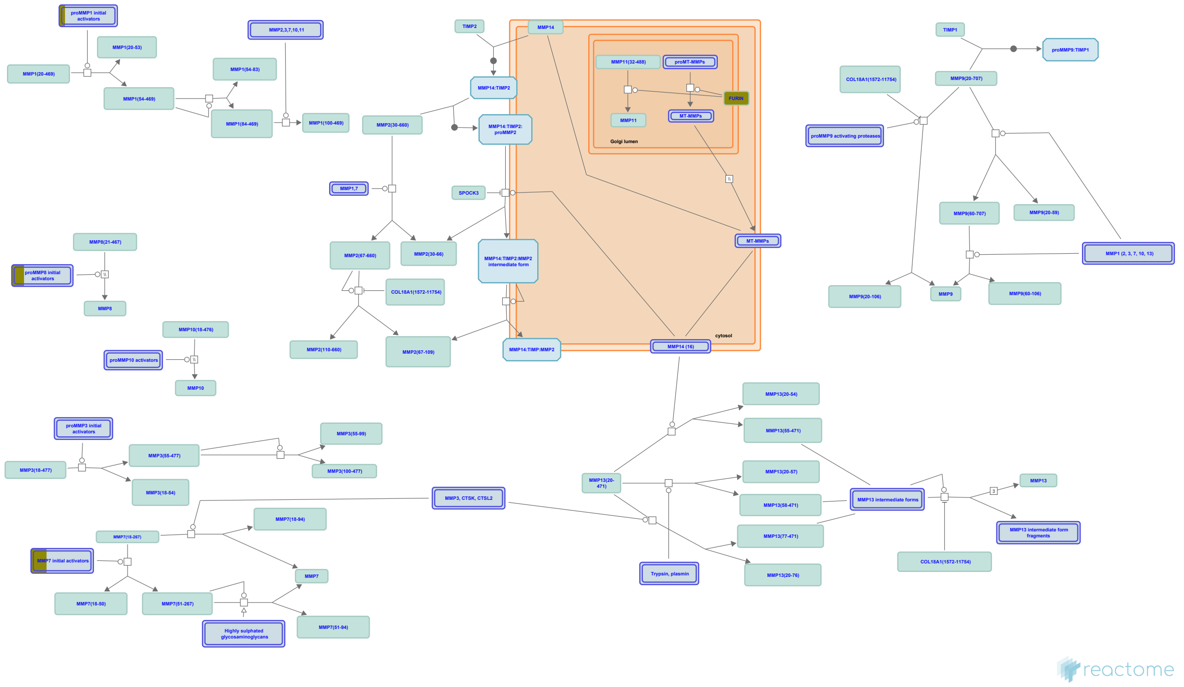

The matrix metalloproteinases (MMPs), previously known as matrixins, are classically known to be involved in the turnover of extracellular matrix (ECM) components. However, recent high throughput proteomics analyses have revealed that ~80% of MMP substrates are non-ECM proteins including cytokines, growth factor binding protiens, and receptors. It is now clear that MMPs regulate ECM turnover not only by cleaving ECM components, but also by the regulation of cell signalling, and that some MMPs are beneficial and may be drug anti-targets. Thus, MMPs have important roles in many processes including embryo development, morphogenesis, tissue homeostasis and remodeling. They are implicated in several diseases such as arthritis, periodontitis, glomerulonephritis, atherosclerosis, tissue ulceration, and cancer cell invasion and metastasis. All MMPs are synthesized as preproenzymes. Alternate splice forms are known, leading to nuclear localization of select MMPs. Most are secreted from the cell, or in the case of membrane type (MT) MMPs become plasma membrane associated, as inactive proenzymes. Their subsequent activation is a key regulatory step, with requirements specific to MMP subtype.

References

Woessner J & Nagase H (2000). *Matrix Metalloproteinases and TIMPs* .

Cauwe B, Van den Steen PE & Opdenakker G (2007). The biochemical, biological, and pathological kaleidoscope of cell surface substrates processed by matrix metalloproteinases. Crit Rev Biochem Mol Biol, 42, 113-85.

Butler GS & Overall CM (2009). Updated biological roles for matrix metalloproteinases and new "intracellular" substrates revealed by degradomics. Biochemistry, 48, 10830-45.

Morrison CJ, Butler GS, Rodríguez D & Overall CM (2009). Matrix metalloproteinase proteomics: substrates, targets, and therapy. Curr Opin Cell Biol, 21, 645-53.

Rodríguez D, Morrison CJ & Overall CM (2010). Matrix metalloproteinases: what do they not do? New substrates and biological roles identified by murine models and proteomics. Biochim Biophys Acta, 1803, 39-54.

Edit history

Date	Action	Author
2011-09-09	Authored	Jupe S
2011-09-09	Created	Jupe S
2012-02-21	Edited	Jupe S
2012-02-28	Reviewed	Butler GS, Overall CM
2018-08-24	Modified	Schmidt EE

Elements found in this pathway

Input	UniProt Id	Input	UniProt Id	Input	UniProt Id
Elane	P08246	Furin	P09958		

15. Expression and Processing of Neurotrophins (R-HSA-9036866)

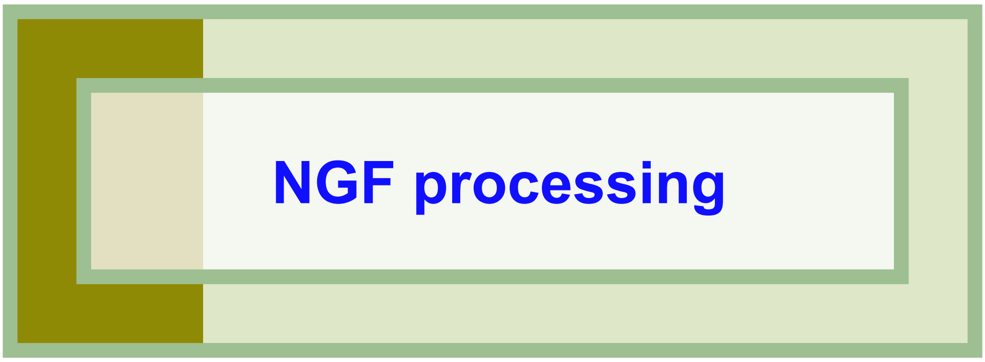

Neurotrophins (NGF, BDNF, NT-3, NT-4/5) play pivotal roles in survival, differentiation, and plasticity of neurons in the peripheral and central nervous system. They are produced, and secreted in minute amounts, by a variety of tissues. They signal through two types of receptors: TRK tyrosine kinase receptors (TRKA, TRKB, TRKC), which specifically interact with the different neurotrophins, and p75NTR, which interacts with all neurotrophins. Neurotrophins and their receptors are synthesized as several different splice variants, which differ in terms of their biological activities. For review, please refer to Lessmann et al. 2003, Chao 2003, and Park and Poo 2013.

References

Lessmann V, Gottmann K & Malcangio M (2003). Neurotrophin secretion: current facts and future prospects. Prog Neurobiol, 69, 341-74.

Chao MV (2003). Neurotrophins and their receptors: a convergence point for many signalling pathways. Nat Rev Neurosci, 4, 299-309.

Park H & Poo MM (2013). Neurotrophin regulation of neural circuit development and function. Nat. Rev. Neurosci., 14, 7-23.

Edit history

Date	Action	Author
2018-02-10	Edited	Orlic-Milacic M
2018-02-10	Authored	Orlic-Milacic M
2018-02-10	Created	Orlic-Milacic M
2018-08-23	Modified	Schmidt EE

Elements found in this pathway

Input	UniProt Id	Input	UniProt Id	Input	UniProt Id
Furin	P09958				

16. NGF processing (R-HSA-167060)

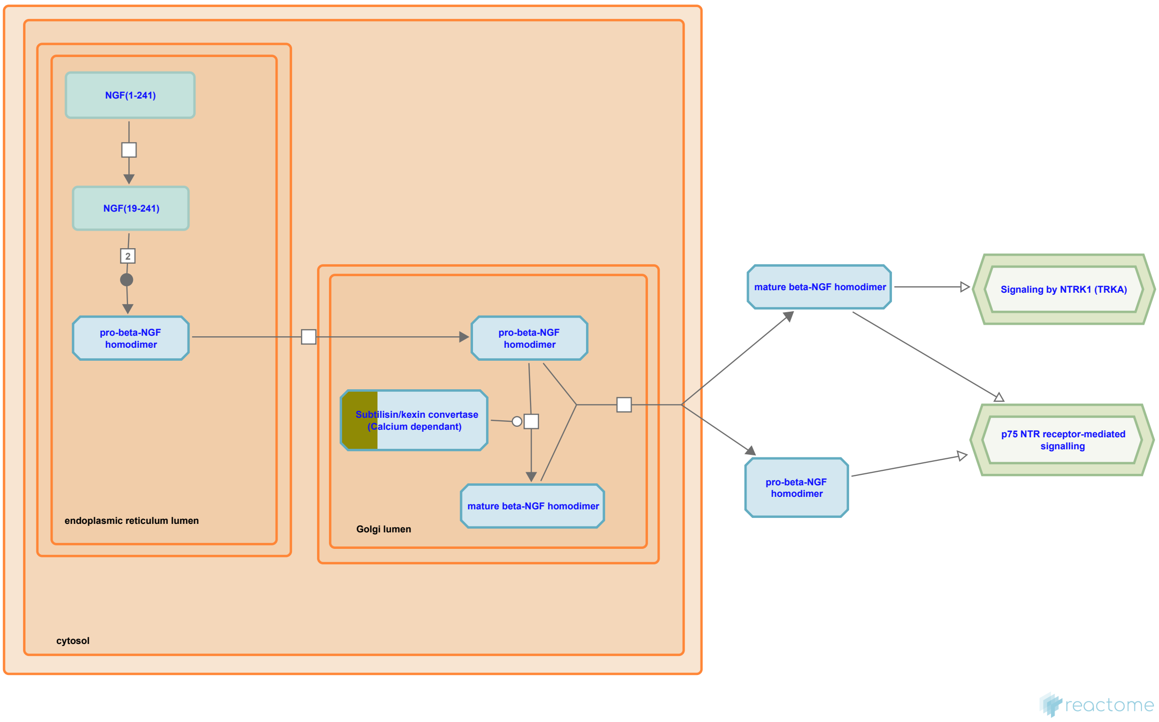

All neurotrophins (NTs) are generated as pre-pro-neurotrophin precursors. The signal peptide is cleaved off as NT is associated with the endoplasmic reticulum (ER). The resulting pro-NT can form a homodimer spontaneously which then transits to the Golgi apparatus and then onto the trans-Golgi network (TGN). Resident protein convertases (PCs) can cleave off the pro-sequence and mature NT is is targeted to constitutively released vesicles. The pro-NT form can also be released to the extracellular region.

References

Lessmann V, Gottmann K & Malcangio M (2003). Neurotrophin secretion: current facts and future prospects. Prog Neurobiol, 69, 341-74.

Edit history

Date	Action	Author
2006-02-07	Created	
2006-10-10	Edited	Jassal B
2006-10-10	Authored	Nasi S, Annibali D
2007-11-08	Reviewed	Greene LA
2018-08-23	Modified	Schmidt EE

Elements found in this pathway

Input	UniProt Id	Input	UniProt Id	Input	UniProt Id
Furin	P09958				

17. Muscarinic acetylcholine receptors (R-HSA-390648)

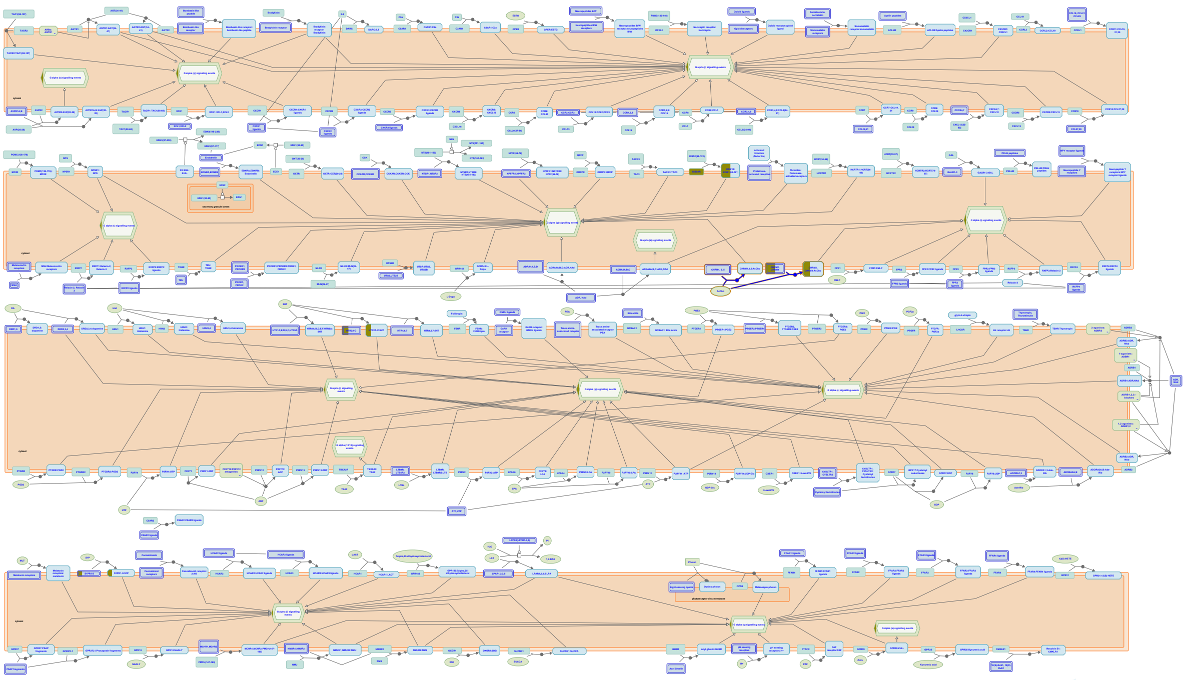

Muscarinic acetylcholine (mAChRs) receptors were so named because they are more sensitive to muscarine than to nicotine (Ishii M and Kurachi Y, 2006). Their counterparts are nicotinic acetylcholine receptors (nAChRs), ion channels receptors that are also important in the autonomic nervous system. Many drugs can manipulate these two distinct receptors by acting as selective agonists or antagonists. mAChRs bind to the bioamine acetylcholine, have a widespread tissue distribution and are involved in the control of numerous central and peripheral physiological responses, particularly voluntary muscle contraction. They are also major targets for drugs in human diseases such as Alzheimer's, Parkinson's and schizophrenia. This family of G-protein coupled receptors consists of five members designated M1-M5 and are sub-divided into two groups based on their primary coupling to G proteins. M2 and M4 receptors couple to Gi/o proteins and M1, M3 and M5 receptors couple to Gq/11 proteins (Caulfield MP and Birdsall NJ, 1998).

References

Caulfield MP & Birdsall NJ (1998). International Union of Pharmacology. XVII. Classification of muscarinic acetylcholine receptors. Pharmacol Rev, 50, 279-90.

Ishii M & Kurachi Y (2006). Muscarinic acetylcholine receptors. Curr Pharm Des, 12, 3573-81.

Edit history

Date	Action	Author
2009-02-10	Edited	Jassal B
2009-02-10	Authored	Jassal B
2009-02-10	Created	Jassal B
2009-03-02	Reviewed	D'Eustachio P
2018-08-23	Modified	Schmidt EE

Elements found in this pathway

Input	UniProt Id	Input	UniProt Id	Input	UniProt Id
Chrm4	P08173				

18. Alternative complement activation (R-HSA-173736)

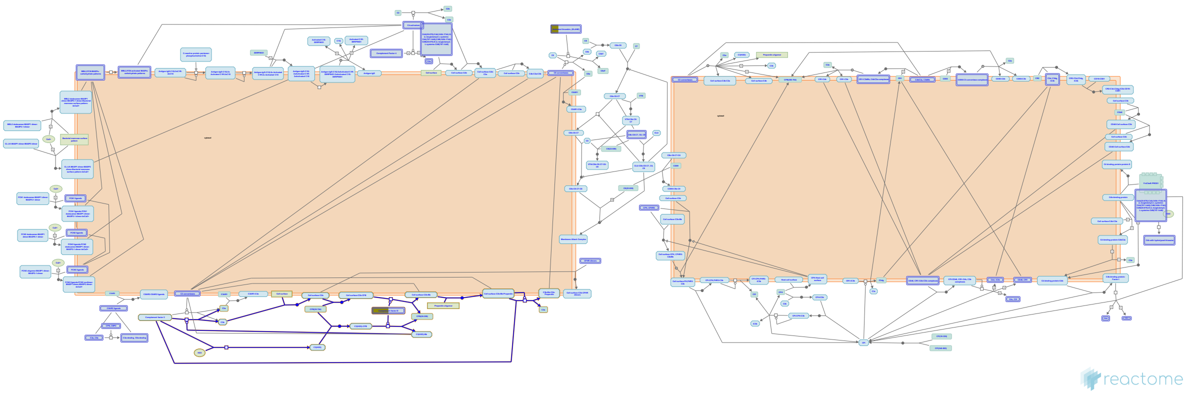

Cellular compartments: extracellular region, plasma membrane.

The proteins participating in alternative pathway activation are C3 (and C3b), the factors B, D, and properdin. In the first place, alternative pathway activation is a positive feedback mechanism to increase C3b. When C3b binds covalently to sugars on a cell surface, it can become protected. Then Factor B binds to C3b. In the presence of Factor D, bound Factor B is cleaved to Ba and Bb. Bb contains the active site for a C3 convertase. Properdin then binds to C3bBb to stabilize the C3bBb convertase on cell surface leading to cleavage of C3. Finally, a C3bBb3b complex forms and this is a C5 convertase.

References

Fearon DT (1979). Activation of the alternative complement pathway. CRC Crit Rev Immunol, 1, 1-32.

Edit history

Date	Action	Author
2004-08-04	Authored	de Bono B
2006-02-16	Created	de Bono B
2006-07-04	Reviewed	D'Eustachio P
2010-10-22	Revised	Jupe S
2010-11-17	Edited	Jupe S
2018-08-23	Modified	Schmidt EE

Elements found in this pathway

Input	UniProt Id	Input	UniProt Id	Input	UniProt Id
Cfd	P00746				

19. RUNX2 regulates bone development (R-HSA-8941326)

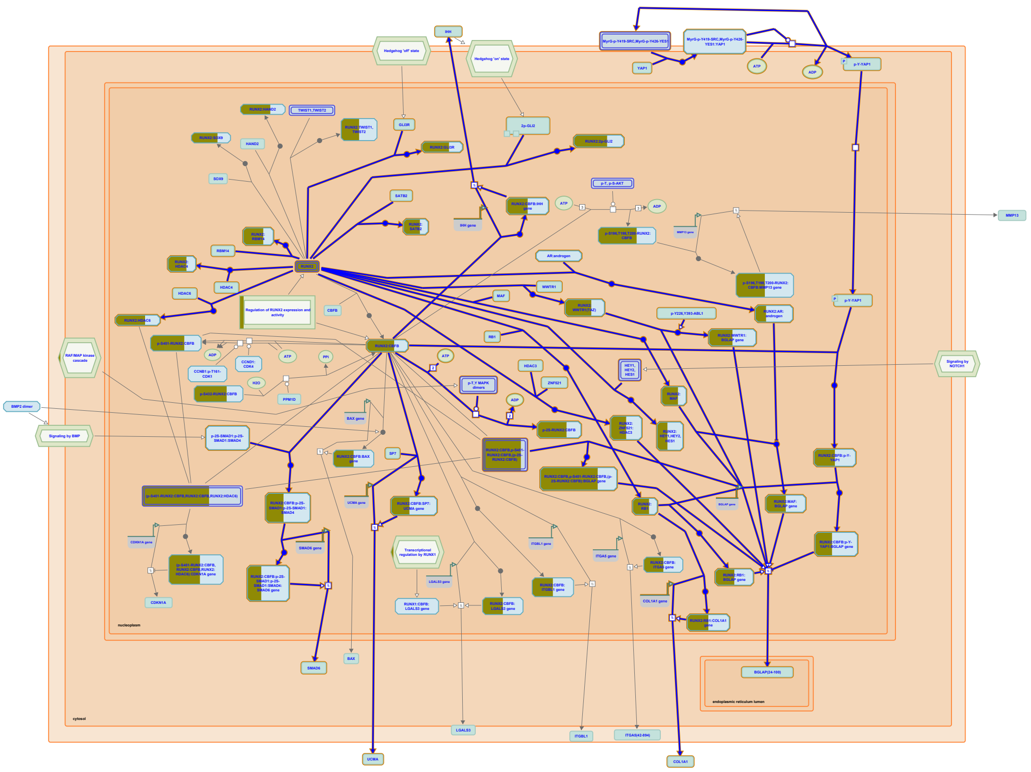

RUNX2 is required for the development of both intramembraneous and endochondral bones through regulation of osteoblast differentiation and chondrocyte maturation, respectively. In its absence, intramembraneous ossification is blocked while endochondral ossification is arrested at the cartilaginous stage (Otto et al. 1997, Komori et al. 1997). In mice and humans, RUNX2 haploinsufficiency causes Cleidocranial dysplasia, a generalized bone disorder (Otto et al. 1997, Lee et al. 1997).

RUNX2 stimulates transcription of most of the genes constituting the bone extracellular matrix and of BGLAP gene, which encodes Osteocalcin, a bone-derived hormone controlling glucose metabolism, male fertility and cognition (Ducy et al. 1997).

RUNX2 promotes chondrocyte maturation by stimulating transcription of the IHH gene, encoding Indian hedgehog (Takeda et al. 2001, Yoshida et al. 2004).

In response to BMP2 signaling, RUNX2 forms a complex with SMAD1:SMAD4 heterotrimer in the nucleus and stimulates transcription of SMAD6 (Wang et al. 2007).

RBM14, a negative regulator of RUNX2 transcriptional activity, is frequently overexpressed in osteosarcoma (Li et al. 2009).

References

Yoshida CA, Yamamoto H, Fujita T, Furuichi T, Ito K, Inoue K, ... Komori T (2004). Runx2 and Runx3 are essential for chondrocyte maturation, and Runx2 regulates limb growth through induction of Indian hedgehog. Genes Dev., 18, 952-63.

Li X, Hoeppner LH, Jensen ED, Gopalakrishnan R & Westendorf JJ (2009). Co-activator activator (CoAA) prevents the transcriptional activity of Runt domain transcription factors. J. Cell. Biochem., 108, 378-87.

Lee B, Thirunavukkarasu K, Zhou L, Pastore L, Baldini A, Hecht J, ... Karsenty G (1997). Missense mutations abolishing DNA binding of the osteoblast-specific transcription factor OSF2/CBFA1 in cleidocranial dysplasia. Nat. Genet., 16, 307-10.

Otto F, Thornell AP, Crompton T, Denzel A, Gilmour KC, Rosewell IR, ... Owen MJ (1997). Cbfa1, a candidate gene for cleidocranial dysplasia syndrome, is essential for osteoblast differentiation and bone development. Cell, 89, 765-71.

Komori T, Yagi H, Nomura S, Yamaguchi A, Sasaki K, Deguchi K, ... Kishimoto T (1997). Targeted disruption of Cbfa1 results in a complete lack of bone formation owing to maturational arrest of osteoblasts. Cell, 89, 755-64.

Edit history

Date	Action	Author
2016-09-30	Authored	Orlic-Milacic M
2016-09-30	Created	Orlic-Milacic M
2017-08-04	Reviewed	Ducy P
2017-08-09	Edited	Orlic-Milacic M
2018-08-23	Modified	Schmidt EE

Elements found in this pathway

Input	UniProt Id	Input	UniProt Id	Input	UniProt Id
Runx2	Q13950-2, Q13950-1				

20. Elastic fibre formation (R-HSA-1566948)

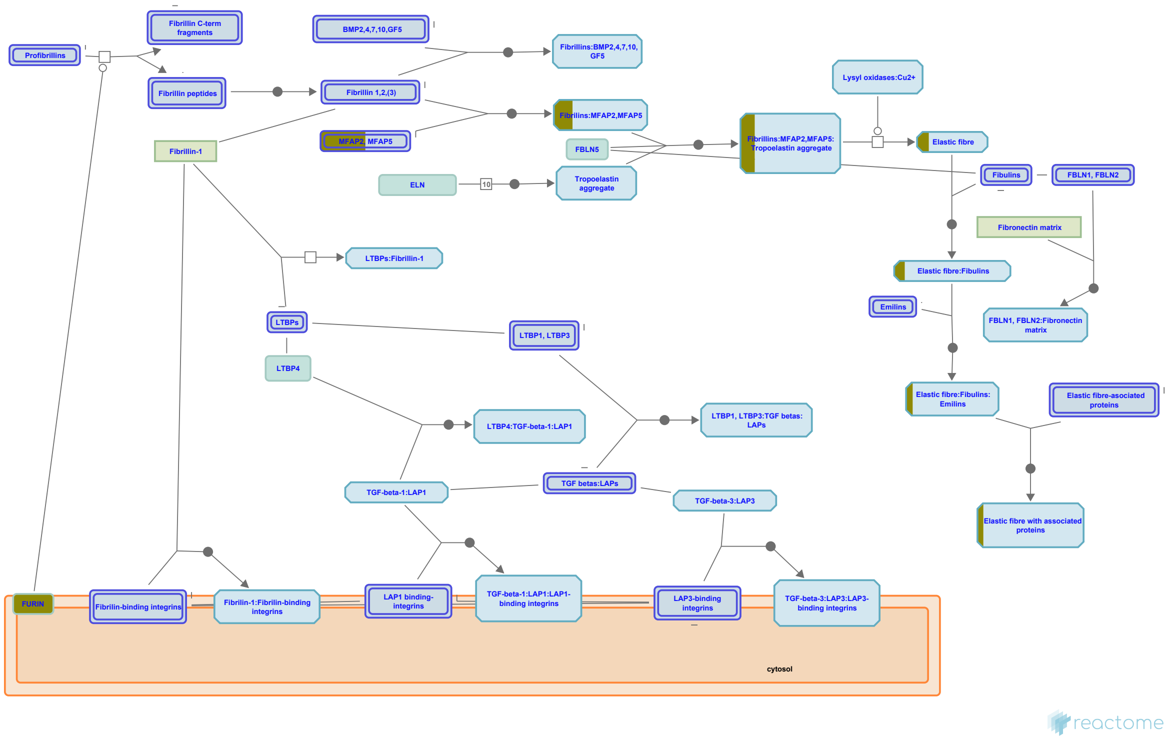

Cellular compartments: extracellular region.

Elastic fibres (EF) are a major structural constituent of dynamic connective tissues such as large arteries and lung parenchyma, where they provide essential properties of elastic recoil and resilience. EF are composed of a central cross-linked core of elastin, surrounded by a mesh of microfibrils, which are composed largely of fibrillin. In addition to elastin and fibrillin-1, over 30 ancillary proteins are involved in mediating important roles in elastic fibre assembly as well as interactions with the surrounding environment. These include fibulins, elastin microfibril interface located proteins (EMILINs), microfibril-associated glycoproteins (MAGPs) and Latent TGF-beta binding proteins (LTBPs). Fibulin-5 for example, is expressed by vascular smooth muscle cells and plays an essential role in the formation of elastic fibres through mediating interactions between elastin and fibrillin (Yanigasawa et al. 2002, Freeman et al. 2005). In addition, it plays a role in cell adhesion through integrin receptors and has been shown to influence smooth muscle cell proliferation (Yanigasawa et al. 2002, Nakamura et al. 2002). EMILINs are a family of homologous glycoproteins originally identified in extracts of aortas. Found at the elastin-fibrillin interface, early studies showed that antibodies to EMILIN can affect the process of elastic fibre formation (Bressan et al. 1993). EMILIN1 has been shown to bind elastin and fibulin-5 and appears to coordinate their common interaction (Zanetti et al. 2004). MAGPs are found to co-localize with microfibrils. MAGP-1, for example, binds strongly to an N-terminal sequence of fibrillin-1. Other proteins found associated with microfibrils include vitronectin (Dahlback et al. 1990).

Fibrillin is most familiar as a component of elastic fibres but microfibrils with no elastin are found in the ciliary zonules of the eye and invertebrate circulatory systems. The addition of elastin to microfibrils is a vertebrate adaptation to high pulsatile pressures in their closed circulatory systems (Faury et al. 2003). Elastin appears to have emerged after the divergence of jawless vertebrates from other vertebrates (Sage 1982).

Fibrillin-1 is the major structural component of microfibrils. Fibrillin-2 is expressed earlier in development than fibrillin-1 and may be important for elastic fiber formation (Zhang et al. 1994). Fibrillin-3 arose as a duplication of fibrillin-2 that did not occur in the rodent lineage. It was first isolated from human brain (Corson et al. 2004).

Fibrillin assembly is not as well defined as elastin assembly. The primary structure of fibrillin is dominated by calcium binding epidermal growth factor like repeats (Kielty et al. 2002). Fibrillin may form dimers or trimers before secretion. However, multimerisation predominantly occurs outside the cell. Formation of fibrils appears to require cell surface structures suggesting an involvement of cell surface receptors. Fibrillin is assembled pericellularly (i.e. on or close to the cell surface) into microfibrillar arrays that undergo time dependent maturation into microfibrils with beaded-string appearance. Transglutaminase forms gamma glutamyl epsilon lysine isopeptide bonds within or between peptide chains. Additionally, intermolecular disulfide bond formation between fibrillins is an important contributor to fibril maturation (Reinhardt et al. 2000).

Models of fibrillin-1 microfibril structure suggest that the N-terminal half of fibrillin-1 is asymmetrically exposed in outer filaments, while the C-terminal half is buried in the interior (Kuo et al. 2007). Fibrillinopathies include Marfan syndrome, familial ectopia lentis, familial thoracic aneurysm, all due to mutations in the fibrillin-1 gene FBN1, and congenital contractural arachnodactyly which is caused by mutation of FBN2 (Maslen & Glanville 1993, Davis & Summers 2012).

In vivo assembly of fibrillin requires the presence of extracellular fibronectin fibres (Sabatier et al. 2009). Fibrillins have Arg-Gly-Asp (RGD) sequences that interact with integrins (Pfaff et al. 1996, Sakamoto et al. 1996, Bax et al., 2003, Jovanovic et al. 2008) and heparin-binding domains that interact with a cell-surface heparan sulfate proteoglycan (Tiedemann et al. 2001) possibly a syndecan (Ritty et al. 2003). Fibrillins also have a major role in binding and sequestering growth factors such as TGF beta into the ECM (Neptune et al. 2003). Proteoglycans such as versican (Isogai et al. 2002), biglycan, and decorin (Reinboth et al. 2002) can interact with the microfibrils. They confer specific properties including hydration, impact absorption, molecular sieving, regulation of cellular activities, mediation of growth factor association, and release and transport within the extracellular matrix (Buczek-Thomas et al. 2002). In addition, glycosaminoglycans have been shown to interact with tropoelastin through its lysine side chains (Wu et al. 1999), regulating tropoelastin assembly (Tu & Weiss 2008).

Elastin is synthesized as a 70kDa monomer called tropoelastin, a highly hydrophobic protein composed largely of two types of domains that alternate along the polypeptide chain. Hydrophobic domains are rich in glycine, proline, alanine, leucine and valine. These amino acids occur in characteristic short (3-9 amino acids) tandem repeats, with a flexible and highly dynamic structure (Floquet et al. 2004). Unlike collagen, glycine in elastin is not rigorously positioned every 3 residues. However, glycine is distributed frequently throughout all hydrophobic domains of elastin, and displays a strong preference for inter-glycine spacing of 0-3 residues (Rauscher et al. 2006).

Elastic fibre formation involves the deposition of tropoelastin onto a template of fibrillin rich microfibrils. Recent results suggest that the first step of elastic fiber formation is the organization of small globules of elastin on the cell surface followed by globule aggregation into microfibres (Kozel et al. 2006). An important contribution to the initial stages assembly is thought to be made by the intrinsic ability of the protein to direct its own polymeric organization in a process termed 'coacervation' (Bressan et al. 1986). This self-assembly process appears to be determined by interactions between hydrophobic domains (Bressan et al. 1986, Vrhovski et al. 1997, Bellingham et al. 2003, Cirulis & Keeley 2010) which result in alignment of the cross-linking domains, allowing the stabilization of elastin through the formation of cross-links generated through the oxidative deamination of lysine residues, catalyzed by members of the lysyl oxidase (LOX) family (Reiser et al. 1992, Mithieux & Weiss 2005). The first step in the cross-linking reaction is the oxidative formation of the delta aldehyde, known as alpha aminoadipic semialdehyde or allysine (Partridge 1963). Subsequent reactions that are probably spontaneous lead to the formation of cross-links through dehydrolysinonorleucine and allysine aldol, a trifunctional cross-link dehydromerodesmosine and two tetrafunctional cross-links desmosine and isodesmosine (Lucero & Kagan 2006), which are unique to elastin. These cross-links confer mechanical integrity and high durability. In addition to their role in self-assembly, hydrophobic domains provide elastin with its elastomeric properties, with initial studies suggesting that the elastomeric propereties of elastin are driven through changes in entropic interactions with surrounding water molecules (Hoeve & Flory 1974).

A very specific set of proteases, broadly grouped under the name elastases, is responsible for elastin remodelling (Antonicelli et al. 2007). The matrix metalloproteinases (MMPs) are particularly important in elastin breakdown, with MMP2, 3, 9 and 12 explicitly shown to degrade elastin (Ra & Parks 2007). Nonetheless, elastin typically displays a low turnover rate under normal conditions over a lifetime (Davis 1993).

References

Kozel BA, Rongish BJ, Czirok A, Zach J, Little CD, Davis EC, ... Mecham RP (2006). Elastic fiber formation: a dynamic view of extracellular matrix assembly using timer reporters. J Cell Physiol , 207, 87-96.

Edit history

Date	Action	Author
2011-09-06	Created	Jupe S
2012-04-30	Authored	Jupe S
2012-11-02	Reviewed	Muiznieks LD
2012-11-12	Edited	Jupe S
2013-02-27	Reviewed	Parkinson J
2018-08-23	Modified	Schmidt EE

Elements found in this pathway

Input	UniProt Id	Input	UniProt Id	Input	UniProt Id
Furin	P09958	Mfap2	P55001		

21. Intestinal saccharidase deficiencies (R-HSA-5659898)

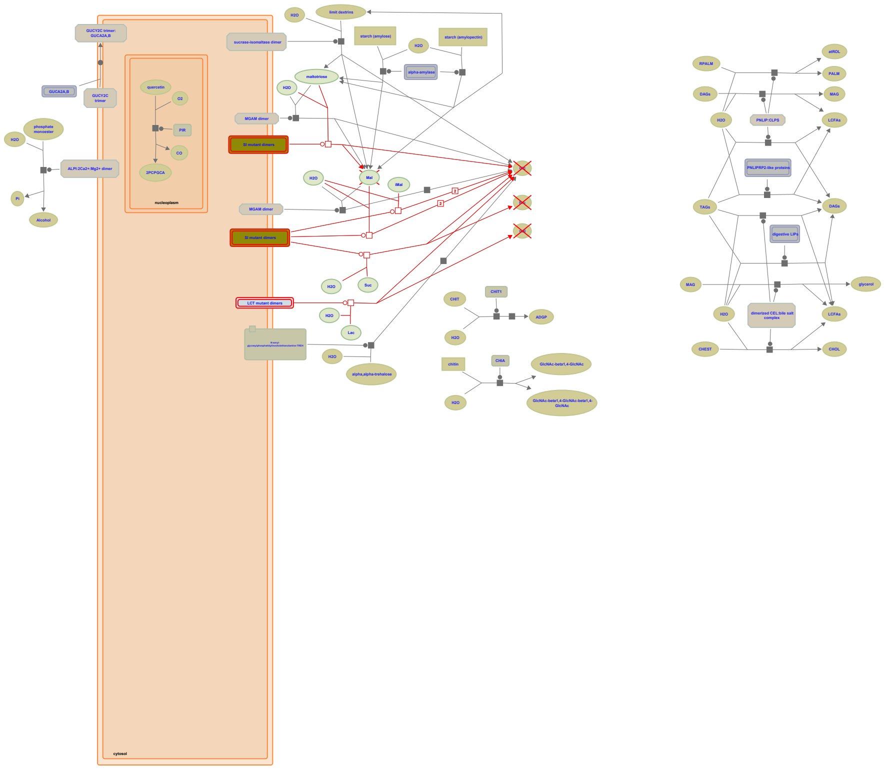

Diseases: intestinal disaccharidase deficiency.

Defects in in two enzymes required for intestinal digestion of dietary carbohydrate, lactase (LCT, a domain of lactase-phlorizin hydrolase protein) and sucrase-isomaltase (SI), are annotated here. The first affects nursing infants; the second affects individuals after weaning.

The disaccharide lactose is a major constituent of human breast milk. To be taken up from the gut in the nursing infant, this sugar must first be hydrolyzed by LCT present on the external face of enterocytes in microvilli of the small intestine. Mutations that disrupt LCT activity are associated with acute illness in newborn children as lactose fermentation by gut bacteria leads to severe diarrhea. The condition is effectively treated by feeding affected infants a lactose-free formula. This congenital disease is distinct from the down-regulation of LCT expression after weaning in many human populations that is associated with a milder form of lactose intolerance in adults (Jarvela et al. 2009).

The starch in a post-weaning diet is digested by amylases to di- and oligosaccharides that must be further digested to monosaccharides in order to be taken up from the lumen of the small intestine into endothelial cells of the intestinal brush border. If they are not digested, a process in which enterocyte-associated SI plays a central role, they remain in the gut lumen and are fermented by gut bacteria, leading to osmotic and fermentative diarrhea (Naim et al. 2012; Van Beers et al. 1995).

References

Järvelä I, Torniainen S & Kolho KL (2009). Molecular genetics of human lactase deficiencies. Ann. Med., 41, 568-75.

Naim HY, Heine M & Zimmer KP (2012). Congenital sucrase-isomaltase deficiency: heterogeneity of inheritance, trafficking, and function of an intestinal enzyme complex. J. Pediatr. Gastroenterol. Nutr., 55, S13-20.

Van Beers EH, Büller HA, Grand RJ, Einerhand AW & Dekker J (1995). Intestinal brush border glycohydrolases: structure, function, and development. Crit. Rev. Biochem. Mol. Biol., 30, 197-262.

Edit history

Date	Action	Author
2014-12-24	Created	D'Eustachio P
2015-01-29	Reviewed	Jassal B
2015-02-15	Edited	D'Eustachio P
2015-02-15	Reviewed	Amiri M, Naim HY
2015-02-15	Authored	D'Eustachio P
2017-03-06	Modified	D'Eustachio P

Elements found in this pathway

Input	UniProt Id	Input	UniProt Id	Input	UniProt Id
Sis	P14410				

22. Carboxyterminal post-translational modifications of tubulin (R-HSA-8955332)

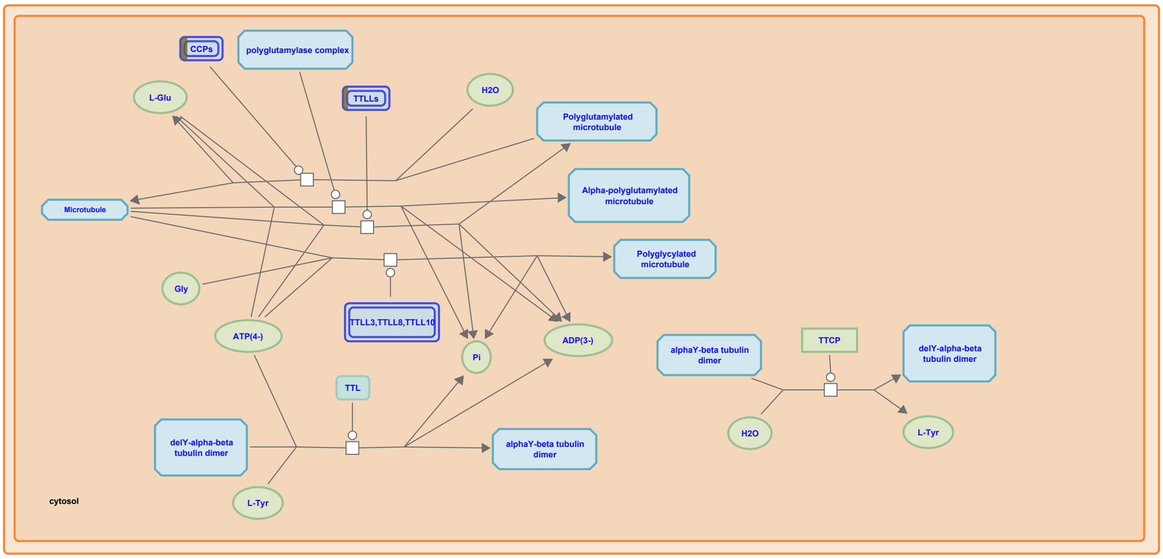

Cellular compartments: cytosol.

Tubulins fold into compact globular domains with less structured carboxyterminal tails. These tails vary in sequence between tubulin isoforms and are exposed on the surfaces of microtubules. They can undergo a variety of posttranslational modifications, including the attachment and removal of polyglutamate chains and in the case of alpha-tunulins the loss and reattachment of a terminal tyrosine (Tyr) residue. These modifications are associated with changes in the rigidity and stability of microtubules (Song & Brady 2015; Yu et al. 2015).

Mutations affecting these modification processes can have severe effects on phenotype (e.g., Ikegami et al. 2007). Nevertheless, the precise molecular mechanisms by which these changes in tubulin structure modulate its functions remain unclear, so these modification processes are simply annotated here as a series of chemical transformations of tubulins.

References

Ikegami K, Heier RL, Taruishi M, Takagi H, Mukai M, Shimma S, ... Setou M (2007). Loss of alpha-tubulin polyglutamylation in ROSA22 mice is associated with abnormal targeting of KIF1A and modulated synaptic function. Proc. Natl. Acad. Sci. U.S.A., 104, 3213-8.

Song Y & Brady ST (2015). Post-translational modifications of tubulin: pathways to functional diversity of microtubules. Trends Cell Biol., 25, 125-36.

Yu I, Garnham CP & Roll-Mecak A (2015). Writing and Reading the Tubulin Code. J. Biol. Chem., 290, 17163-72.

Edit history

Date	Action	Author
2017-01-11	Created	D'Eustachio P
2017-01-13	Authored	D'Eustachio P
2017-01-19	Edited	Jupe S

Date	Action	Author
2017-01-19	Reviewed	Jassal B
2018-08-23	Modified	Schmidt EE

Elements found in this pathway

Input	UniProt Id	Input	UniProt Id	Input	UniProt Id
Agbl2	Q5U5Z8	Ttll9	Q3SXZ7		

23. Removal of aminoterminal propeptides from gamma-carboxylated proteins (R-HSA-159782)

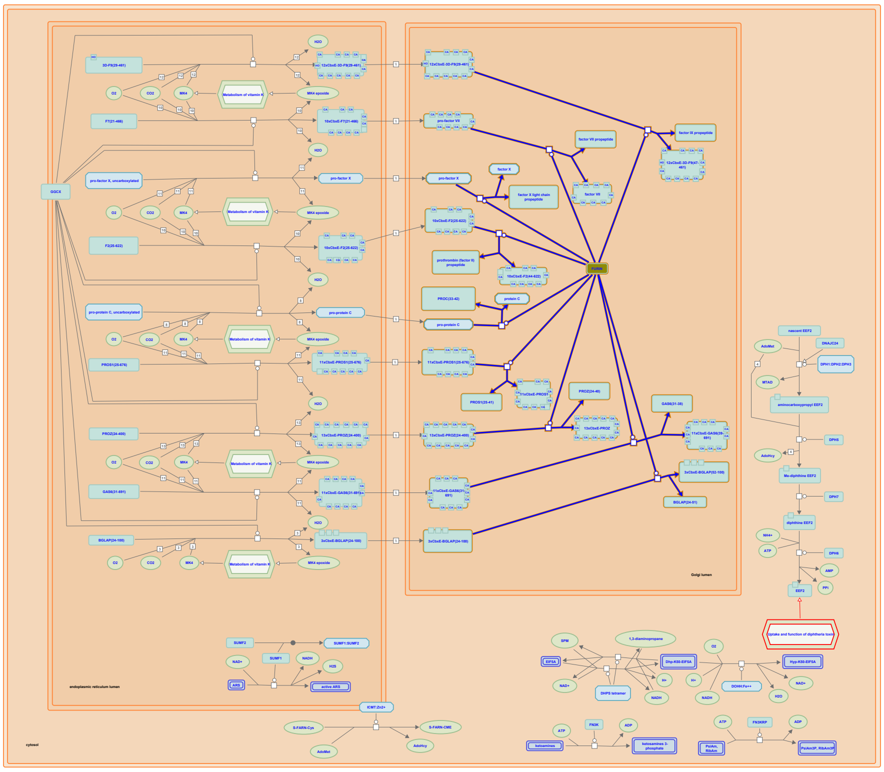

Cellular compartments: Golgi lumen.

Furin is an endopeptidase localized to the Golgi membrane that cleaves many proteins on the carboxyterminal side of the sequence motif Arg-[any residue]-(Lys or Arg)-Arg (Jones et al. 1995; Leduc et al. 1992). In the case of gamma-carboxylated proteins, if this cleavage does not occur, the proteins are still secreted but do not function properly (Bristol et al. 1993; Lind et al. 1997; Wasley et al. 1993). The aminoterminal fragments, "propeptides", generated in this reaction have no known function; the carboxylated, cleaved proteins are delivered to the cell membrane or secreted from the cell.

References

Leduc R, Molloy S S, Thorne BA & Thomas G (1992). Activation of human furin precursor processing endoprotease occurs by an intramolecular autoproteolytic cleavage. J Biol Chem, 267, 14304-8.

Jones BG, Thomas L, Molloy S S, Thulin CD, Fry MD, Walsh KA & Thomas G (1995). Intracellular trafficking of furin is modulated by the phosphorylation state of a casein kinase II site in its cytoplasmic tail. EMBO J, 14, 5869-83.

Lind B, Johnsen AH & Thorsen S (1997). Naturally occurring Arg(-1) to His mutation in human protein C leads to aberrant propeptide processing and secretion of dysfunctional protein C. Blood, 89, 2807-16.

Bristol JA, Furie BC & Furie B (1993). Propeptide processing during factor IX biosynthesis. Effect of point mutations adjacent to the propeptide cleavage site. J Biol Chem, 268, 7577-84.

Wasley LC, Rehemtulla A, Bristol JA & Kaufman RJ (1993). PACE/furin can process the vitamin K-dependent pro-factor IX precursor within the secretory pathway. J. Biol. Chem., 268, 8458-65.

Edit history

Date	Action	Author
2005-03-17	Authored	D'Eustachio P
2005-03-17	Created	D'Eustachio P
2018-08-21	Edited	D'Eustachio P
2018-08-30	Modified	Croft D

Elements found in this pathway

Input	UniProt Id	Input	UniProt Id	Input	UniProt Id
Furin	P09958				

24. Neurotransmitter uptake and metabolism In glial cells (R-HSA-112313)

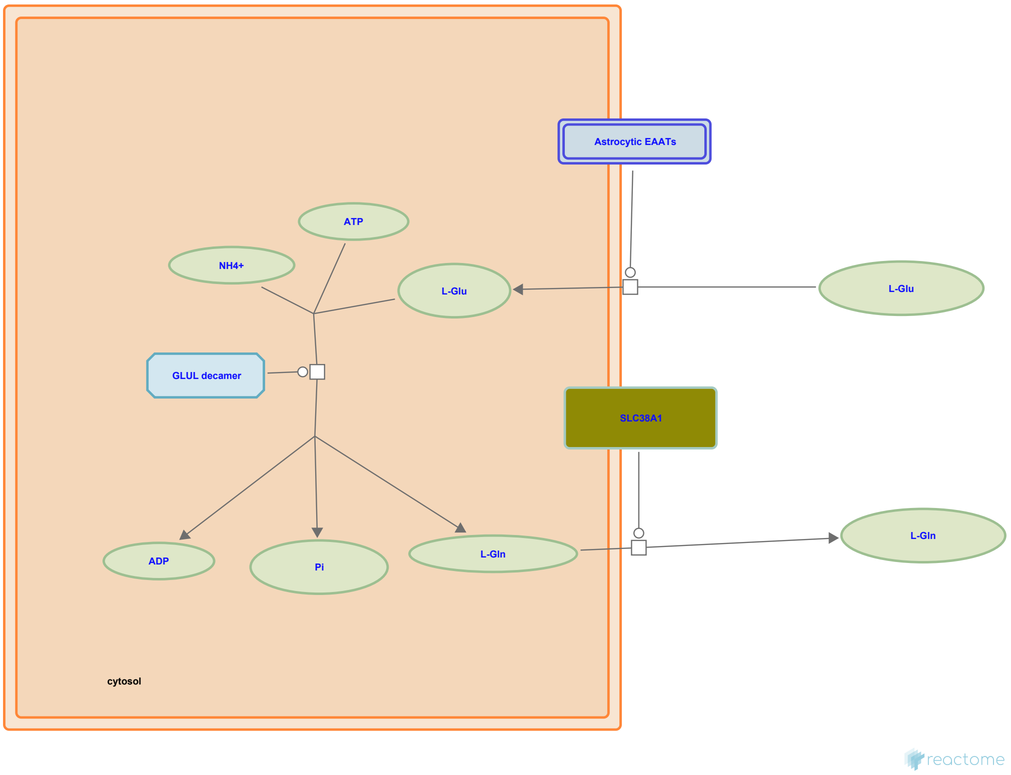

Neuotransmitter uptake by astrocytes is mediated by a specific transporter located on the astrocytic membrane. The imported neurotransmitter is metabolized and transported back to the neuron.

Edit history

Date	Action	Author
2004-04-22	Created	Joshi-Tope G
2008-01-14	Edited	Mahajan SS
2008-01-14	Authored	Mahajan SS
2008-12-02	Reviewed	Restituito S, Kavalali E
2018-08-24	Modified	Schmidt EE

Elements found in this pathway

Input	UniProt Id	Input	UniProt Id	Input	UniProt Id
Nat2	Q9H2H9				

25. Astrocytic Glutamate-Glutamine Uptake And Metabolism (R-HSA-210455)

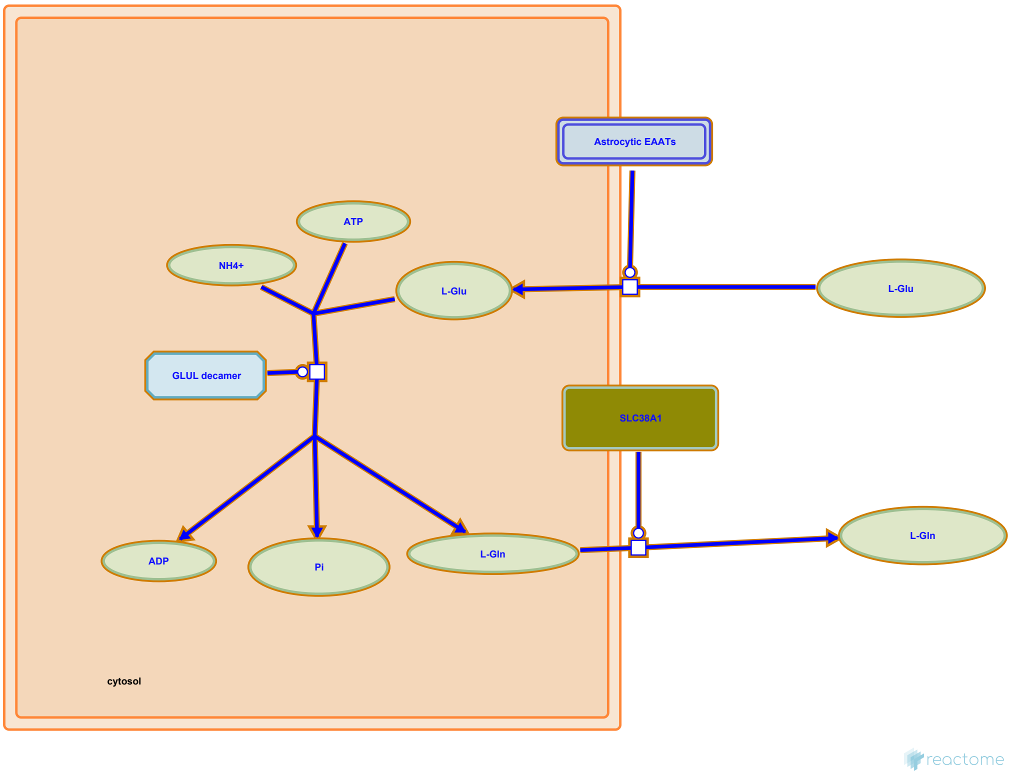

Cellular compartments: cytosol.

In astrocytic glutamate-glutamine cycle, the excess glutamate released by the pre-synaptic neuron in the synaptic cleft is transported into the astrocyte by a family glutamate transporters called the excitatory amino acid transporters 1 and 2, EAAT1 and EAAT2. Astrocytes carrying these transporters exist in close apposition to the synapse to clear excess glutamate to prevent excessive activation of neurons and hence neuronal death. Glutamate in astrocytes is converted to glutamine by glutamine synthetase. Glutamine is then transported into the extracellular space by system N transporters. The glutamate in the extracellular space is available for neuronal uptake.

Edit history

Date	Action	Author
2008-01-14	Edited	Mahajan SS
2008-01-14	Authored	Mahajan SS
2008-01-14	Created	Mahajan SS
2008-04-24	Reviewed	Kavalali E
2018-08-24	Modified	Schmidt EE

Elements found in this pathway

Input	UniProt Id	Input	UniProt Id	Input	UniProt Id
Nat2	Q9H2H9				

6. Identifiers found

Input	UniProt Id	Input	UniProt Id	Input	UniProt Id
Agbl2	Q5U5Z8	Alg13	Q9NP73	Arid3a	Q99856
Cfd	P00746	Chrm4	P08173	Elane	P08246
Fes	P07332	Furin	P09958	Mfap2	P55001
Nat2	Q9H2H9	Runx2	Q13950-2, Q13950-1	Sema3a	Q14563
Sis	P14410	Ttll9	Q3SXZ7		

Input	Ensembl Id	Input	Ensembl Id	Input	Ensembl Id
Arid3a	ENSG00000116017	Runx2	ENSG00000124813		

7. Identifiers not found

1810034E14Rik	4930563M21Rik	6820408C15Rik	Aaed1	Ahnak	Atl3	Bsdc1	Crocc
Cyp4a12b	Cyp4a30b	D330050G23Rik	Dnttip1	Efr3b	Espn	Fnbp4	Gm10081
Gm13178	Gm14164	Hes2	Kri1	Lrch1	Mir680-2	Mir6999	Nrsn2
Nudcd1	Oas1c	Oas1e	Olfr478	Olfr479	Olfr480	Olfr655	Olfr656
Olfr657	Olfr715	Olfr908	Olfr910	Olfr911-ps1	Pdrg1	Ppp1r3a	R3hdm4
Reg1	Reg2	Rem2	Rims3	Rnf183	Scaper	Scgb1a1	Sox12
Spint5	Stil	Tmco5	Vmn1r42	Vmn1r43	Wfdc3	Xkr7	Zbtb8b
Zcchc3	Zfp106						
