# Supplementary figures and images for: p.E95K mutation in Indian hedgehog causing brachydactyly type A1 impairs IHH/Gli1 downstream transcriptional regulation
Source: BMC Genet. 2019 Jan 16;20:10. doi: 10.1186/s12863-018-0697-5 (PMC6335781; doi:10.1186/s12863-018-0697-5)

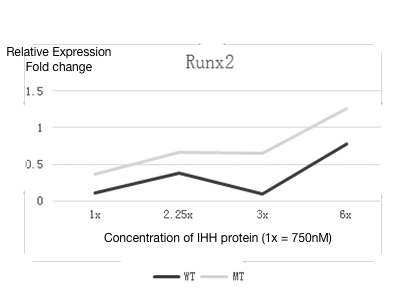

Supplement: Supplementary file 9 — Relative expression of Runx2 with IHH concentration increasing. Relative expression of Runx2 with increased IHH concentrations in WT and MT group compared to control group. (TIFF 489 kb) [file 12863_2018_697_MOESM9_ESM.tiff]
